# Dual‐Targeted Biomimetic MoSe_2_ Nanozymes for Enhancing Peri‐Implant Soft Tissue Integration

**DOI:** 10.1002/advs.77771

**Published:** 2026-09-21

**Authors:** Minghao Zhou, Miaomiao Tian, Jingwei Yu, Jiaxin Kang, Miaomiao Chen, Yue Yuan, Lizhi Liu, Hongbo Wei

**Affiliations:** ^1^ State Key Laboratory of Oral & Maxillofacial Reconstruction and Regeneration National Clinical Research Center for Oral Diseases Shaanxi Engineering Research Center for Dental Materials and Advanced Manufacture Department of Oral Implants School of Stomatology The Fourth Military Medical University Xi'an Shaanxi China; ^2^ State Key Laboratory of Solidification Processing School of Materials Science and Engineering Northwestern Polytechnical University Xi'an China

**Keywords:** biomimetic nanotechnology, cell membrane coating, dental implants, molybdenum diselenide, soft tissue integration

## Abstract

Peri‐implant diseases (PID) remain major threats to titanium implant longevity, largely due to insufficient soft tissue integration (STI), which weakens the biological seal and promotes a pathogenic–immunological amplification loop involving early *Staphylococcus aureus* colonization and oxidative stress‐induced macrophage dysfunction. Here, we developed a dual‐targeting biomimetic nanozyme platform by camouflaging molybdenum diselenide (MoSe_2_) nanoflowers with membranes derived from *S. aureus*‐pre‐stimulated macrophages (SPMM). This biomimetic design couples *S. aureus*–targeting of SPMM with the photothermal activity of MoSe_2_ to achieve targeted contact‐enhanced bacterial eradication, while integrating macrophage‐targeting of SPMM with reactive oxygen species (ROS)‐scavenging activity of MoSe_2_ to alleviate oxidative stress in macrophages. By disrupting the pathogenic–immunological amplification loop, MoSe_2_@SPMM reshaped a pro‐regenerative peri‐implant immune microenvironment. In a rat implantation model, near‐infrared (NIR)‐triggered MoSe_2_@SPMM suppressed bacterial colonization, promoted macrophage reprogramming, and preserved endothelial and fibroblast functions, thereby enhancing angiogenesis, matrix remodeling, connective tissue attachment, and integrin‐mediated tissue–implant adhesion. This biomimetic nanozyme strategy provides a rational framework for coordinating pathogen control and immune regulation to promote peri‐implant STI.

## Graphical abstract

A dual‐targeting biomimetic nanozyme, MoSe_2_@SPMM, selectively targets Staphylococcus aureus and oxidatively stressed macrophages. Near‐infrared activation couples contact‐enhanced photothermal bacterial eradication with reactive oxygen species scavenging to disrupt the pathogenic–immunological amplification loop. A pro‐regenerative immune microenvironment is thereby restored, promoting angiogenesis, matrix remodeling, connective tissue attachment, and integrin‐mediated peri‐implant soft tissue adhesion.

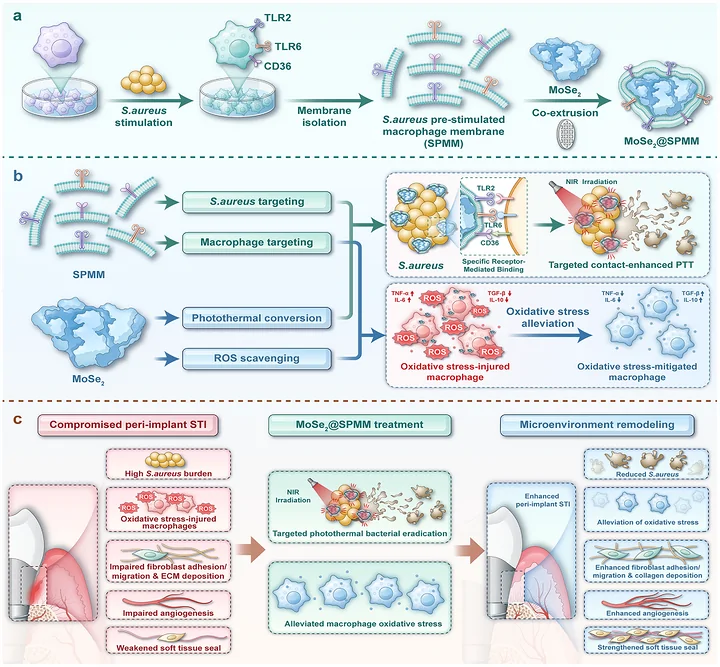

## 1 Introduction

Although titanium implants have achieved favorable osseointegration and widespread clinical adoption, their long‐term success is still threatened by peri‐implant diseases (PID), including peri‐implant mucositis and peri‐implantitis. Epidemiological data indicate that 17–24% of patients receiving implant therapy develop peri‐implantitis, while 41–51% exhibit peri‐implant mucositis worldwide [1]. These conditions not only impose sustained physical and psychological distress on patients but also contribute to a rapidly growing economic burden. In particular, the global expenditure for peri‐implantitis treatment alone reached USD 1.04 billion in 2024 and is projected to grow at a compound annual growth rate of 9.1% from 2025 to 2030 [2]. Increasing evidence highlights that robust peri‐implant soft tissue integration (STI) is essential for PID prevention by establishing a stable biological barrier that limits microbial invasion and mitigates chronic inflammation [3, 4]. However, inadequate peri‐implant STI compromises this protective barrier, facilitating microbial colonization and predisposing implants to persistent inflammatory damage, ultimately contributing to PID as a leading cause of implant failure [5, 6, 7]. Therefore, developing effective strategies to enhance peri‐implant STI remains a pressing clinical need.

Current strategies for enhancing peri‐implant STI mainly rely on implant surface modifications, such as topographical/physicochemical engineering, biofunctional coatings, antibacterial coatings, and immunomodulatory surfaces [8, 9, 10, 11, 12]. However, these approaches are frequently limited by their mono‐functionality, gradual bioactivity loss, and a lack of precise regulatory capacity. Following implant placement, the immature soft tissue interface remains vulnerable to early microbial colonization and inflammatory challenges during the healing process [6, 13]. Early bacterial accumulation can trigger excessive immune activation and oxidative stress, which may impair fibroblast function, extracellular matrix (ECM) remodeling, and vascular maturation, ultimately compromising the establishment of stable STI [5]. Impaired STI is not simply a problem of insufficient tissue attachment; rather, it is driven by an early pathogenic–immunological amplification loop [8, 14]. In this loop, *Staphylococcus aureus* (*S. aureus*), a key early colonizer with specific affinity toward titanium implant surfaces and biofilm‐forming capacity, initiates bacterial stress at the implant–soft tissue interface [15, 16]. This challenge, together with surgical trauma and implant‐induced oxidative stress (OS), drives macrophage dysfunction and excessive reactive oxygen species (ROS)‐associated inflammation [17]. Sustained inflammatory amplification then compromises fibroblast activity and angiogenesis and ultimately undermines peri‐implant STI [18, 19]. Thus, simultaneously targeting S. aureus and OS‐injured macrophages may provide a more precise strategy to disrupt this pathogenic‐immunological loop, restore peri‐implant microenvironmental homeostasis, and ultimately enhance STI.

Nanozymes, as artificial enzyme mimics, possess intrinsic catalytic activity to scavenge excessive ROS and restore redox homeostasis, while overcoming the instability and high cost associated with natural antioxidant enzymes [20, 21]. Recent studies have further demonstrated the potential of immunomodulatory nanozymes with targeting capabilities for regulating infection‐associated immune dysfunction and restoring host immune homeostasis [22]. However, precisely adapting these nanoengineered systems to complex peri‐implant microenvironments, where bacterial colonization, oxidative stress, and immune dysregulation coexist, remains challenging. Cell membrane‐coated biomimetic nanoparticles (CMBNs) have emerged as an appealing cell‐mimicking strategy, capable of targeted recognition and immunomodulation by preserving the complex receptor profile of the source cells [23, 24, 25]. Yet, their potential for enhancing peri‐implant soft tissue integration remains largely unexplored, especially in strategies designed to simultaneously address pathogen colonization and OS‐induced macrophage dysfunction. Upon bacterial challenge, macrophages undergo pathogen‐responsive activation, during which surface pattern recognition receptors (PRRs) and scavenger receptors are dynamically remodeled to recognize bacterial pathogen‐associated molecular patterns (PAMPs) and coordinate phagocytosis and intracellular sensing [26, 27]. We therefore hypothesized that membranes derived from *S. aureus*‐stimulated macrophages would be enriched with PRRs capable of binding *S. aureus*, while retaining intrinsic macrophage‐interactive features. Such CMBNs are expected to facilitate a dual‐targeting effect, encompassing both membrane‐mediated homotypic recognition of macrophages and specialized targeting of *S. aureus*.

**FIGURE 1:**
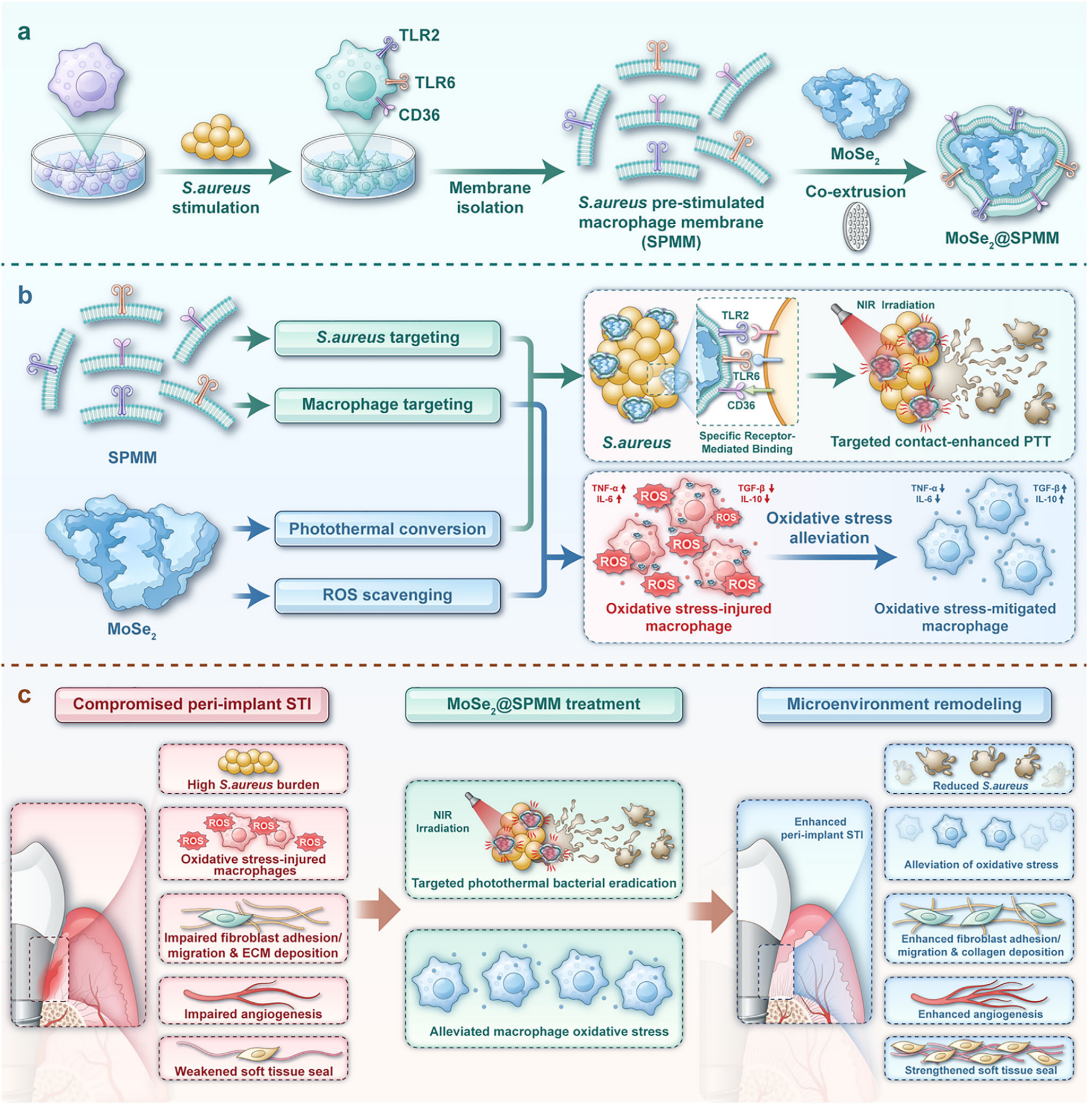
Schematic illustration of the construction and therapeutic mechanism of MoSe_2_@SPMM for enhancing peri‐implant STI. (a) *S. aureus*‐pre‐stimulated macrophage membranes (SPMM), enriched with pathogen‐recognition molecules including TLR2, TLR6, and CD36, were isolated and coated onto MoSe_2_ through co‐extrusion to construct MoSe_2_@SPMM. (b) MoSe_2_@SPMM integrates the dual‐targeting properties of SPMM with the multifunctional activities of MoSe_2_. SPMM‐mediated S. aureus targeting, attributed to pathogen‐recognition molecules such as TLR2, TLR6, and CD36, enhances bacterial binding and cooperates with the photothermal conversion capability of MoSe_2_ for targeted contact‐enhanced photothermal eradication. Meanwhile, SPMM‐mediated macrophage targeting through homotypic recognition facilitates macrophage interaction with MoSe_2_@SPMM, enabling ROS scavenging to alleviate OS‐induced macrophage dysfunction. (c) MoSe_2_@SPMM reduces bacterial burden and alleviates macrophage OS, thereby restoring fibroblast function and angiogenesis to enhance peri‐implant STI.

Herein, we developed a dual‐targeting biomimetic nanoplatform by camouflaging MoSe_2_ nanoflowers with *S. aureus*‐pre‐stimulated macrophage membranes (MoSe_2_@SPMM, Figure 1a).

Beyond their conventional biomedical applications, NIR‐responsive nanomaterials have attracted increasing attention for their potential in minimally invasive diagnosis and therapy owing to their deep tissue penetration and remote activation capability [28]. MoSe_2_ was selected as the functional nanozyme core owing to its intrinsic ROS‐scavenging multi‐enzyme mimetic activity, NIR‐responsive photothermal antibacterial capability, and favorable biocompatibility [29, 30, 31, 32, 33, 34]. Rather than simply integrating multiple functions, MoSe_2_@SPMM is rationally designed around two coordinated axes that jointly regulate the peri‐implant immune microenvironment: SPMM‐mediated S. aureus targeting, driven by enriched pathogen‐recognition molecules such as TLR2, TLR6, and CD36, couples with MoSe_2_‐mediated photothermal activity for contact‐enhanced bacterial eradication, while SPMM‐mediated macrophage targeting through homotypic recognition cooperates with MoSe_2_‐mediated ROS scavenging to alleviate OS‐induced injury (Figure 1b). In a rat oral implantation model, MoSe_2_@SPMM under localized 808 nm NIR irradiation reduced the peri‐implant bacterial burden by 91.4% and promoted macrophage reprogramming, thereby attenuating the pathogenic–immunological amplification loop and restoring a pro‐healing milieu. Consequently, angiogenesis increased by 2.3‐fold, collagen deposition by 67.1%, integrin expression by 61.4%, and peri‐implant connective tissue width by 1.7‐fold, contributing to enhanced peri‐implant STI (Figure 1c). Collectively, this dual‐targeting biomimetic nanozyme strategy provides a rational approach for regulating the peri‐implant immune microenvironment and promoting STI.

## 2 Results and Discussion

### 2.1 Proteomics Analysis of *S. aureus*‐Stimulated Macrophages

*S. aureus* exhibits a specific affinity toward titanium implant surfaces, enabling rapid colonization and initiating biofilm formation through enhanced interbacterial adhesion [15, 16, 35]. This process is considered a critical event in the disruption of peri‐implant STI and the onset of PID [14]. Further studies have demonstrated that *S. aureus* is highly representative during the early stage following implantation, and its colonization status possesses significant predictive value for subsequent microbial establishment  [36]. Moreover, among PID‐associated microorganisms, S. aureus displays a dominant distribution within peri‐implant niches, persisting across both healthy and inflamed conditions, thereby functioning as a core colonizer [37]. In addition, host immune responses against S. aureus are closely associated with implant prognosis, with antibody titer exhibiting significant predictive value for clinical outcomes [38, 39]. Notably, S. aureus has also been directly identified as a key pathogen in peri‐implantitis [40]. Based on these considerations, macrophages were pre‐stimulated with *S. aureus*, and the corresponding cell membranes were subsequently isolated. By leveraging the upregulated pathogen‐recognition‐related receptors on the stimulated macrophage membranes, this strategy aims to achieve enhanced and specific targeting toward S. aureus. Proteomic analysis was first performed on S. aureus‐stimulated macrophages to elucidate the underlying molecular response profiles.

**FIGURE 2:**
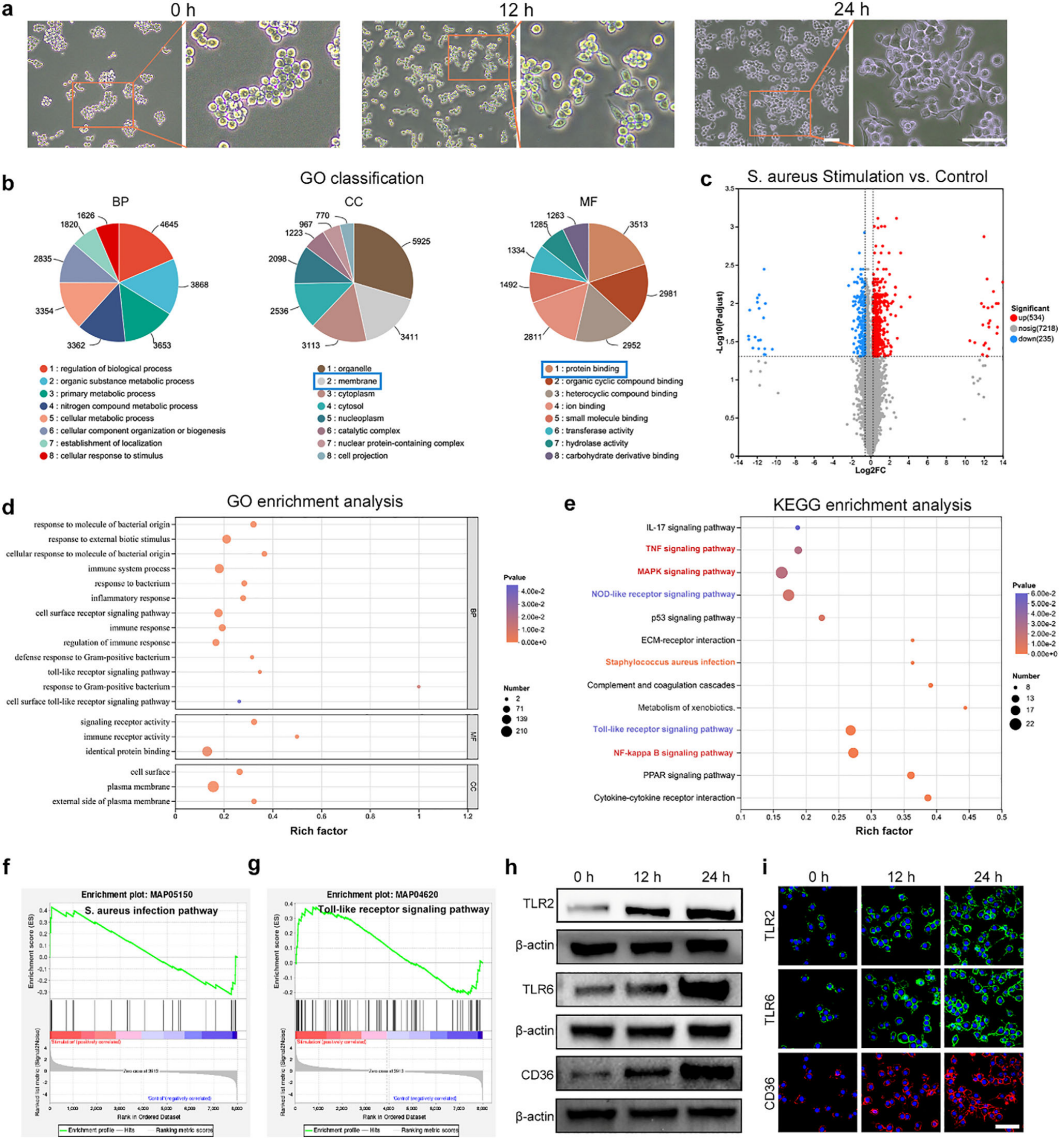
Proteomic analysis of macrophages following *S. aureus* stimulation and screening of membrane‐associated recognition receptors. (a) Morphological changes of RAW264.7 macrophages after *S. aureus* stimulation at different time points. Scale bar, 50 µm (both overview image and magnified image). (b) Gene Ontology classification of proteins identified by proteomic analysis, including biological process (BP), cellular component (CC), and molecular function (MF). (c) Volcano plot showing differentially expressed proteins between *S. aureus*‐stimulated macrophages and control cells. Red dots indicate significantly upregulated proteins, blue dots indicate significantly downregulated proteins, and gray dots represent proteins without significant change. (d) GO enrichment analysis of the differentially expressed proteins. (e) KEGG pathway enrichment analysis, revealing significant enrichment of several immune‐related signaling pathways. GSEA plots showing enrichment of the *S. aureus* infection pathway (f) and Toll‐like receptor signaling pathway (g) in *S. aureus*‐stimulated macrophages. (h) Western blot analysis of membrane‐associated recognition receptors (TLR2, TLR6, and CD36) in RAW264.7 at 0, 12, and 24 h after *S. aureus* stimulation. (i) Immunofluorescence staining showing the expression of TLR2, TLR6, and CD36 in macrophages after *S. aureus* stimulation. DAPI marks nuclei (blue); green indicates TLR2/TLR6, and red indicates CD36. Scale bar, 50 µm. Representative images in a, h, and i are from three biologically independent experiments.

To obtain *S. aureus* pre‐stimulated macrophages, RAW264.7 cells were co‐incubated with *S. aureus* for different durations. Stimulation induced time‐dependent morphological changes, including a spindle‐like phenotype and pseudopodia extension (Figure 2a). Cell viability initially increased but declined after prolonged stimulation, with a marked reduction after 24 h (Figure S1). Macrophages cultured for 24 h after stimulation were therefore selected for proteomic analysis. GO classification revealed that differentially expressed proteins were mainly enriched in metabolic and stimulus‐responsive processes, membrane‐ and organelle‐associated compartments, and protein binding activity (Figure 2b), suggesting substantial remodeling of membrane‐associated interaction proteins. Proteomic profiling identified 534 upregulated and 235 downregulated proteins after stimulation (Figure 2c). GO enrichment further showed that the upregulated proteins were predominantly involved in bacterial molecule recognition, PRR‐mediated pathogen sensing, Toll‐like receptor signaling, and immune‐inflammatory activation (Figure 2d). These proteins were enriched in receptor activity and protein binding and were mainly localized to the plasma membrane and its external side, indicating that *S. aureus* stimulation reshapes macrophage membrane receptor components. KEGG analysis showed marked enrichment of the *S. aureus* infection pathway, together with PRR‐related and inflammation‐associated pathways (Figure 2e). GSEA further confirmed positive enrichment of both the *S. aureus* infection and Toll‐like receptor signaling pathways in the stimulation group (Figure 2f,g), supporting coordinated activation of pathogen‐recognition programs in *S. aureus*‐stimulated macrophages.

Macrophage recognition and capture of bacteria largely depend on ligand–receptor interactions between membrane receptors and bacterial surface molecules [41]. Upon bacterial stimulation, macrophages undergo transcriptomic and proteomic reprogramming, leading to the upregulation of receptor molecules involved in bacterial recognition, binding, and immune activation [42]. Therefore, the enrichment of membrane‐associated receptor activity and Toll‐like receptor signaling observed in this study suggests that *S. aureus* stimulation may endow macrophage membranes with enhanced recognition potential toward *S. aureus*. Based on the marked enrichment of Toll‐like receptor signaling in the proteomic analysis, we next examined the expression of related TLRs on macrophage membranes in response to S. aureus stimulation. TLRs are key PRRs of the innate immune system, widely expressed on macrophages and other immune cells, where they recognize PAMPs derived from bacteria, viruses, and other pathogens, thereby initiating immune responses [42, 43]. RT–qPCR analysis showed that the transcript levels of *Tlr2*, *Tlr6*, and *Cd36* increased with prolonged *S. aureus* stimulation, whereas *Tlr4*, *Cd14*, and the virus‐associated receptors *Tlr3* and *Tlr7* remained unchanged (Figure S2). These findings suggest that *Tlr2*, *Tlr6*, and *Cd36* are preferentially responsive to *S. aureus* stimulation in macrophages. Consistent with the transcriptional results, Western blot and immunofluorescence analyses further confirmed that the protein levels of TLR2, TLR6, and CD36 were upregulated on the macrophage membrane during *S. aureus* stimulation (Figure 2h,i, Figures S3 and S4). Previous studies have exploited bacteria‐activated macrophage membrane coatings to improve bacterial binding or capture through the presence of Toll‐like receptors and other pathogen‐recognition molecules [44, 45]. However, most of these studies have relied on selected receptor validation rather than systematically comparing membrane‐associated protein changes before and after bacterial stimulation. In this study, proteomic profiling combined with transcriptional and protein‐level validation provides direct evidence that S. aureus stimulation remodels the macrophage membrane proteome and selectively enhances TLR2, TLR6, and CD36 expression. These findings provide a molecular basis for using *S. aureus* pre‐stimulated macrophage membranes as a biomimetic coating for constructing *S. aureus*‐targeted nanoplatforms.

### 2.2 Characterization of MoSe_2_@SPMM

**FIGURE 3:**
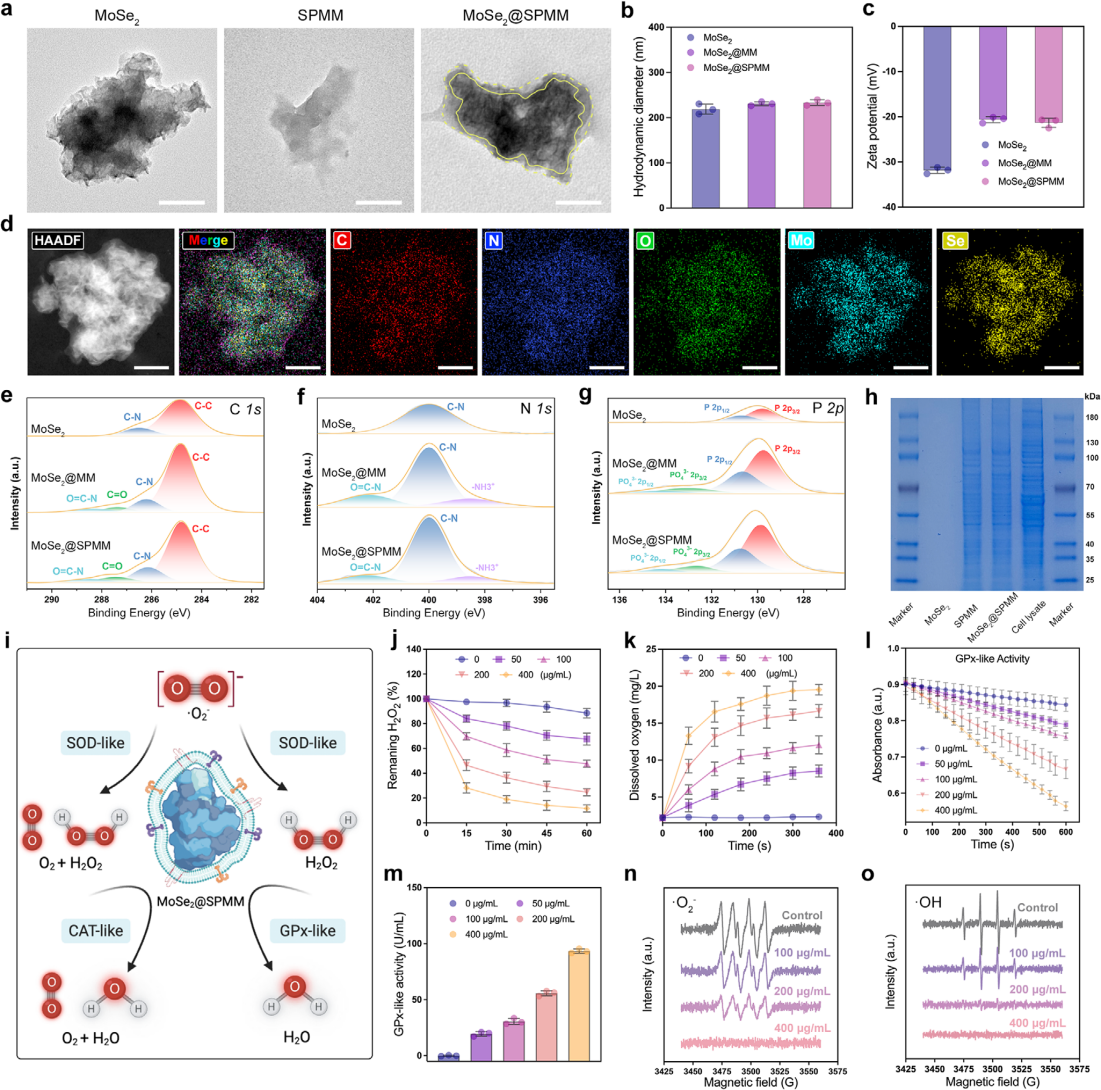
Characterization of MoSe_2_@SPMM with ROS‐scavenging capability. (a) Representative TEM images of MoSe_2_, *S. aureus*‐pre‐stimulated macrophage membranes (SPMM) and MoSe_2_@SPMM nanoparticles. Yellow dashed outlines indicate the membrane coating. Scale bar, 50 nm. Hydrodynamic diameter (b) and zeta potential (c) of MoSe_2_, MoSe_2_@MM and MoSe_2_@SPMM measured by DLS. Data are presented as mean ± s.d. (n = 3). (d) HAADF‐STEM image and elemental mapping of MoSe_2_@SPMM, showing the distribution of C, N, O, Mo and Se. Scale bar, 50 nm. High‐resolution XPS spectra of C 1s (e), N 1s (f) and P 2p (g) for MoSe_2_, MoSe_2_@MM and MoSe_2_@SPMM. (h) SDS–PAGE analysis of membrane proteins from MoSe_2_, SPMM, MoSe_2_@SPMM and macrophage lysates. (i) Schematic illustration of ROS scavenging by MoSe_2_@SPMM. Created with Biorender.com. (j) Time‐dependent decomposition of H_2_O_2_ by different concentrations of MoSe_2_@SPMM. Data are presented as mean ± s.d. (n = 3). (k) Dissolved oxygen generation during H_2_O_2_ decomposition in the presence of different concentrations of MoSe_2_@SPMM. Data are presented as mean ± s.d. (n = 3). (l) Time‐dependent GPx‐like catalytic activity of MoSe_2_@SPMM at different concentrations. Data are presented as mean ± s.d. (n = 3). (m) Quantitative analysis of the GPx‐like activity of MoSe_2_@SPMM at different concentrations. Data are presented as mean ± s.d. (n = 3). ESR spectra showing concentration‐dependent scavenging of •O_2_^−^ (n) and •OH (o) radicals by MoSe_2_@SPMM. Representative images in a, d and h are from three biologically independent experiments.

*S. aureus* pre‐stimulated macrophage membrane (SPMM) was first isolated via differential centrifugation and subsequently coated onto MoSe_2_ through ultrasonication and extrusion process to obtain MoSe_2_@SPMM. TEM revealed that pristine MoSe_2_ exhibited irregular, nanoflower‐like structures with sharp edges, whereas SPMM displayed a relatively uniform membranous morphology. After membrane coating, MoSe_2_@SPMM showed a smoother outline with a continuous shell surrounding the core (Figure 3a). MoSe_2_@MM, prepared using membranes from unstimulated macrophages, was included as a control for comparison. DLS analysis showed that MoSe_2_ had a mean hydrodynamic diameter of ∼218 nm, which slightly increased upon coating with either unstimulated macrophage membrane (MM) or SPMM (Figure 3b). Consistently, the zeta potential shifted from −31.9 mV for bare MoSe_2_ to −20.6 and −21.2 mV after coating with MM and SPMM, respectively (Figure 3c). The colloidal stability of MoSe_2_@SPMM was further evaluated in PBS and artificial saliva by DLS analysis. As shown in Figure S6, MoSe_2_@SPMM maintained a relatively stable hydrodynamic diameter over 7 days in both media, suggesting good dispersion stability under physiologically relevant conditions. Elemental mapping (merged image) showed that C, N, and O were relatively enriched at the particle periphery, whereas Mo and Se were predominantly localized in the core region (Figure 3d). Although signals of C, N, and O were detected across the entire particle, this is likely attributable to projection effects arising from electron beam penetration during TEM mapping. XPS analysis revealed the incorporation of membrane‐derived components, as evidenced by the emergence of O═C─N in the C 1s and N 1s spectra, and ─NH_3_^+^ peaks in theN 1s spectra of MoSe_2_@MM and MoSe_2_@SPMM (Figure 3e,f). The presence of PO_4_^3^^−^ signals in the P 2p spectrum further indicates phospholipid structures (Figure 3g). SDS‐PAGE confirmed that the protein profile of MoSe_2_@SPMM closely resembled that of SPMM but differed from that of whole cell lysates containing intracellular proteins (Figure 3h). Notably, Western blot analysis further showed that MoSe_2_@MM and MoSe_2_@SPMM retained membrane‐associated receptors involved in *S. aureus* recognition, including TLR2, TLR6, and CD36, with higher expression in MoSe_2_@SPMM than in MoSe_2_@MM (Figure S5). This result was consistent with their elevated expression levels in SPMM compared to MM (Figure 2h,i, Figures S3 and S4).

To evaluate the ROS scavenging and enzyme‐mimetic properties of MoSe_2_@SPMM, its catalytic decomposition of H_2_O_2_ was first assessed. As shown in Figure 3j, H_2_O_2_ decreased in a time and concentration‐dependent behavior, with ∼75% and ∼88% removed at 200 and 400 µg mL^−^^1^ within 60 min, respectively. Concurrently, dissolved oxygen increased during the reaction (Figure 3k), indicating O_2_ generation from H_2_O_2_ decomposition. Together with its stable performance over multiple cycles (Figure S7), these results support the catalase (CAT)‐like activity of MoSe_2_@SPMM. The glutathione peroxidase (GPx)‐like catalytic activity of MoSe_2_@SPMM was evaluated using a classical glutathione reductase (GR)‐coupled assay. As shown in Figure 3l, the absorbance decreased progressively over time in all groups, indicating ongoing catalytic reactions. Notably, the rate of absorbance decline increased with rising material concentration, exhibiting a typical concentration‐dependent behavior. Quantitative analysis further showed that the GPx‐like activity reached 55.8 and 93.4 U mL^−1^ at 200 and 400 µg mL^−^^1^, respectively (Figure 3m). The catalytic recyclability and chemical stability of MoSe_2_@SPMM were further evaluated. MoSe_2_@SPMM retained substantial GPx‐like activity after three consecutive catalytic cycles, with only a slight decrease in relative activity (Figure S8a). XPS analysis of Se 3d and Mo 3d showed no obvious changes before and after catalytic cycling (Figure S8b,c), suggesting that the GPx‐like activity mainly arises from recyclable catalytic processes rather than rapid material consumption.

Electron spin resonance (ESR) spectroscopy was used to assess the ROS scavenging activity of MoSe_2_@SPMM using 5,5‐dimethyl‐1‐pyrroline N‐oxide (DMPO) as a spin trap. The characteristic DMPO/·O_2_^−^ signal (1:1:1:1) was progressively suppressed with increasing MoSe_2_@SPMM concentration and nearly vanished at higher doses (Figure 3n), indicating efficient ·O_2_^−^ scavenging. Similarly, the DMPO/·OH signal (1:2:2:1) decreased in a concentration‐dependent manner and was almost completely quenched at 200 µg mL^−^^1^ (Figure 3o). In agreement with these results, the nitroblue tetrazolium (NBT) assay further confirmed the strong ·O_2_^−^ scavenging capacity (Figure S9a), suggesting superoxide dismutase (SOD)‐like activity for ROS scavenging. To exclude the effect of membrane coating, the ROS‐scavenging performance of MoSe_2_, MoSe_2_@MM, and MoSe_2_@SPMM was compared (Figure S9a–c). All samples exhibited pronounced scavenging activity toward these radicals, with no significant differences observed among the groups, indicating that coating with either MM or SPMM does not impair the intrinsic antioxidative activity of MoSe_2_. These extracellular catalytic activities provide the biochemical basis for the antioxidative function of MoSe_2_@SPMM. Upon macrophage‐targeted internalization, MoSe_2_@SPMM may further regulate the intracellular oxidative microenvironment by reducing ROS accumulation, which was subsequently confirmed by cellular ROS and mitochondrial functional analyses.

Additionally, MoSe_2_@SPMM exhibited a pronounced photothermal response under 808 nm NIR irradiation. The temperature increased over time with rising laser power density (0–1.0 W cm^−^^2^), indicating power‐dependent heating behavior (Figure S10a). A similar concentration‐dependent trend was observed, with higher temperatures achieved at increased material concentrations (50–500 µg mL^−^^1^) (Figure S10b). MoSe_2_@SPMM also showed stable and repeatable heating profiles over multiple on/off irradiation cycles, without significant attenuation (Figure S10c).

### 2.3 Biosafety Evaluation

As shown in Figure S11a–c, MoSe_2_@SPMM exhibited good cytocompatibility within the concentration range of 0–200 µg mL^−1^, with no significant reduction in cell viability observed in different cell types. In contrast, a marked decrease in cell viability was observed at 400 µg mL^−1^ in all cell types, indicating potential cytotoxicity at this higher concentration. Live/dead staining was further performed to evaluate cell viability at 200 µg mL^−1^ (Figure S11d). Predominantly green fluorescence with minimal red signals was observed in different cell types, with no apparent differences between the control and MoSe_2_@SPMM groups. These findings are consistent with the Cell Counting Kit‐8 (CCK‐8) results, further confirming the good cytocompatibility of MoSe_2_@SPMM at this concentration. Furthermore, the effect of NIR irradiation on macrophage viability at this concentration was evaluated over 1, 3, and 5 days (Figure S11e). A slight reduction in cell viability was observed in the NIR+ group at day 1; however, cell viability gradually recovered over time, and no significant differences were observed among the groups at days 3 and 5. Based on these results, 200 µg mL^−1^ was selected as a safe concentration for subsequent experiments. The hemocompatibility of MoSe_2_@SPMM was evaluated by a hemolysis assay. As shown in Figure S11f,g, obvious hemolysis was observed in the H_2_O group, whereas negligible hemolysis was detected in the saline group and all MoSe_2_@SPMM‐treated groups across the tested concentration range, indicating good hemocompatibility of MoSe_2_@SPMM. At the end of the in vivo study, major organs were harvested for H&E staining, and blood samples were collected for routine hematological and biochemical analyses. Histological examination revealed no obvious tissue damage, necrosis, or inflammatory infiltration in the major organs following MoSe_2_@SPMM treatment (Figure S11h). In addition, no significant abnormalities were observed in routine blood parameters or plasma biochemical indicators, suggesting that MoSe_2_@SPMM does not induce detectable adverse effects on the hematological system or liver and kidney function (Figure S11i).

### 2.4 Targeting‐Enhanced Photothermal Antibacterial Activity of MoSe_2_@SPMM Against *S. aureus* In Vitro

**FIGURE 4:**
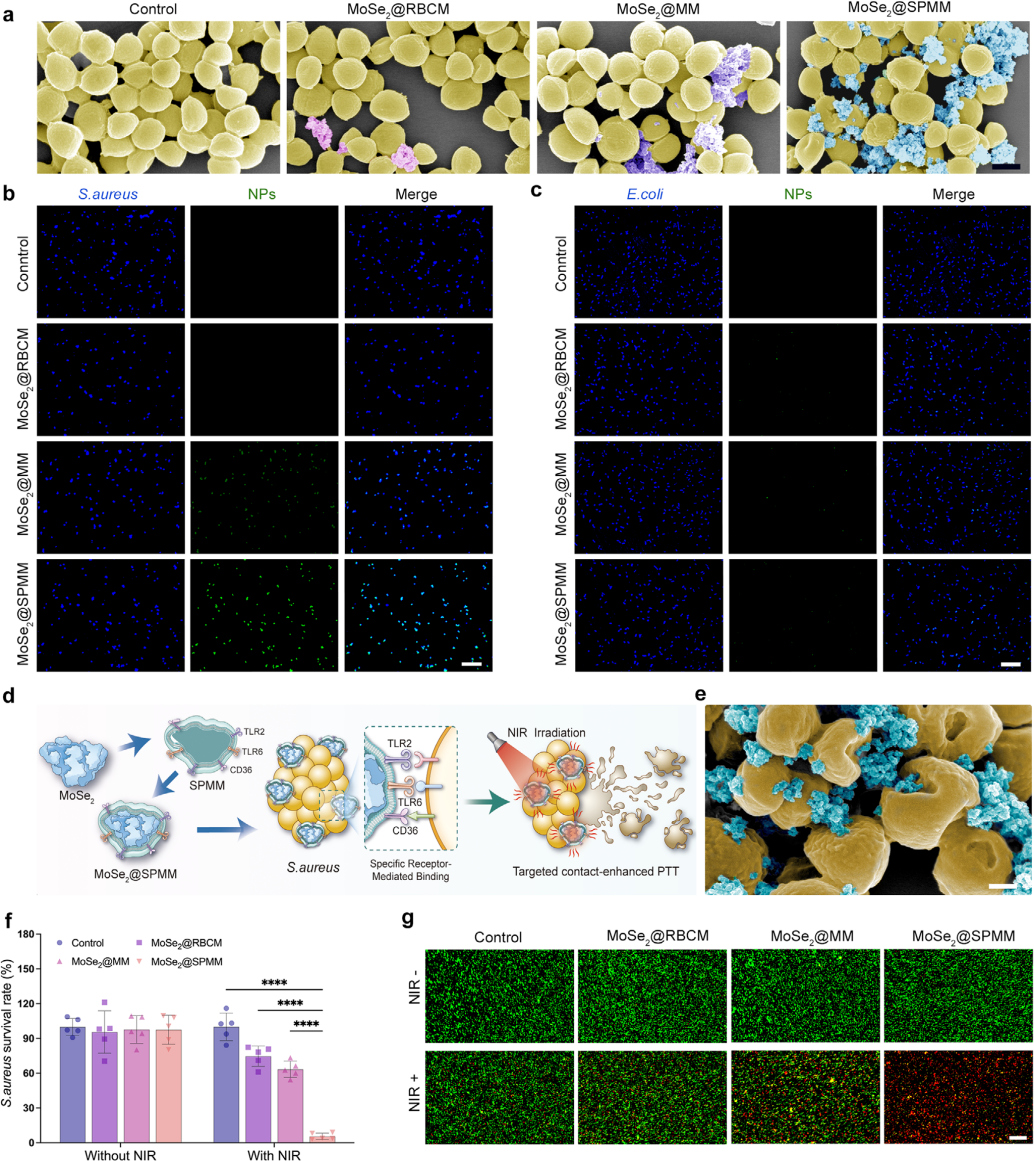
*S. aureus* targeting potentiates the photothermal antibacterial activity of MoSe_2_@SPMM. (a) SEM images showing the interaction between different nanoparticles, including MoSe_2_@RBCM, MoSe_2_@MM and MoSe_2_@SPMM, and S. aureus. Scale bar, 500 nm. Confocal fluorescence images showing nanoparticle binding to S. aureus (b) and E. coli (c). Bacteria were stained with Hoechst 33342 (blue), and nanoparticles were labelled with DiO (green). Scale bars, 20 µm. (d) Schematic illustration of targeted contact‐enhanced photothermal killing of S. aureus mediated by MoSe_2_@SPMM. (e) Representative SEM image showing the interaction between MoSe_2_@SPMM and S. aureus. Scale bar, 200 nm. (f) Survival rate of S. aureus after various treatments, analyzed by plating bacteria on lysogeny broth agar plates. Data are presented as mean ± s.d. (n = 5 biological replicates). Statistical significance was calculated using one‐way ANOVA with Tukey's post hoc test. (g) Live/dead fluorescence staining of S. aureus after different treatments. Live bacteria are stained green and dead bacteria are stained red. Scale bar, 20 µm. Images in a, b, c, and e are representative of three biologically independent experiments; g is representative of five. NPs, nanoparticles. ^****^, *p* < 0.0001.

We first evaluated the in vitro targeting behavior of MoSe_2_@SPMM toward *S. aureus*. In addition to MoSe_2_@MM, red blood cell membrane–coated MoSe_2_ (MoSe_2_@RBCM) was introduced as a control, as it is neither macrophage‐derived nor enriched in receptors such as TLR2, TLR6, and CD36 (Figure S12). MoSe_2_ coated with RBCM, MM, or SPMM at identical concentrations were incubated with *S. aureus* and examined by scanning electron microscopy (SEM) (Figure 4a). MoSe_2_@RBCM showed little to no association with *S. aureus*, whereas MoSe_2_@MM exhibited a moderate level of attachment. By contrast, MoSe_2_@SPMM displayed extensive binding to *S. aureus* under the same conditions. These observations were corroborated by confocal imaging. As shown in Figure 4b, DiO‐labeled MoSe_2_@RBCM showed almost no colocalization with Hoechst 33342‐stained *S. aureus* after incubation. MoSe_2_@MM displayed a detectable but limited degree of interaction, whereas MoSe_2_@SPMM produced a strong colocalization signal along the *S. aureus*, indicating enhanced targeting affinity. To further exclude nonspecific effects associated with lipid coating, 1,2‐dioleoyl‐sn‐glycero‐3‐phosphocholine (DOPC)‐coated MoSe_2_ (MoSe_2_@DOPC) was introduced as a synthetic lipid‐coated neutral control. Compared with MoSe_2_@DOPC, MoSe_2_@SPMM showed markedly stronger association with S. aureus (Figure S13), further supporting that SPMM‐enriched pathogen‐recognition molecules contribute to its bacterial targeting capability. To examine specificity, the same experiment was then performed with *E. coli* (Figure 4c). In this case, none of the groups showed appreciable colocalization, and the differences between them were minimal, suggesting that the targeting behavior exhibits a certain degree of bacterial selectivity. These results provide strong evidence for the *S. aureus*–targeting capability of MoSe_2_@SPMM. This *S. aureus* targeting may be associated with the retention and enrichment of pathogen‐recognition molecules on SPMM after *S. aureus* pre‐stimulation. Based on proteomic screening, TLR2, TLR6, and CD36 were identified as candidate membrane receptors related to *S. aureus* recognition. TLR2 can form a heterodimer with TLR6 to participate in the recognition of *S. aureus*‐associated PAMPs, whereas CD36 may act as a scavenger receptor and TLR2/TLR6‐associated coreceptor to facilitate the recognition, phagocytosis, and intracellular delivery [46, 47, 48]. Therefore, the simultaneous enrichment of TLR2, TLR6, and CD36 on SPMM may provide a molecular basis for the enhanced binding of MoSe_2_@SPMM to *S. aureus*. Notably, effective targeting toward a specific bacterium is usually not determined by a single receptor but is more likely to rely on the coordinated action of multiple recognition molecules. This also highlights a key advantage of CMBNs: they inherit the natural multi‐receptor recognition features of source cells, thereby enabling complex biological functions that are difficult to reproduce using single‐ligand modification strategies.

Building on its targeting capability, we next examined whether this interaction translates into improved photothermal antibacterial performance. As illustrated in Figure 4d, MoSe_2_@SPMM retains receptor proteins from SPMM, including TLR2, TLR6, and CD36, enabling receptor‐mediated binding to the bacterial surface. Upon NIR irradiation, the locally enriched MoSe_2_ nanoparticles generate a more effective photothermal response, resulting in a targeted contact‐enhanced antibacterial effect. SEM images showed marked bacterial damage after MoSe_2_@SPMM treatment under NIR irradiation, including surface roughening, membrane perforation, and severe deformation and collapse (Figure 4e). Antibacterial activity was assessed by colony formation assays (Figure 4f). Without NIR irradiation, colony numbers were comparable across groups, indicating negligible antibacterial effects. Upon NIR exposure, MoSe_2_@SPMM showed markedly enhanced bactericidal activity, outperforming MoSe_2_@MM and MoSe_2_@RBCM, which displayed moderate and minimal effects, respectively. Live/Dead staining provided a more direct visualization of antibacterial efficacy (Figure 4g). Under NIR, viable bacteria dominated in the control and MoSe_2_@RBCM groups, while MoSe_2_@MM showed mixed populations and MoSe_2_@SPMM was largely composed of dead bacteria. We next extended this evaluation from planktonic bacteria to biofilms, which are more resistant and clinically relevant in PID. Both crystal violet staining (Figure S14a) and biomass quantification (Figure S14b) confirmed that MoSe_2_@SPMM effectively suppressed biofilm formation under NIR irradiation, whereas MoSe_2_@MM and MoSe_2_@RBCM exhibited limited inhibition. Additionally, 3D reconstruction and cross‐sectional views revealed pronounced biofilm disruption in the MoSe_2_@SPMM group under NIR, characterized by reduced thickness, a discontinuous structure, and strong red signals across the biofilm depth, indicating killing throughout the structure (Figure S14c). In contrast, viable bacteria persisted in the other groups, particularly in deeper layers. These results suggest that SPMM endows MoSe_2_ with a biologically recognizable interface that promotes receptor‐mediated bacterial surface accumulation and enhances nanoparticle–bacteria contact. Importantly, SPMM‐mediated bacterial targeting primarily facilitates the selective localization of MoSe_2_@SPMM to S. aureus, whereas the photothermal conversion capability of MoSe_2_ provides the major bactericidal effect upon NIR irradiation, leading to enhanced contact‐dependent photothermal killing and effective biofilm disruption. Therefore, the superior antibacterial performance of MoSe_2_@SPMM results from the synergistic integration of targeting‐enhanced bacterial localization and photothermal‐mediated bacterial elimination.

### 2.5 MoSe_2_@SPMM Restores Macrophage Function Through Homotypic Targeting and ROS Scavenging

**FIGURE 5:**
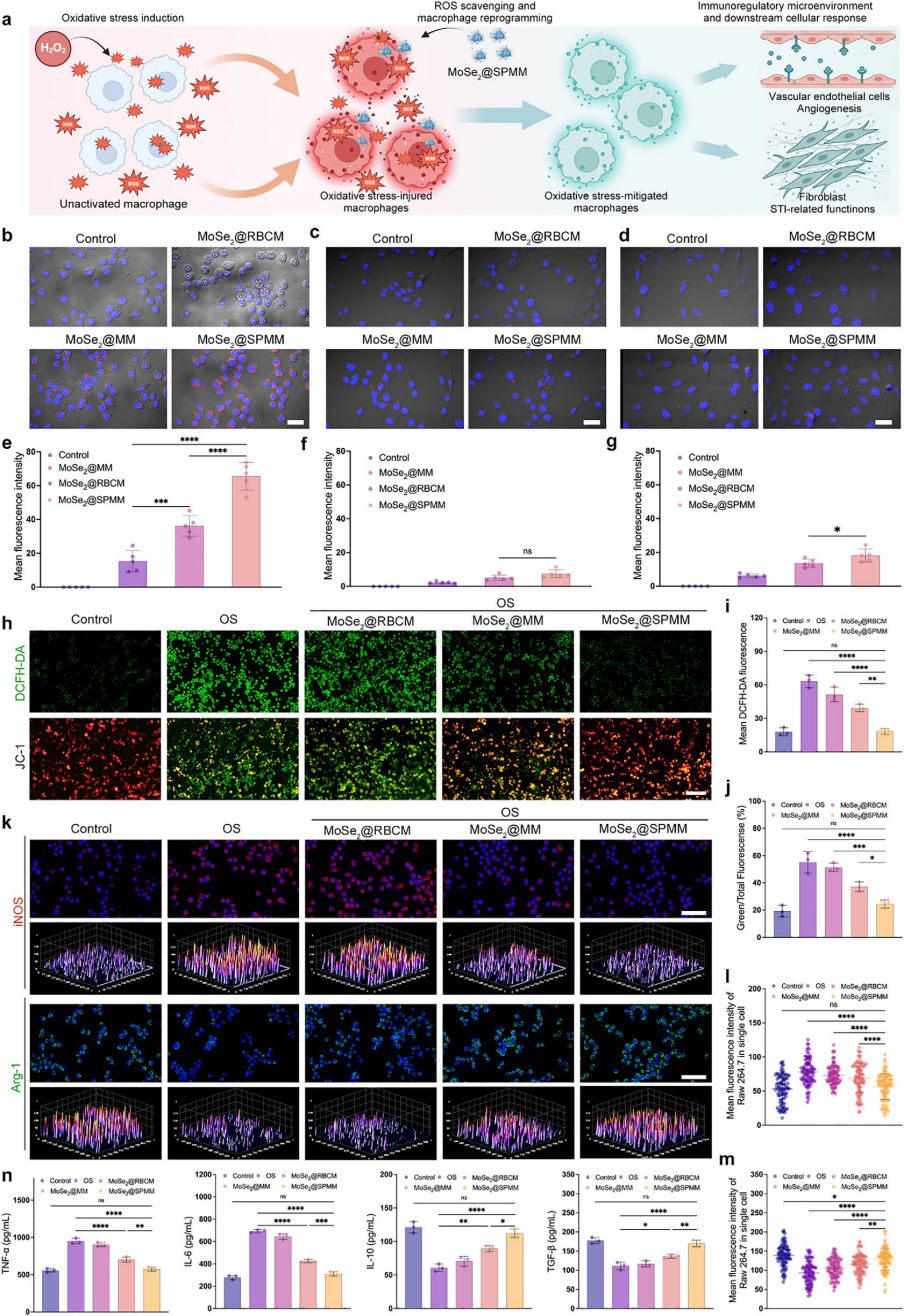
Macrophage‐targeting and ROS scavenging by MoSe_2_@SPMM. (a) Schematic illustration of macrophage‐homotypic targeting and ROS‐scavenging‐mediated functional recovery by MoSe_2_@SPMM. Created with Biorender.com. b–d, Confocal fluorescence images showing the internalization of MoSe_2_@RBCM, MoSe_2_@MM and MoSe_2_@SPMM by macrophages (b), fibroblasts (c) and HUVECs (d). Cell nuclei were stained with DAPI (blue), and nanoparticles were labeled with Dil (red). Scale bars, 20 µm. Quantification of nanoparticle uptake based on mean fluorescence intensity in macrophages (e), fibroblasts (f), and HUVECs (g). Data are presented as mean ± s.d. (n = 5 biological replicates). (h) Representative images of intracellular ROS levels detected by DCFH‐DA staining and mitochondrial membrane potential assessed by JC‐1 staining in RAW264.7 macrophages under different treatments. DCFH‐DA fluorescence is shown in green, with stronger fluorescence indicating higher intracellular ROS levels; red JC‐1 aggregates indicate preserved mitochondrial membrane potential, whereas green JC‐1 monomers indicate mitochondrial depolarization. Scale bar, 100 µm. (i) Quantitative analysis of intracellular ROS levels based on DCFH‐DA fluorescence intensity. Data are presented as mean ± s.d. (n = 3 biological replicates). (j) Quantification of mitochondrial membrane potential based on the JC‐1 green/total fluorescence ratio. Data are presented as mean ± s.d. (n = 3 biological replicates). Immunofluorescence staining of iNOS and Arg‐1 in RAW264.7 macrophages under OS conditions, with corresponding three‐dimensional surface plots (k), and quantification of iNOS (l) and Arg‐1 (m) mean fluorescence intensity. Scale bar, 50 µm. Each dot represents one cell. Twenty‐five cells were analysed per group in each of four independent experiements (100 cells per group in total). Data are presented as mean ± s.d. (n) ELISA analysis of TNF‐α, IL‐6, IL‐10 and TGF‐β secretion from macrophages under different treatment conditions. Data are presented as mean ± s.d. (n = 3 biological replicates). Statistical significance was calculated via one‐way ANOVA with Tukey's post hoc test. OS, oxidative stress. ns, non‐significant. ^*^, *p* < 0.05. ^**^, *p* < 0.01. ^***^, *p* < 0.001. ^****^, *p* < 0.0001.

As conceptually illustrated in Figure 5a, under OS, excessive ROS accumulation disrupts macrophage redox homeostasis and promotes a pro‐inflammatory state, which further amplifies local inflammation and impairs downstream endothelial and fibroblast functions. Through SPMM‐mediated homotypic targeting, MoSe_2_@SPMM preferentially accumulates in macrophages, where its intrinsic ROS‐scavenging capability contributes to the alleviation of intracellular oxidative stress. This regulation contributes to a pro‐healing immune microenvironment that supports angiogenesis and fibroblast‐mediated matrix remodeling. We first evaluated whether MoSe_2_@SPMM could preferentially target macrophages under OS. Compared with MoSe_2_@RBCM and MoSe_2_@MM, MoSe_2_@SPMM showed substantially stronger fluorescence signals in macrophages, indicating enhanced macrophage‐homotypic internalization (Figure 5b). In contrast, relatively weak fluorescence signals were observed in fibroblasts (Figure 5c) and vascular endothelial cells (Figure 5d) across all groups, indicating that MoSe_2_@SPMM exhibited selective macrophage targeting while maintaining low nonspecific uptake in other cell types. Quantitative analysis confirmed that MoSe_2_@SPMM accumulated preferentially in macrophages (Figure 5e) rather than in fibroblasts (Figure 5f) or endothelial cells (Figure 5g). Considering that RBCM may contain CD47 and thereby suppress macrophage phagocytosis, DOPC‐coated MoSe_2_ (MoSe_2_@DOPC) was further introduced as a synthetic lipid‐coated neutral control. MoSe_2_@SPMM exhibited significantly higher macrophage internalization than MoSe_2_@DOPC (Figure S15), supporting the role of SPMM‐mediated macrophage homotypic recognition in macrophage targeting. These results demonstrate that the SPMM coating enables MoSe_2_ to selectively target OS‐injured macrophages. The limited uptake of MoSe_2_@RBCM is likely associated with CD47 on RBCM surface, which acts as a “don't eat me” signal to suppress macrophage phagocytosis [49, 50]. In contrast, macrophage‐derived membranes may preserve homotypic recognition‐associated proteins, thereby enhancing macrophage recognition and internalization of MoSe_2_@MM. Notably, the further increased uptake of MoSe_2_@SPMM suggests that *S. aureus* stimulation may remodel the macrophage membrane proteome and strengthen SPMM‐mediated recognition and internalization. Consistently, bacterial stimulation has been reported to enrich pattern‐recognition and scavenger receptors, which can facilitate receptor‐mediated CMBNs internalization [51, 52, 53]. Thus, the enhanced macrophage uptake of MoSe_2_@SPMM is likely associated with stimulation‐induced membrane proteome remodeling rather than membrane coating alone. Macrophage membrane coating confers context‐dependent uptake behavior. Previous studies have reported that macrophage membranes can reduce nonspecific phagocytic clearance through immune‐evasive effects [54, 55]. On the other hand, consistent with our results, studies have shown that macrophage membrane‐coated nanoparticles can exhibit homotypic macrophage targeting and enhanced cellular internalization [56, 57]. Therefore, macrophage membranes should not be regarded simply as “stealth” shells; rather, their biological behavior is determined by the dynamic balance between anti‐phagocytic signals and active membrane protein‐mediated recognition. Previous studies have shown that enzymatic digestion of membrane proteins significantly reduces the uptake of macrophage membrane‐coated nanoparticles, further supporting the critical role of membrane proteins in homotypic targeting and cellular internalization [58]. Taken together, MoSe_2_@SPMM preferentially targets and is internalized by OS‐injured macrophages, providing a cellular basis for macrophage‐directed redox regulation.

We next examined whether this targeted internalization could alleviate OS‐induced macrophage injury. Intracellular ROS levels and mitochondrial membrane potential (ΔΨm) were assessed using DCFH‐DA and JC‐1 staining, respectively (Figure 5h–j). Compared with the control group, H_2_O_2_ treatment led to a marked increase in DCFH‐DA fluorescence, indicating substantial ROS accumulation (Figure 5h). In parallel, JC‐1 staining revealed a significant shift from red to green fluorescence (Figure 5h). Given that JC‐1 forms red‐emissive aggregates at high ΔΨm and transitions to green‐emissive monomers upon the decrease of ΔΨm [59], these results indicate mitochondrial depolarization and OS–induced mitochondrial impairment. Following material treatment, all groups exhibited partial recovery, with reduced ROS levels to varying extents. Notably, MoSe_2_@SPMM showed the weakest DCFH‐DA signal, indicating the most effective ROS scavenging (Figure 5h,i). Correspondingly, JC‐1 staining in this group showed a clear restoration of red fluorescence and a reduction in green signals, suggesting recovery of ΔΨm (Figure 5h–j). Although MoSe_2_@MM and MoSe_2_@RBCM also showed certain improvements, their effects were less pronounced. Overall, MoSe_2_@SPMM effectively reduces intracellular ROS and alleviates OS, thereby mitigating mitochondrial dysfunction and maintaining cellular homeostasis. Building on these findings, we next examined whether alleviating OS could translate into macrophage reprogramming. To this end, the expression of the pro‐inflammatory marker iNOS and the repair‐associated marker Arg‐1 was evaluated (Figure 5k–m). Under OS, iNOS expression was markedly upregulated, indicating a shift toward a pro‐inflammatory state. Treatment with membrane‐coated MoSe_2_ nanoparticles reduced iNOS levels to varying extents, with MoSe_2_@SPMM showing the most pronounced suppression, followed by MoSe_2_@MM, while MoSe_2_@RBCM had only a limited effect (Figure 5k,l). In contrast, Arg‐1 expression was reduced under OS, suggesting impaired repair‐associated function. Following treatment, Arg‐1 levels were partially restored across all groups, with the greatest increase observed in the MoSe_2_@SPMM group (Figure 5k–m). This effect is likely driven by its ROS‐scavenging capacity, which alleviates OS and restores mitochondrial function. We next assessed macrophage secretory profiles. Enzyme‐linked immunosorbent assay (ELISA) showed that OS increased TNF‐α and IL‐6 while reducing IL‐10 and TGF‐β, indicating a pro‐inflammatory–dominant profile (Figure 5n). Following treatment, TNF‐α levels decreased across all groups, most notably in the MoSe_2_@SPMM group, followed by MoSe_2_@MM, while MoSe_2_@RBCM showed only a limited effect. In contrast, IL‐10 secretion was restored to varying degrees, with the greatest increase in the MoSe_2_@SPMM group. Overall, MoSe_2_@SPMM modulates intracellular ROS levels and preserves mitochondrial function, thereby alleviating OS–induced macrophage dysfunction. This drives a shift from a pro‐inflammatory state toward a repair‐associated phenotype and reshapes the macrophage secretory profile, ultimately establishing a pro‐regenerative immune microenvironment.

### 2.6 MoSe_2_@SPMM‐Regulated Macrophage Microenvironment Promotes Vascular Endothelial Functional Recovery

**FIGURE 6:**
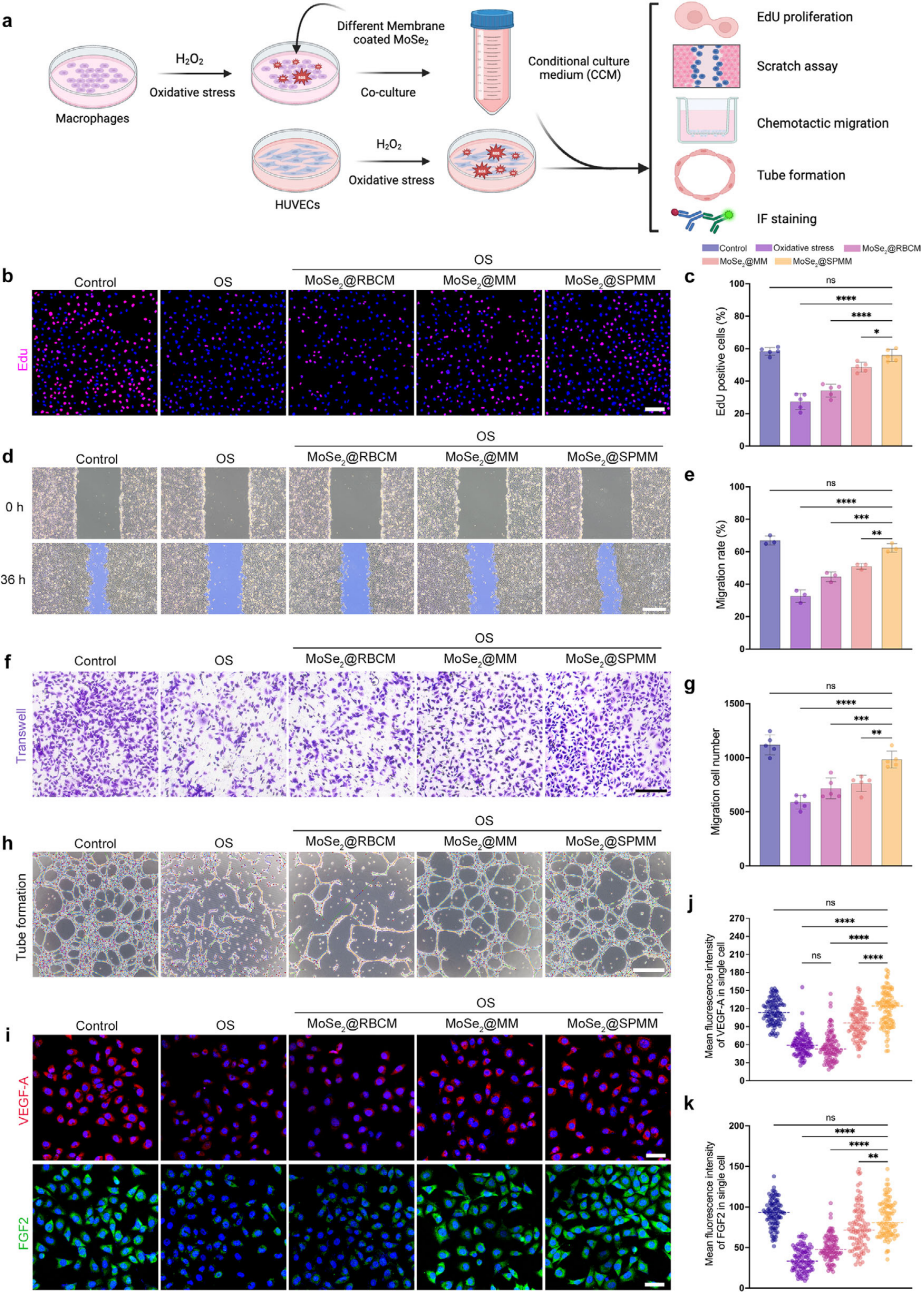
MoSe_2_@SPMM‐regulated macrophage microenvironment improves vascular endothelial cell function under OS. (a) Experimental workflow. Macrophages were exposed to H_2_O_2_ to induce OS, treated with different MoSe_2_‐based nanoparticles, and the collected CCM was applied to OS‐injured HUVECs to assess proliferation, migration, tube formation, and angiogenesis‐related protein expression. Created with Biorender.com. (b) Representative EdU staining images showing the proliferation of HUVECs cultured with CCM from different treatment groups. EdU‐positive cells are shown in magenta and nuclei in blue. Scale bar, 200 µm. (c) Quantitative analysis of the percentage of EdU‐positive HUVECs under different treatment conditions. Data are presented as mean ± s.d. (n = 5 biological replicates). (d) Representative scratch wound‐healing images at 0 and 36 h, used to assess the migratory capacity of HUVECs cultured with CCM from different groups. The blue area indicates the wound region. Scale bar, 50 µm. (e) Quantitative analysis of HUVEC migration rates in the scratch assay. Data are presented as mean ± s.d. (n = 3 biological replicates). (f) Representative images of Transwell migration assays showing the migration of HUVECs cultured with CCM from different treatment groups. Migrated cells were stained with crystal violet. Scale bar, 200 µm. (g) Quantitative analysis of the number of migrated HUVECs in the Transwell assay. Data are presented as mean ± s.d. (n = 5 biological replicates). (h) Representative images of tube formation by HUVECs cultured with CCM from different treatment groups. Scale bar, 50 µm. (i) Immunofluorescence staining of the angiogenesis‐related factors VEGF‐A and FGF2 in HUVECs cultured with CCM from different groups. Scale bar, 50 µm. Quantification of VEGF‐A (j) and FGF2 (k) fluorescence intensity in HUVECs. Data are presented as mean ± s.d.Each dot represents one cell. Twenty‐five cells were analysed per group in each of four independent experiments (100 cells per group in total) Representative images in **d** and **i** are from three biologically independent experiments; b, f, and h are from five. Statistical significance was calculated via one‐way ANOVA with Tukey's post hoc test. OS, oxidative stress. ns, non‐significant. ^*^, *p* < 0.05. ^**^, *p* < 0.01. ^***^, *p* < 0.001. ^****^, *p* < 0.0001.

Having established that MoSe_2_@SPMM alleviates OS and reprograms macrophage function to generate a pro‐regenerative immune microenvironment, we next examined whether this microenvironment could modulate downstream vascular endothelial cell responses. Vascular endothelial cells are critical regulators of peri‐implant STI, as angiogenesis and vascular maturation provide essential support for tissue maturation and remodeling during soft tissue healing. Therefore, endothelial functional recovery was evaluated as an important indicator of the regenerative capacity of the macrophage‐regulated microenvironment [4]. As illustrated in Figure 6a, macrophages were first subjected to H_2_O_2_‐induced OS and treated with differently membrane‐coated MoSe_2_, after which the conditioned culture medium (CCM) was collected and applied to endothelial cells under OS. Proliferation, migration, and angiogenesis were then systematically evaluated using 5‐ethynyl‐2′‐deoxyuridine (EdU) incorporation, scratch wound healing, transwell migration, and tube formation assays. As shown in Figure 6b,c, OS markedly impaired the proliferative capacity of vascular endothelial cells. Treatment with CCM derived from MoSe_2_@SPMM‐treated macrophages effectively restored proliferation, as evidenced by an increased proportion of EdU‐positive cells. MoSe_2_@MM‐CCM produced a moderate improvement, whereas MoSe_2_@RBCM‐CCM showed minimal effect. Consistent with the inhibition of proliferation, cell migration was also significantly compromised under OS (Figure 6d,e). In the scratch assay, wound closure was clearly delayed, while treatment with MoSe_2_@SPMM‐CCM accelerated closure, indicating a recovery of migratory capacity. A similar trend was observed in the transwell assay, where the number of migrated cells was markedly increased in the MoSe_2_@SPMM‐CCM group, with only partial improvement in the MoSe_2_@MM‐CCM group and limited effect in the MoSe_2_@RBCM‐CCM group (Figure 6f,g). Moreover, the functional changes in vascular endothelial cells were notably manifested in their angiogenic capacity (Figure 6h). Under OS, cells failed to form an intact network structure, whereas treatment with MoSe_2_@SPMM‐CCM resulted in a continuous and well‐organized tubular network with markedly increased branching. Quantitative analysis (Figure S16a–c) also confirmed significantly higher numbers of nodes, junctions, and meshes in the MoSe_2_@SPMM‐CCM group compared with other treatments. The functional recovery was underpinned by a significant enrichment in vascular endothelial growth factor A (VEGF‐A) and fibroblast growth factor 2 (FGF2), which are indispensable for orchestrating the angiogenic process (Figure 6i–k). OS reduced the expression of VEGF‐A and FGF2, while both were effectively restored following treatment with MoSe_2_@SPMM‐CCM. Taken together, MoSe_2_@SPMM alleviates OS–induced damage in macrophages and modulates their functional state, thereby establishing a pro‐healing immune microenvironment. This, in turn, restores endothelial proliferation, migration, and angiogenic capacity under OS, indicating its potential to promote vascularized tissue repair.

### 2.7 MoSe_2_@SPMM‐Regulated Macrophage Microenvironment Promotes Fibroblast Functional Recovery

**FIGURE 7:**
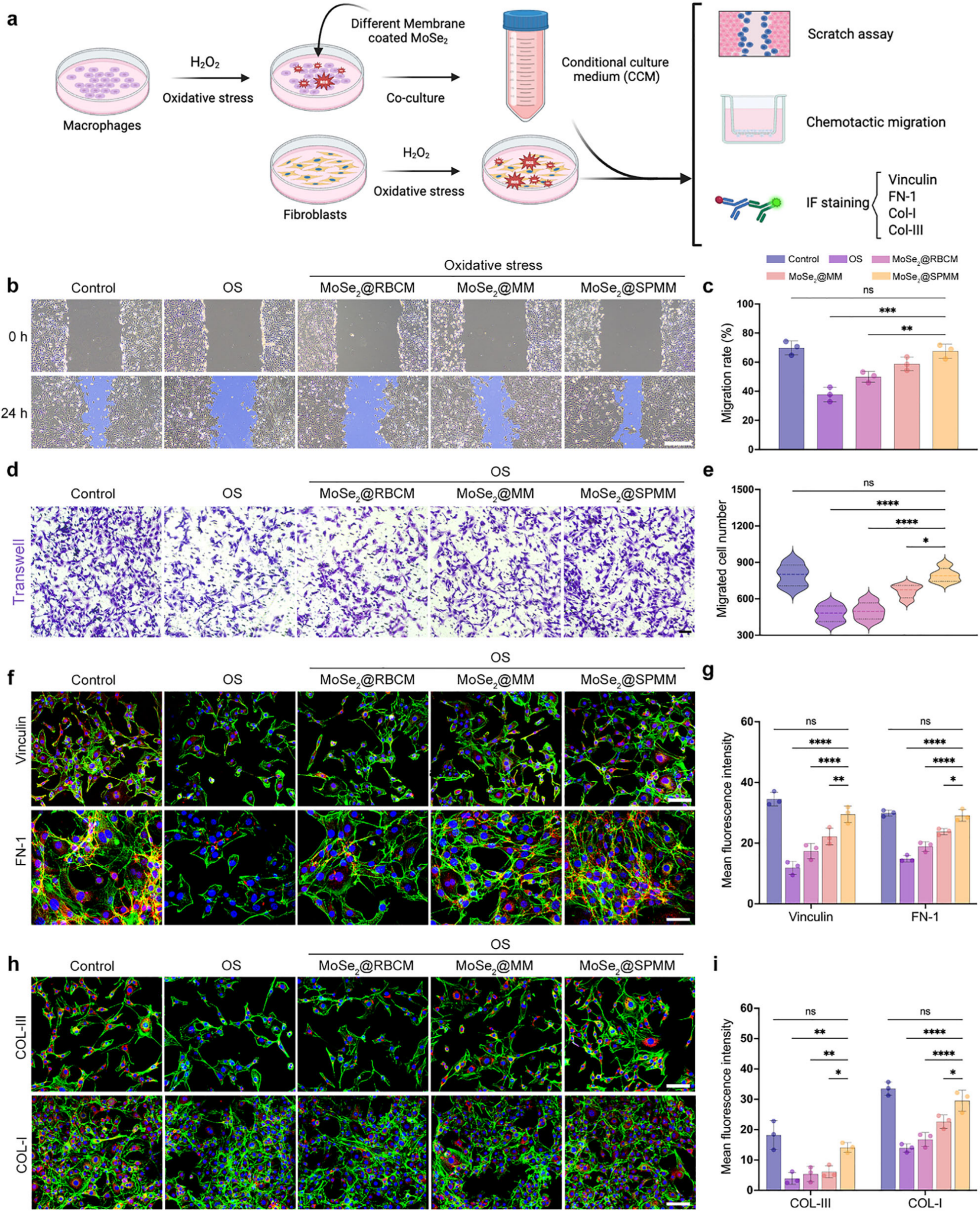
MoSe_2_@SPMM‐regulated macrophage microenvironment promotes fibroblast migration and ECM‐related protein expression under OS. (a) Schematic illustration of the experimental workflow. Macrophages were first exposed to H_2_O_2_ to induce OS and then treated with MoSe_2_ coated with different membranes. CCM from each macrophage group was subsequently collected and applied to fibroblasts under OS to evaluate cell migration and ECM‐related protein expression. Created with Biorender.com. (b) Representative images of scratch wound‐healing assays of fibroblasts at 0 and 24 h. The blue region indicates the wound area. Scale bar, 50 µm. (c) Quantification of fibroblast migration rates in the scratch assay. Data are presented as mean ± s.d. (n = 3 biological replicates). (d) Representative Transwell migration images showing chemotactic migration of fibroblasts cultured with CCM from different treatment groups. Migrated cells were stained with crystal violet. Scale bar, 200 µm. (e) Quantitative analysis of migrated cell numbers in the Transwell assay. Data are presented as mean ± s.d. (n = 5 biological replicates). (f) Immunofluorescence images showing the focal adhesion protein vinculin and ECM protein FN‐1 in fibroblasts. The actin cytoskeleton was stained with phalloidin (green), vinculin or FN‐1 was labelled in red, and nuclei were stained with DAPI (blue). Scale bars, 100 µm for vinculin and 50 µm for FN‐1. (g) Quantification of vinculin and FN‐1 fluorescence intensity. Data are presented as mean ± s.d. (n = 3 biological replicates). (h) Immunofluorescence images showing collagen proteins COL‐III and COL‐I in fibroblasts. The actin cytoskeleton was stained with phalloidin (green), COL‐III or COL‐I was labelled in red, and nuclei were stained with DAPI (blue). Scale bar, 100 µm. (i) Quantification of COL‐III and COL‐I fluorescence intensity. Data are presented as mean ± s.d. (n = 3 biological replicates). Images in b, f and h are representative of three biologically independent experiments; d is representative of five. Statistical significance was calculated via one‐way ANOVA with Tukey's post hoc test. OS, oxidative stress. ns, non‐significant. ^*^, *p* < 0.05. ^**^, *p* < 0.01. ^***^, *p* < 0.001. ^****^, *p* < 0.0001.

Given the central role of fibroblasts in peri‐implant STI, we next examined whether this reprogrammed macrophage microenvironment could also modulate fibroblast function. Fibroblasts are key effector cells responsible for connective tissue formation around implants, regulating ECM deposition, collagen remodeling, and soft tissue attachment. Therefore, fibroblast migration, adhesion, and matrix‐related functions were assessed to determine whether the macrophage‐regulated microenvironment could promote soft tissue reconstruction [4, 60]. Using a similar experimental workflow as for vascular endothelial cells, CCM derived from macrophages treated with different membrane‐coated MoSe_2_ was applied to fibroblasts under OS. Fibroblast migration, adhesion, and matrix‐related functions were subsequently assessed (Figure 7a). As shown in Figure 7b,c, OS markedly impaired fibroblast migration, as evidenced by delayed wound closure in the scratch assay. Treatment with MoSe_2_@SPMM‐CCM accelerated wound closure and restored migratory capacity, whereas MoSe_2_@MM‐CCM showed a moderate improvement and MoSe_2_@RBCM‐CCM had only a limited effect. This trend was further confirmed in the transwell assay (Figure 7d,e), where OS reduced the number of migrated cells, while MoSe_2_@SPMM‐CCM markedly promoted fibroblast migration. We next examined cell adhesion and ECM deposition and remodeling. As shown in Figure 7f,g, OS markedly disrupted fibroblast adhesion, with diminished vinculin and fibronectin 1 (FN‐1) signals accompanied by a disorganized cytoskeletal architecture. As core components of focal adhesions, vinculin and FN‐1 coordinate cell–matrix interactions during peri‐implant soft tissue integration. A reduction in FN‐1 deposition points to compromised matrix assembly and weakened integrin‐mediated anchorage, whereas decreased vinculin reflects impaired focal adhesion maturation and insufficient cytoskeletal stabilization [4, 61, 62]. Upon treatment with MoSe_2_@SPMM‐CCM, fibroblasts largely regained their morphology, together with a pronounced recovery of vinculin and FN‐1 expression. By comparison, MoSe_2_@MM‐CCM produced only a partial effect, while MoSe_2_@RBCM‐CCM showed little improvement. A similar trend was observed in collagen expression (Figure 7h,i). OS reduced the levels of both collagen I and collagen III, whereas treatment with MoSe_2_@SPMM‐CCM restored their expression. Collagen III is typically associated with the early phase of tissue repair, where its flexible network supports fibroblast migration and angiogenesis and provides a provisional matrix for peri‐implant STI [63, 64]. In contrast, collagen I accumulates during the remodeling phase, forming a denser and mechanically stronger fibrillar network that contributes to stable connective tissue attachment and enhances the integrity of the soft tissue seal [63, 64]. Taken together, MoSe_2_@SPMM reshapes the macrophage microenvironment by relieving OS, which in turn restores fibroblast function across migration, adhesion, and matrix remodeling under OS, thereby facilitating the establishment of the peri‐implant STI interface.

### 2.8 MoSe_2_@SPMM Enhances Peri‐Implant STI In Vivo

**FIGURE 8:**
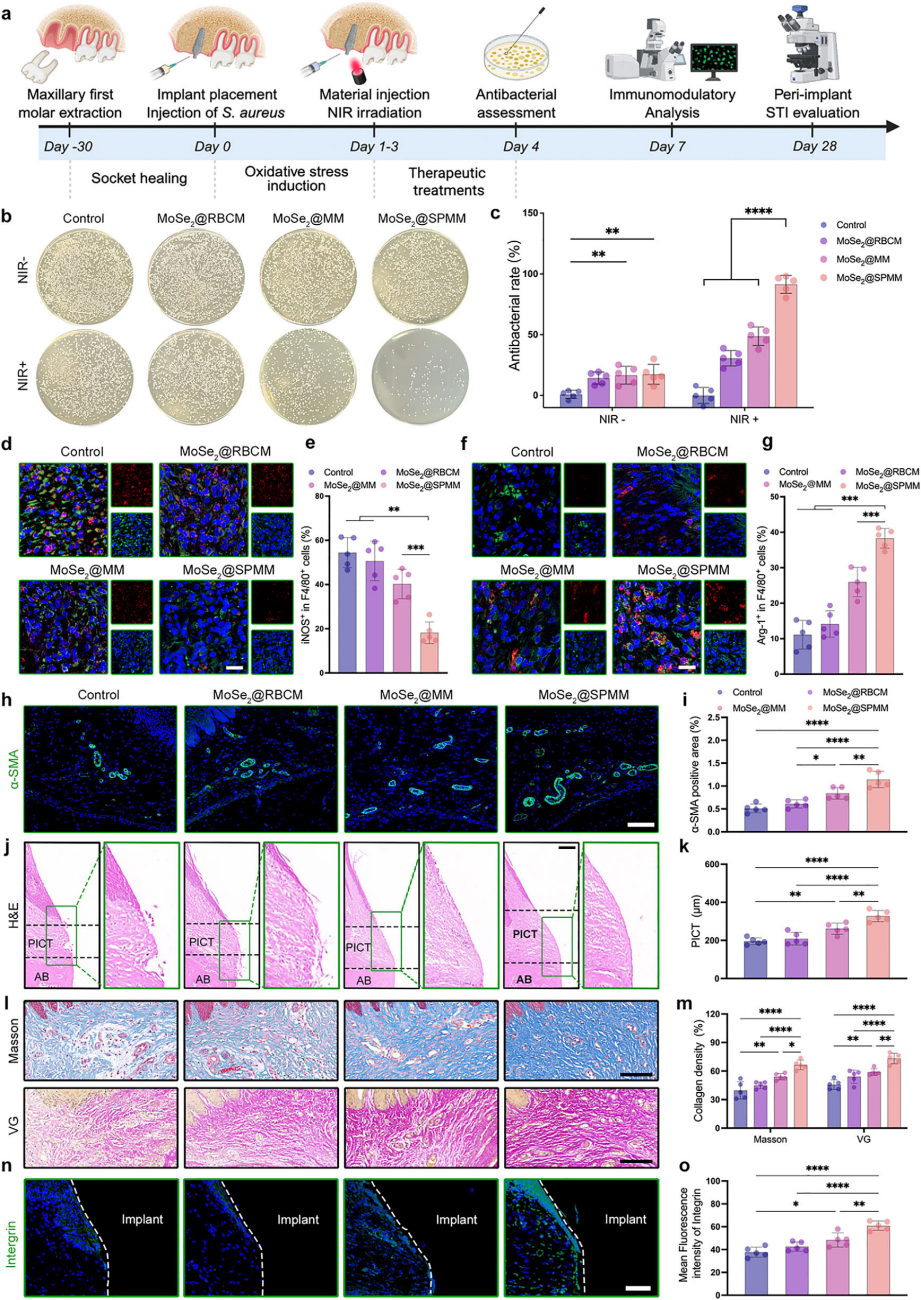
MoSe_2_@SPMM promotes a pro‐regenerative peri‐implant microenvironment and enhances peri‐implant STI in vivo. (a) Schematic diagram of the in vivo study process. After extraction of the maxillary first molars and a 30‐day healing period, implants were placed and S. aureus was inoculated to induce peri‐implant OS injury on Day 0. From Day 1 to Day 3, different nanoparticles were locally administered in combination with NIR irradiation. Antibacterial activity, immunomodulation, and peri‐implant STI were evaluated on Days 4, 7, and 28, respectively. Created with Biorender.com. Representative colony‐forming unit images (b) and corresponding antibacterial rates (c) from peri‐implant soft tissues after different treatments. Immunofluorescence staining of F4/80^+^ macrophages (green) and iNOS (red) (d), with quantification of iNOS^+^ cells among F4/80^+^ macrophages (e). Scale bar, 20 µm. Immunofluorescence staining of F4/80^+^ macrophages (green) and Arg‐1 (red) (f), with quantification of Arg‐1^+^ cells among F4/80^+^ macrophages (g). Scale bar, 20 µm. Immunofluorescence staining of α‐SMA (green) (h) and quantification of the α‐SMA‐positive area (i). Scale bar, 100 µm. H&E staining of peri‐implant soft tissues showing the overall tissue architecture (j), with quantification of PICT width (k). Scale bar, 100 µm. Masson's trichrome and VG staining of peri‐implant tissues showing collagen deposition and organization (l), with quantification of collagen density (m). Scale bars, 100 µm. Immunofluorescence staining of Integrin (green) at the implant–soft tissue interface (n), with quantification of Integrin fluorescence intensity (o). Nuclei were counterstained with DAPI (blue). Scale bar, 50 µm. Data in **c**, **e**, **g**, **i**, **k**, **m** and **o** are presented as mean ± s.d. (n = 5 biologically independent animals). Statistical significance was calculated via one‐way ANOVA with Tukey's post hoc test. Images in **d**, **f**, **h**, **j**, **l** and **n** are representative of five biologically independent animals. PICT, peri‐implant connective tissue. AB, Alveolar bone. *, *p* < 0.05. **, *p* < 0.01. ***, *p* < 0.001. ****, *p* < 0.0001.

To evaluate the in vivo potential of MoSe_2_@SPMM in enhancing peri‐implant STI, a delayed implantation model was established in the rat maxilla. At the time of implantation, *S. aureus* was locally introduced at the peri‐implant site. In addition to reflecting its role as an early colonizer in biofilm initiation, this approach enabled the establishment of a controlled OS microenvironment, thereby providing a more stringent and comparable in vivo setting for evaluating MoSe_2_@SPMM. It should be emphasized that this model was not designed to reproduce established clinical peri‐implantitis, which is typically characterized by polymicrobial biofilms and more complex disease progression. Rather, local S. aureus inoculation was used to create a controlled early oxidative‐stress challenge after implantation, reflecting early pathogenic and immunological stressors that may impair peri‐implant STI. Accordingly, MoSe_2_@SPMM is intended as an early microenvironment‐regulatory strategy to disrupt the pathogenic–immunological amplification loop, thereby enhancing STI and potentially preventing or reducing the risk of peri‐implant diseases. Following material administration and NIR irradiation, antibacterial efficacy, immunomodulation, and peri‐implant STI were systematically assessed at predefined time points (Figure 8a). In the absence of NIR irradiation, all groups exhibited only limited antibacterial activity. Upon NIR exposure, antibacterial efficacy was enhanced across all treatments, with MoSe_2_@SPMM showing the most pronounced reduction in colony counts with an antibacterial rate of 91.4%, significantly outperforming MoSe_2_@MM and MoSe_2_@RBCM (Figure 8b,c). Having confirmed the in vivo antibacterial performance of MoSe_2_@SPMM under NIR irradiation, we next investigated whether MoSe_2_@SPMM could reshape the peri‐implant immune microenvironment to enhance STI. Following the workflow shown in Figure 8a, immunomodulation was evaluated on Day 7, and peri‐implant STI was assessed on Day 28 after combined material treatment and NIR irradiation. As shown in Figure 8d,e, bacterial challenge–induced OS led to a high proportion of iNOS^+^ cells in the control group. Treatment with MoSe_2_@SPMM or MoSe_2_@MM under NIR reduced the proportion of iNOS^+^ cells, with a more pronounced decrease observed in the MoSe_2_@SPMM group, whereas MoSe_2_@RBCM showed only minimal changes. In parallel, the repair‐associated marker Arg‐1 exhibited an opposite trend (Figure 8f,g). Arg‐1^+^ cells were scarce in the control group but increased following treatment, with the highest expression observed in the MoSe_2_@SPMM group. Quantitative analysis further confirmed a significant enrichment of F4/80^+^Arg‐1^+^ cells in the MoSe_2_@SPMM group (Figure 8g).

By reducing bacterial burden, MoSe_2_@SPMM alleviates persistent microbial stimulation and promotes macrophage functional recovery, thereby creating a pro‐regenerative microenvironment that may support vascular maturation, ECM remodeling, and soft tissue seal formation. Therefore, we next evaluated peri‐implant STI at day 28. Immunofluorescence staining for alpha‐smooth muscle actin (α‐SMA), a marker of vascular maturation, showed a low abundance of α‐SMA^+^ structures in the control group, whereas the MoSe_2_@SPMM group exhibited a markedly higher density (Figure 8h). Quantification confirmed a significant increase in α‐SMA^+^ area in the MoSe_2_@SPMM group compared with other treatments, indicating enhanced vascular maturation under OS (Figure 8i). Histological evaluation by hematoxylin and eosin (H&E) staining revealed that the peri‐implant STI interface in the control and MoSe_2_@RBCM groups appeared relatively loose, with reduced peri‐implant connective tissue (PICT) width and evident epithelial downgrowth, indicative of compromised STI quality (Figure 8j). In contrast, the MoSe_2_@SPMM group exhibited a more integrated interface, characterized by continuous epithelium and increased PICT width. Quantitative analysis confirmed a significantly greater PICT width and barrier epithelium length, together with reduced inflammatory cell infiltration, in the MoSe_2_@SPMM group compared with other treatments (Figure 8k and Figure S17). Given that adequate PICT width, epithelial barrier formation, and a balanced local immune environment are critical for restricting epithelial migration and maintaining the peri‐implant biological seal [12], these results suggest that MoSe_2_@SPMM facilitates the establishment of a more well‐organized and robust STI interface. Further evaluation at the connective tissue level by Masson and Van Gieson (VG) staining revealed loosely distributed and disorganized collagen fibers in the control group (Figure 8l). In contrast, MoSe_2_@SPMM markedly enhanced collagen deposition, with improved fiber alignment and a denser, more organized architecture. MoSe_2_@MM showed partial improvement but remained inferior to MoSe_2_@SPMM. Quantitative analysis was consistent with these observations, confirming the superior capacity of MoSe_2_@SPMM in promoting collagen remodeling (Figure 8m). Beyond structural changes, we next examined cell–ECM interactions during peri‐implant soft tissue establishment. Integrins, key transmembrane receptors mediating cell–matrix adhesion, contribute to initial cell anchorage and regulate migration, proliferation, and focal adhesion maturation through cytoskeletal coupling and downstream signaling [8, 65]. As shown in Figure 8n,o, integrin expression was relatively low in the control group but increased following treatment, with the most pronounced enhancement observed in the MoSe_2_@SPMM group. These results suggest that MoSe_2_@SPMM promotes the establishment and strengthening of cell–ECM adhesion, thereby supporting the formation of more functionally favorable peri‐implant STI. Compared with conventional titanium surface modification strategies that mainly integrate antibacterial, bioactive, or immunomodulatory functions onto implant surfaces, MoSe_2_@SPMM provides a biomimetic therapeutic platform that simultaneously enables bacterial/macrophage targeting and antioxidative regulation. Moreover, its locally administrable design offers greater clinical flexibility and may mitigate limitations associated with surface‐bound strategies, including insufficient stability in the complex oral environment, uncontrolled release of bioactive agents, and limited long‐term efficacy [3, 66].

## 3 Conclusions

In summary, we developed a biomimetic nanoengineered platform (MoSe_2_@SPMM) that integrates pathogen‐targeting capability with immunoregulatory function for the management of peri‐implant microenvironments. This design pairs the *S. aureus*–targeting capability of SPMM with the photothermal activity of MoSe_2_ to enable targeted contact‐enhanced bacterial eradication, while concurrently coupling macrophage‐targeting of SPMM with the ROS‐scavenging activity of MoSe_2_ to alleviate OS–induced dysfunction in macrophages. Through this dual‐functional integration, MoSe_2_@SPMM coordinates antibacterial activity with immune modulation, reshaping the local microenvironment and preserving vascular endothelial cell and fibroblast functions related to angiogenesis, migration, and ECMremodeling. As a result, it facilitates the formation of a more organized and functionally favorable peri‐implant STI in vivo. More broadly, this design principle may be extendable to other implant‐associated soft tissue interfaces, including orthopaedic and percutaneous devices, where bacterial colonization, oxidative stress, and dysregulated inflammation jointly compromise tissue repair. Collectively, this work highlights a strategy that links targeted antibacterial action with redox‐mediated immune regulation to disrupt the pathogenic–immunological amplification loop, offering a promising approach for enhancing peri‐implant soft tissue integration.

From a translational perspective, MoSe_2_@SPMM may be suitable for local application at the implant–soft tissue interface, including the region between the transgingival implant neck and surrounding soft tissue in tissue‐level implants, as well as the interface between the transmucosal abutment and soft tissue in bone‐level implants. In potential dental implant applications, MoSe_2_@SPMM could be locally delivered around these soft tissue integration sites during the early healing stage or under high‐risk inflammatory conditions, followed by localized NIR activation using intraoral NIR probes or fiber‐guided irradiation. This site‐specific intervention may help concentrate antibacterial and immunoregulatory effects at the peri‐implant soft tissue seal while limiting unnecessary systemic exposure. Further refinement of the treatment window, irradiation parameters, repeated local delivery protocols, scalable membrane‐coating preparation, and long‐term in vivo fate will facilitate its future clinical translation.

## Author Contributions

**Minghao Zhou**: conceptualization, methodology, writing – original draft, writing – review and editing, investigation, visualization, software. **Miaomiao Tian**: methodology, validation, data curation, investigation, visualization. **Jingwei Yu**: methodology, visualization. **Jiaxin Kang**: methodology, data curation, validation. **Miaomiao Chen**: formal analysis, validation, investigation. **Yue Yuan**: investigation. **Lizhi Liu**: writing – review and editing, supervision, resources, project administration, conceptualization. **Hongbo Wei**: conceptualization, writing – review and editing, funding acquisition, project administration, supervision, resources.

## Conflicts of Interest

The authors declare no conflicts of interest.

## Ethics Statement

All animal experiments were conducted in accordance with institutional guidelines for the care and use of laboratory animals and protocols, which were approved by the Institutional Animal Care and Use Committee, Fourth Military Medical University (Approval No. kq‐2025‐166).

## Supporting information




**Supporting File**: advs77771‐sup‐0001‐SuppMat.pdf.

## Data Availability

The data that support the findings of this study are available from the corresponding author upon reasonable request.

## References

[1] M. E. Galarraga‐Vinueza , S. Pagni , M. Finkelman , T. Schoenbaum , and L. Chambrone , “Prevalence, Incidence, Systemic, Behavioral, and Patient‐Related Risk Factors and Indicators for Peri‐Implant Diseases: An AO/AAP Systematic Review and Meta‐Analysis,” Journal of Periodontology 96, no. 6 (2025): 587–633, 10.1002/JPER.24-0154. PMC12273760 40489307

[2] Grand View Research. *Peri‐implantitis Market (2025‐2030): Size, Share & Trends Analysis Report by Method Type and Region* , Updated July 2026. Accessed April 2026, https://www.grandviewresearch.com/industry‐analysis/peri‐implantitis‐market.

[3] S. Jin , Y. Yu , T. Zhang , et al., “Surface Modification Strategies to Reinforce the Soft Tissue Seal at Transmucosal Region of Dental Implants,” Bioactive Materials 42 (2024): 404–432, 10.1016/j.bioactmat.2024.08.042. PMC11415887 39308548

[4] R. Alexander and X. Liu , “Soft Tissue Integration Around Dental Implants: A Pressing Priority,” Biomaterials 324 (2026): 123491, 10.1016/j.biomaterials.2025.123491. PMC12226153 40505390

[5] T. Guo , K. Gulati , H. Arora , P. Han , B. Fournier , and S. Ivanovski , “Orchestrating Soft Tissue Integration at the Transmucosal Region of Titanium Implants,” Acta Biomaterialia 124 (2021): 33–49, 10.1016/j.actbio.2021.01.001. 33444803

[6] J. Lindhe and J. Meyle , “Peri‐Implant Diseases: Consensus Report of the Sixth European Workshop on Periodontology,” Journal of Clinical Periodontology 35, no. s8 (2008): 282–285, 10.1111/j.1600-051X.2008.01283.x. 18724855

[7] “Academy Report: Peri‐Implant Mucositis and Peri‐Implantitis: A Current Understanding of Their Diagnoses and Clinical Implications,” Journal of Periodontology 84, no. 4 (2013): 436–443, 10.1902/jop.2013.134001. 23537178

[8] G. Shineh , L. M. Janghour , Y. Xia , et al., “Biomolecule‐Functionalized Dental Implant Surfaces: Towards Augmenting Soft Tissue Integration,” Bioactive Materials 53 (2025): 540–590, 10.1016/j.bioactmat.2025.07.005. PMC12313982 40755849

[9] Y. Yuan , Z. Hou , M. Chen , et al., “Sequential Hydrogen‐Release Implant for Promoting the Soft Tissue Integration Through Immunomodulatory and Pro‐Remodeling Coupling,” Bioactive Materials 57 (2026): 514–530, 10.1016/j.bioactmat.2025.11.018. PMC12666708 41334374

[10] W.‐R. Wang , J. Li , J.‐T. Gu , et al., “Optimization of Lactoferrin‐Derived Amyloid Coating for Enhancing Soft Tissue Seal and Antibacterial Activity of Titanium Implants,” Advanced Healthcare Materials 12, no. 11 (2023): 2203086, 10.1002/adhm.202203086. 36594680

[11] R. Liu , S. Chen , P. Huang , et al., “Immunomodulation‐Based Strategy for Improving Soft Tissue and Metal Implant Integration and Its Implications in the Development of Metal Soft Tissue Materials,” Advanced Functional Materials 30, no. 21 (2020): 1910672, 10.1002/adfm.201910672.

[12] A. Harada , H. Sasaki , Y. Asami , et al., “Effects of the Application of Low‐Temperature Atmospheric Plasma on Titanium Implants on Wound Healing in Peri‐Implant Connective Tissue in Rats,” International Journal of Implant Dentistry 10, no. 1 (2024): 15, 10.1186/s40729-024-00524-3. PMC10954594 38509336

[13] T. Berglundh , G. Armitage , M. G. Araujo , et al., “Peri‐Implant Diseases and Conditions: Consensus Report of Workgroup 4 of the 2017 World Workshop on the Classification of Periodontal and Peri‐Implant Diseases and Conditions,” Journal of Clinical Periodontology 45, no. S20 (2018): S286–S291, 10.1111/jcpe.12957. 29926491

[14] T. Guo , K. Gulati , H. Arora , P. Han , B. Fournier , and S. Ivanovski , “Race to Invade: Understanding Soft Tissue Integration at the Transmucosal Region of Titanium Dental Implants,” Dental Materials 37, no. 5 (2021): 816–831, 10.1016/j.dental.2021.02.005. 33676764

[15] C. R. Arciola , D. Campoccia , P. Speziale , L. Montanaro , and J. W. Costerton , “Biofilm Formation in Staphylococcus Implant Infections. A Review of Molecular Mechanisms and Implications for Biofilm‐Resistant Materials,” Biomaterials 33, no. 26 (2012): 5967–5982, 10.1016/j.biomaterials.2012.05.031. 22695065

[16] C. R. Arciola , D. Campoccia , and L. Montanaro , “Implant Infections: Adhesion, Biofilm Formation and Immune Evasion,” Nature Reviews Microbiology 16, no. 7 (2018): 397–409, 10.1038/s41579-018-0019-y. 29720707

[17] M. E. Galarraga‐Vinueza , K. Obreja , A. Ramanauskaite , et al., “Macrophage Polarization in Peri‐Implantitis Lesions,” Clinical Oral Investigations 25, no. 4 (2021): 2335–2344, 10.1007/s00784-020-03556-2. PMC7966129 32886246

[18] F. Schwarz , J. Derks , A. Monje , and H.‐L. Wang , “Peri‐Implantitis,” Journal of Clinical Periodontology 45, no. S20 (2018): S246–S266, 10.1111/jcpe.12954. 29926484

[19] T. A. Wynn and K. M. Vannella , “Macrophages in Tissue Repair, Regeneration, and Fibrosis,” Immunity 44, no. 3 (2016): 450–462, 10.1016/j.immuni.2016.02.015. PMC4794754 26982353

[20] Y. Feng , J. Chen , and C. Ning , “Advances in Biomedical Applications of Se‐Based Nanozymes,” Journal of Nanobiotechnology 23, no. 1 (2025): 711, 10.1186/s12951-025-03770-8. PMC12604428 41214729

[21] R. Zhang , B. Jiang , K. Fan , L. Gao , and X. Yan , “Designing Nanozymes for In Vivo Applications,” Nature Reviews Bioengineering 2, no. 10 (2024): 849–868, 10.1038/s44222-024-00205-1.

[22] X. Du , Z. Dong , Y. Yan , et al., “Immunomodulatory Nanozymes Eradicate Intracellular Infections and Rescue Immunoparalysis for Treating Multidrug‐Resistant Bacterial Sepsis,” Exploration 5, no. 5 (2025): 20250127, 10.1002/EXP.20250127. PMC12561197 41163807

[23] B. Li , W. Wang , L. Zhao , et al., “Photothermal Therapy of Tuberculosis Using Targeting Pre‐Activated Macrophage Membrane‐Coated Nanoparticles,” Nature Nanotechnology 19, no. 6 (2024): 834–845, 10.1038/s41565-024-01618-0. 38383890

[24] J. Zhu , R. Xie , R. Gao , et al., “Multimodal Nanoimmunotherapy Engages Neutrophils to Eliminate Staphylococcus Aureus Infections,” Nature Nanotechnology 19, no. 7 (2024): 1032–1043, 10.1038/s41565-024-01648-8. PMC12126137 38632494

[25] Y. Liu , J. Luo , X. Chen , W. Liu , and T. Chen , “Cell Membrane Coating Technology: A Promising Strategy for Biomedical Applications,” Nano‐Micro Letters 11, no. 1 (2019): 100, 10.1007/s40820-019-0330-9. PMC7770915 34138027

[26] S. Liu , H. Wang , L. Yu , et al., “Rapid Bacterial Detection and Gram‐Identification Using Bacterially Activated, Macrophage‐Membrane‐Coated Nanowired‐Si Surfaces in a Microfluidic Device,” Nano Letters 23, no. 17 (2023): 8326–8330, 10.1021/acs.nanolett.3c02686. PMC10510579 37611221

[27] J. Wang , C. Zhu , J. Tan , J. Xu , and C. Wang , “Engineered Artificial Nanochannels With Cell Membrane Nanointerface for Ultrasensitive Detection and Discrimination of Multiple Bacterial Infections,” Journal of the American Chemical Society 147, no. 15 (2025): 12821–12832, 10.1021/jacs.5c01542. 40173403

[28] R. Shang , F. Yang , G. Gao , et al., “Bioimaging and Prospects of Night Pearls‐Based Persistence Phosphors in Cancer Diagnostics,” Exploration 4, no. 4 (2024): 20230124, 10.1002/EXP.20230124. PMC11335470 39175886

[29] C. L. Mitchell , M. Matveyenka , and D. Kurouski , “Neuroprotective Properties of Transition Metal Dichalcogenide Nanoflowers Alleviate Acute and Chronic Neurological Conditions Linked to Mitochondrial Dysfunction,” Journal of Biological Chemistry 301, no. 5 (2025): 108498, 10.1016/j.jbc.2025.108498. PMC12139420 40216249

[30] X. Zheng , Y. Ouyang , H. Fan , et al., “Molybdesum Selenide‐Based Platelet‐Rich Plasma Containing Carboxymethyl Chitosan/Polyvinyl Pyrrolidone Composite Antioxidant Hydrogels Dressing Promotes the Wound Healing,” Journal of Nanobiotechnology 22, no. 1 (2024): 217, 10.1186/s12951-024-02490-9. PMC11080249 38725012

[31] P. Xie , L. Zhang , H. Shen , et al., “Biodegradable MoSe_2_‐Polyvinylpyrrolidone Nanoparticles With Multi‐Enzyme Activity for Ameliorating Acute Pancreatitis,” Journal of Nanobiotechnology 20, no. 1 (2022): 113, 10.1186/s12951-022-01288-x. PMC8898412 35248068

[32] Y. Wang , F. Zhang , Q. Wang , P. Yang , H. Lin , and F. Qu , “Hierarchical MoSe_2_ Nanoflowers as Novel Nanocarriers for Nir‐Light‐Mediated Synergistic Photo‐Thermal/Dynamic and Chemo‐Therapy,” Nanoscale 10, no. 30 (2018): 14534–14545, 10.1039/C8NR04538K. 30024001

[33] M. P. Rayman , “The Importance of Selenium to Human Health,” The Lancet 356, no. 9225 (2000): 233–241, 10.1016/S0140-6736(00)02490-9. 10963212

[34] J. A. Novotny and C. A. Peterson , “Molybdenum,” Advances in Nutrition 9, no. 3 (2018): 272–273, 10.1093/advances/nmx001. PMC5952949 29767695

[35] L. G. Harris and R. G. Richards , “Staphylococci and Implant Surfaces: A Review,” Injury 37, no. 2 (2006): S3–S14, 10.1016/j.injury.2006.04.003. 16651069

[36] G. E. Salvi , M. M. Fürst , N. P. Lang , and G. R. Persson , “One‐Year Bacterial Colonization Patterns of Staphylococcus Aureus and Other Bacteria at Implants and Adjacent Teeth,” Clinical Oral Implants Research 19, no. 3 (2008): 242–248, 10.1111/j.1600-0501.2007.01470.x. 18177429

[37] L.‐F. Zhuang , R. M. Watt , N. Mattheos , M.‐S. Si , H.‐C. Lai , and N. P. Lang , “Periodontal and Peri‐Implant Microbiota in Patients With Healthy and Inflamed Periodontal and Peri‐Implant Tissues,” Clinical Oral Implants Research 27, no. 1 (2016): 13–21, 10.1111/clr.12508. 25399962

[38] M. Kronström , B. Svensson , E. Erickson , L. Houston , P. Braham , and G. R. Persson , “Humoral Immunity Host Factors in Subjects with Failing or Successful Titanium Dental Implants,” Journal of Clinical Periodontology 27, no. 12 (2000): 875–882, 10.1034/j.1600-051x.2000.027012875.x. 11140553

[39] M. Kronström , B. Svenson , M. Hellman , and G. R. Persson , “Early Implant Failures in Patients Treated with Brånemark System Titanium Dental Implants: A Retrospective Study,” International Journal of Oral and Maxillofacial Implants 16, no. 2 (2001): 201–207. 11324208

[40] G. R. Persson and S. Renvert , “Cluster of Bacteria Associated With Peri‐Implantitis,” Clinical Implant Dentistry and Related Research 16, no. 6 (2014): 783–793, 10.1111/cid.12052. 23527870

[41] A. Plüddemann , S. Mukhopadhyay , and S. Gordon , “The Interaction of Macrophage Receptors With Bacterial Ligands,” Expert Reviews in Molecular Medicine 8, no. 28 (2006): 1–25, 10.1017/S1462399406000159. 17118220

[42] T. Kawai and S. Akira , “Toll‐Like Receptors and Their Crosstalk With Other Innate Receptors in Infection and Immunity,” Immunity 34, no. 5 (2011): 637–650, 10.1016/j.immuni.2011.05.006. 21616434

[43] P. R. Taylor , L. Martinez‐Pomares , M. Stacey , H.‐H. Lin , G. D. Brown , and S. Gordon , “Macrophage Receptors and Immune Recognition,” Annual Review of Immunology 23, no. 1 (2005): 901–944, 10.1146/annurev.immunol.23.021704.115816. 15771589

[44] J. Li , Y. Wang , J. Yang , and W. Liu , “Bacteria Activated‐Macrophage Membrane‐Coated Tough Nanocomposite Hydrogel With Targeted Photothermal Antibacterial Ability for Infected Wound Healing,” Chemical Engineering Journal 420 (2021): 127638, 10.1016/j.cej.2020.127638.

[45] S. Liu , G. Jiang , R. Shi , et al., “Clearance of Eskape Pathogens From Blood Using Bacterially Activated Macrophage Membrane‐Coated Silicon Nanowires,” Advanced Functional Materials 31, no. 14 (2021): 2007613, 10.1002/adfm.202007613.

[46] L. M. Stuart , J. Deng , J. M. Silver , et al., “Response to Staphylococcus Aureus Requires Cd36‐Mediated Phagocytosis Triggered by the Cooh‐Terminal Cytoplasmic Domain,” The Journal of Cell Biology 170, no. 3 (2005): 477–485, 10.1083/jcb.200501113. PMC2171464 16061696

[47] A. Ozinsky , D. M. Underhill , J. D. Fontenot , et al., “The Repertoire for Pattern Recognition of Pathogens by the Innate Immune System is Defined by Cooperation between Toll‐Like Receptors,” Proceedings of the National Academy of Sciences 97, no. 25 (2000): 13766–13771, 10.1073/pnas.250476497. PMC17650 11095740

[48] J. Y. Kang , X. Nan , M. S. Jin , et al., “Recognition of Lipopeptide Patterns by Toll‐Like Receptor 2‐Toll‐Like Receptor 6 Heterodimer,” Immunity 31, no. 6 (2009): 873–884, 10.1016/j.immuni.2009.09.018. 19931471

[49] P.‐A. Oldenborg , A. Zheleznyak , Y.‐F. Fang , C. F. Lagenaur , H. D. Gresham , and F. P. Lindberg , “Role of Cd47 as a Marker of Self on Red Blood Cells,” Science 288, no. 5473 (2000): 2051–2054, 10.1126/science.288.5473.2051. 10856220

[50] A. N. Barclay and T. K. van den Berg , “The Interaction Between Signal Regulatory Protein Alpha (Sirpα) and Cd47: Structure, Function, and Therapeutic Target,” Annual Review of Immunology 32, no. 1 (2014): 25–50, 10.1146/annurev-immunol-032713-120142. 24215318

[51] L. K. Erdman , G. Cosio , A. J. Helmers , D. C. Gowda , S. Grinstein , and K. C. Kain , “CD36 and TLR Interactions in Inflammation and Phagocytosis: Implications for Malaria,” The Journal of Immunology 183, no. 10 (2009): 6452–6459, 10.4049/jimmunol.0901374. PMC2853812 19864601

[52] J. M. Blander and R. Medzhitov , “Regulation of Phagosome Maturation by Signals From Toll‐Like Receptors,” Science 304, no. 5673 (2004): 1014–1018, 10.1126/science.1096158. 15143282

[53] Q. Taban , P. T. Mumtaz , K. Z. Masoodi , E. Haq , and S. M. Ahmad , “Scavenger Receptors in Host Defense: From Functional Aspects to Mode of Action,” Cell Communication and Signaling 20, no. 1 (2022): 2, 10.1186/s12964-021-00812-0. PMC8721182 34980167

[54] Y. Gao , L. Rong , J. Cui , et al., “Artificial Lipids and Macrophage Membranes Coassembled Biomimetic Nanovesicles for Thoracic Aortic Dissection Treatment,” Journal of Controlled Release 383 (2025): 113844, 10.1016/j.jconrel.2025.113844. 40379216

[55] A. Parodi , N. Quattrocchi , A. L. van de Ven , et al., “Synthetic Nanoparticles Functionalized With Biomimetic Leukocyte Membranes Possess Cell‐Like Functions,” Nature Nanotechnology 8, no. 1 (2013): 61–68, 10.1038/nnano.2012.212. PMC3751189 23241654

[56] W. Xue , W. Zheng , H. Zhang , et al., “Dual‐Targeted Metabolic Nanovesicles Reprogram Macrophage‐Cardiomyocyte Crosstalk for Myocardial Ischemia‐Reperfusion Therapy,” Advanced Functional Materials 36, no. 12 (2026): 07784, 10.1002/adfm.202507784.

[57] J. Xiong , H. Tang , L. Sun , et al., “A Macrophage Cell Membrane‐Coated Cascade‐Targeting Photothermal Nanosystem for Combating Intracellular Bacterial Infections,” Acta Biomaterialia 175 (2024): 293–306, 10.1016/j.actbio.2023.12.045. 38159895

[58] L. Fan , C. Zhang , Z. Xu , et al., “Hybrid Macrophage‐Mitochondria Extracellular Vesicles for Mitochondrial Ros Regulation in Diabetic Wounds,” Nature Communications 17, no. 1 (2026): 3285, 10.1038/s41467-026-69383-3. PMC13066382 41760650

[59] S. T. Smiley , M. Reers , C. Mottola‐Hartshorn , et al., “Intracellular Heterogeneity in Mitochondrial Membrane Potentials Revealed by a J‐Aggregate‐Forming Lipophilic Cation Jc‐1,” Proceedings of the National Academy of Sciences 88, no. 9 (1991): 3671–3675, 10.1073/pnas.88.9.3671. PMC51514 2023917

[60] F. Aellos , A. Clavelier , B. Liu , et al., “Soft‐Tissue Integration of Dental Implants: Formation, Maintenance, and Relevance for Peri‐Implant Health,” Journal of Periodontal Research (2026): 1–15, 10.1111/jre.70091. 41883086

[61] Q. Zhao , Z. Zhao , J. Zhang , et al., “Fn‐HMGB1 Adsorption Behavior Initiates Early Immune Recognition and Subsequent Osteoinduction of Biomaterials,” Advanced Healthcare Materials 13, no. 2 (2024): 2301808, 10.1002/adhm.202301808. 37602504

[62] M. Pagel , R. Hassert , T. John , et al., “Multifunctional Coating Improves Cell Adhesion on Titanium by Using Cooperatively Acting Peptides,” Angewandte Chemie International Edition 55, no. 15 (2016): 4826–4830, 10.1002/anie.201511781. 26938787

[63] Z. Zheng , X. Ao , P. Xie , F. Jiang , and W. Chen , “The Biological Width Around Implant,” Journal of Prosthodontic Research 65, no. 1 (2021): 11–18, 10.2186/jpr.JPOR_2019_356. 32938861

[64] I. Atsuta , Y. Ayukawa , R. Kondo , et al., “Soft Tissue Sealing Around Dental Implants Based on Histological Interpretation,” Journal of Prosthodontic Research 60, no. 1 (2016): 3–11, 10.1016/j.jpor.2015.07.001. 26725967

[65] N. G. Fischer and C. Aparicio , “Junctional Epithelium and Hemidesmosomes: Tape and Rivets for Solving the “Percutaneous Device Dilemma” in Dental and Other Permanent Implants,” Bioactive Materials 18 (2022): 178–198, 10.1016/j.bioactmat.2022.03.019. PMC8961425 35387164

[66] Y. Yuan , M. Zhou , J. Yu , M. Chen , J. Kang , and H. Wei , “The “Barrier‐Erecting” of Titanium Dental Implant: Surface Modification Strategies for Enhancing Soft Tissue Integration,” Biomaterials 326 (2026): 123697, 10.1016/j.biomaterials.2025.123697. 40966911

