# Supplementary material for: Dual‐Targeted Biomimetic MoSe2 Nanozymes for Enhancing Peri‐Implant Soft Tissue Integration
Source: Adv Sci (Weinh). 2026 Sep 21:e77771. Online ahead of print. doi: 10.1002/advs.77771 (PMC13591435; doi:10.1002/advs.77771)
Supplement: Supplementary file 1 [file ADVS-9999-e77771-s001.pdf]

Supporting Information

Dual-Targeted Biomimetic $MoSe_2$ Nanozymes for Enhancing Peri-Implant Soft Tissue Integration

Minghao Zhou[1], Miaomiao Tian[1], Jingwei Yu[1], Jiaxin Kang[1], Miaomiao Chen[1], Yue Yuan[1], Lizhi Liu[2] and Hongbo Wei[1]**

[1]State Key Laboratory of Oral & Maxillofacial Reconstruction and Regeneration, National Clinical Research Center for Oral Diseases, Shaanxi Engineering Research Center for Dental Materials and Advanced Manufacture, Department of Oral Implants, School of Stomatology, The Fourth Military Medical University, Xi'an, Shaanxi, China

[2]State Key Laboratory of Solidification Processing, School of Materials Science and Engineering, Northwestern Polytechnical University, Xi'an 710072, China

*Corresponding authors: lizhi.liu@nwpu.edu.cn; weihongbo@fmmu.edu.cn

1. Materials and Methods ***3***

Figure S1 | Effect of S. aureus pre-stimulation duration on the viability of RAW264.7 macrophages. ***22***

Figure S2 |Relative mRNA expression levels in macrophages at 0, 12, and 24 h after S. aureus stimulation were analyzed by RT–qPCR. ***23***

Figure S3 | Quantitative analysis of TLR2, TLR6, and CD36 protein expression in macrophages upon S. aureus stimulation. ***24***

Figure S4 |Quantitative analysis of immunofluorescence intensity of TLR2, TLR6, and CD36 in macrophages at different time points following S. aureus stimulation. ***25***

Figure S5 | Western blotting analysis of TLR2, TLR6 and CD6 in the $MoSe_2$, $MoSe_2$@MM, and $MoSe_2$@SPMM. ***26***

Figure S6 | Stability assessment of $MoSe_2$@SPMM in physiological media. ***27***

Figure S7 | Cyclic H_2O_2 decomposition performance of $MoSe_2$@SPMM. ***28***

Figure S8 | Catalytic recyclability and chemical stability of $MoSe_2$@SPMM during GPx-like reactions. ***29***

Figure S9 | Evaluation of ROS-scavenging of $MoSe_2$-based nanomaterials. ***30***

Figure S10 | Photothermal performance of $MoSe_2$@SPMM under 808 nm NIR irradiation. ***31***

Figure S11 | Biosafety evaluation of $MoSe_2$@SPMM. ***32***

Figure S12 | Western blotting analysis of TLR2, TLR6 and CD6 in the $MoSe_2$@RBCM, $MoSe_2$@MM, and $MoSe_2$@SPMM. ***34***

Figure S13 | Evaluation of S. aureus binding by $MoSe_2$@SPMM using $MoSe_2$@DOPC as a neutral lipid-coated control. ***35***

Figure S14 | $MoSe_2$@SPMM inhibits S. aureus biofilm formation under NIR irradiation. ***36***

Figure S15 | Macrophage uptake of $MoSe_2$@SPMM compared with $MoSe_2$@DOPC. ***37***

Figure S16 | Quantitative analysis of tube formation of HUVECs treated with conditioned medium from different groups ***38***

Figure S17 | Quantitative analysis of inflammatory cell infiltration and barrier epithelium formation in peri-implant soft tissues. ***39***

Table S1. Primer sequences used for RT–qPCR analysis 40
References in supporting information 41

1. Materials and Methods

1.1 Materials

NIH 3T3 cells (RRID:CVCL_0594), RAW 264.7 cells (RRID:CVCL_0493), human umbilical vein endothelial cells (HUVECs, RRID:CVCL_2959), and *Staphylococcus aureus* strain (ATCC 25923) were obtained from the State Key Laboratory of Oral & Maxillofacial Reconstruction and Regeneration, Xi'an, Shaanxi, China.Primary antibodies against mouse TLR2 (CST, #13744, RRID:AB_2798308), CD36 (Proteintech, #32993-1-AP, RRID:AB_3742815), and TLR6 (Proteintech, #22240-1-AP, RRID:AB_11232032) were used for Western blotting and immunofluorescence analysis. HRP-conjugated goat anti-rabbit IgG secondary antibody (Proteintech, RGAR001, RRID:AB_3073505), Alexa Fluor 488-conjugated goat anti-rabbit antibody (Immunoway, RS3211, RRID:AB_3751837), and Alexa Fluor 647-conjugated secondary antibodies (Signalway Antibody, L3018/L3038) were applied for fluorescence detection. The following assay kits were used according to the manufacturers' protocols: Reverse Transcription Kit (Kang Century, Shanghai, China), Micro Glutathione Peroxidase (GPx) Assay Kit (Solarbio, BC1195), H_2O_2 assay kit (Beyotime, S0038), total superoxide dismutase (SOD) activity assay kit (NBT method, Beyotime, S0109), hydroxyl radical scavenging assay kit (Solarbio, BC1320), DPPH radical scavenging assay kit (Solarbio, BC4750), ROS detection kit (Beyotime, S0033M), JC-1 mitochondrial membrane potential assay kit (Aikeri Biotech, AG51009), EdU-555 cell proliferation kit (Beyotime, C0075S), CCK-8 cell viability assay kit (Saint-Bio, ST1006), and Calcein-AM/PI live-dead staining kit (Beyotime, C015S). ELISA kits for mouse IL-6 (AE20012M), TNF-α (AER21997M), IL-10 (AE20005M), and TGF-β (AE21903M) were purchased from A&E Bio (China). For proteomic analysis, mass-spectrometry-grade reagents including modified trypsin (Promega), Pierce™ BCA Protein Assay Kit (Thermo Fisher Scientific), TMT 16-plex labeling reagents (Thermo Fisher Scientific), Bond-Breaker™ TCEP solution

(Thermo Fisher Scientific), LC/MS-grade water, acetonitrile, methanol, and formic acid (Thermo Fisher Scientific) were used.

1.2 Screening of Macrophage Pre-activation Time by *Staphylococcus aureus*

RAW264.7 macrophages were seeded in 6-well plates and cultured until reaching moderate confluence. The cells were then stimulated with *Staphylococcus aureus (S.aureus)* at a multiplicity of infection (MOI) of 10 for 4 h. After bacterial stimulation, the culture medium was replaced with complete medium containing antibiotics to terminate the infection. Cells were subsequently incubated for additional time periods (6, 12, 24, 36, and 48 h). At each time point, cell viability was assessed using a CCK-8 assay according to the manufacturer's protocol to evaluate the effect of different bacterial pre-stimulation durations on macrophage viability.

1.3 Proteomic Analysis

For proteomic sequencing, RAW264.7 macrophages stimulated with *S. aureus* for 24 h were used as the experimental group, while untreated macrophages cultured for the same duration served as controls. After reaching the designated incubation time, cells were gently washed with pre-chilled sterile PBS and collected using a cell scraper. The cell suspension was transferred into centrifuge tubes and washed once more with cold PBS. Samples were centrifuged at 1000 g for 10 min at 4 °C to collect the cell pellet, and the supernatant was discarded. The collected pellets were rapidly frozen in liquid nitrogen for 5 min and stored at −80 °C until further analysis. For protein extraction, frozen samples were thawed on ice and lysed with protein lysis buffer (8 M urea and 1% SDS supplemented with protease inhibitors). The lysates were subjected to non-contact ultrasonication at low temperature for 30 min, followed by centrifugation at 14 000 g for 15 min at 8 °C. The supernatant containing soluble proteins was collected. Protein concentration was determined using the BCA assay according to the manufacturer's instructions. After quantification, protein samples were subjected to SDS-PAGE electrophoresis for preliminary quality assessment.

For enzymatic digestion, 100 μg of protein from each sample was first adjusted with triethylammonium bicarbonate (TEAB) buffer to obtain a final TEAB concentration of 100 mM. Tris(2-carboxyethyl)phosphine (TCEP) was then added to a final concentration of 10 mM and incubated at 37 °C for 60 min to reduce disulfide bonds. Subsequently, iodoacetamide (IAM) was introduced at a final concentration of 40 mM and allowed to react in the dark at room temperature

for 40 min to alkylate cysteine residues. Cold acetone was added to each sample at a volume ratio of 6:1 (acetone:sample, v/v), and the mixture was incubated at −20 °C for 4 h to precipitate proteins. After precipitation, samples were centrifuged at 10 000 g for 20 min and the resulting pellet was collected. The pellet was redissolved in 100 µL of 100 mM TEAB. Trypsin was then added at an enzyme-to-protein ratio of 1:50 (w/w), and the samples were incubated at 37 °C overnight for complete digestion.

Following trypsin digestion, peptide mixtures were dried using a vacuum concentrator. The dried peptides were subsequently reconstituted in 0.1% trifluoroacetic acid (TFA). Peptide desalting was performed using HLB solid-phase extraction columns, after which the eluates were again dried by vacuum centrifugation. Peptide concentrations were determined by ultraviolet spectrophotometry using a NanoDrop One spectrophotometer (Thermo Scientific). Equal amounts of peptide samples were dissolved in mass spectrometry loading buffer and subjected to data-independent acquisition (DIA) analysis. Peptide separation was performed using a Vanquish Neo UHPLC system (Thermo Fisher Scientific, USA) equipped with a uPAC High Throughput column (75 µm × 5.5 cm, Thermo Fisher Scientific). Mobile phase A consisted of water containing 2% acetonitrile and 0.1% formic acid, whereas mobile phase B consisted of water containing 80% acetonitrile and 0.1% formic acid. The total chromatographic run time was 8 min. Mass spectrometry data were acquired using Thermo Xcalibur 4.7 software. Peptides separated by nano-flow UHPLC were analyzed on an Orbitrap Astral mass spectrometer (Thermo Fisher Scientific, USA). The instrument was operated in DIA mode under positive ionization conditions with an ion source voltage of 1.5 kV. The mass scan range was set from 100 to 1700 m/z. Raw DIA data were processed using Spectronaut™ 19 software for database searching against the UniProt protein database. The search parameters were set as follows: peptide length 7–52 amino acids; enzyme specificity trypsin/P; maximum missed cleavages 2; variable modifications including oxidation (M) and acetylation (protein N-terminus); fixed modification carbamidomethylation (C). The false discovery rate (FDR) thresholds were set at ≤0.01 for both proteins and peptides, with peptide confidence ≥99%. The extracted ion chromatogram (XIC) width was set to ≤75 ppm. Protein quantification was performed using the MaxLFQ algorithm.

1.4 Bioinformatics Analysis

The processed proteomic datasets were uploaded to the Majorbio Cloud platform (cloud.majorbio.com) for downstream bioinformatics analysis.[1,2] Statistical significance between groups was evaluated using the t.test function in R software. Fold change (FC) values were calculated to assess differences in protein expression levels. Proteins with $p < 0.05$ and fold change >1.2 were defined as differentially expressed proteins. Functional annotation of the differential proteins was performed using the Gene Ontology (GO) database (http://geneontology.org), including biological process, cellular component, and molecular function categories. Pathway enrichment analysis was conducted using the Kyoto Encyclopedia of Genes and Genomes (KEGG) database (http://www.genome.jp/kegg). In addition, Gene Set Enrichment Analysis (GSEA) was applied to the quantitative proteomic dataset. All quantifiable proteins were first ranked based on their expression differences between the two groups. Enrichment scores (ES) were calculated using predefined gene sets from the MSigDB database. Statistical significance and normalized enrichment scores (NES) were obtained through permutation testing to identify significantly enriched biological pathways.

1.5 Real-time quantitative polymerase chain reaction (RT-qPCR)

After *S. aureus* stimulation, RAW 264.7 cells were washed with PBS and further cultured in fresh complete medium containing antibiotics to terminate bacterial exposure. Cells were collected at 0, 12, and 24 h for gene expression analysis. Total RNA was extracted using TRIzol reagent according to the manufacturer's instructions. The concentration and purity of RNA were determined using a NanoDrop spectrophotometer. Subsequently, 1 μg of total RNA was reverse-transcribed into cDNA using a reverse transcription kit. RT–qPCR was performed using SYBR Green Real-time PCR Master Mix (TOYOBO, Shanghai, China) on a real-time quantitative PCR system (ABI 7500). The expression levels of target genes, including Tlr2, Tlr3, Tlr4, Tlr6, Tlr7, Cd36, and Cd14, were analyzed. GAPDH was used as an internal reference gene. Relative gene expression levels were calculated using the $2^{-\Delta\Delta Ct}$ method. All experiments were performed in triplicate ($n = 3$), and data are presented as mean ± SD. The primer sequences are listed in Table S1.

1.6 Analysis of Membrane Receptor Expression in Macrophages after *Staphylococcus aureus* Stimulation

Macrophage stimulation with *Staphylococcus aureus* was performed as described above. After bacterial exposure, cells were further incubated for 12 h or 24 h before downstream analysis. Cells

cultured without bacterial stimulation were used as the 0 h control group. For Western blot analysis, cells were harvested at the indicated time points and lysed using RIPA buffer supplemented with protease inhibitors (RIPA:protease inhibitor = 100:1). Protein concentrations were determined using a BCA assay. Equal amounts of protein were mixed with SDS loading buffer and incubated at 37 °C for 30 min, followed by separation via SDS–PAGE and transfer to PVDF membranes. Membranes were blocked with 5% non-fat milk for 1 h at room temperature, and subsequently incubated overnight at 4 °C with primary antibodies against TLR2, TLR6, or CD36. After washing with TBST, HRP-conjugated secondary antibodies were applied for 1 h at room temperature. Protein bands were visualized using an enhanced chemiluminescence (ECL) detection system and recorded using a gel imaging system. For immunofluorescence staining, cells were washed with pre-cooled PBS, fixed with 4% paraformaldehyde, and blocked before incubation with primary antibodies against TLR2, TLR6, or CD36 overnight at 4 °C. After washing with PBS, appropriate fluorescently labeled secondary antibodies were added and incubated for 1 h in the dark. Cell nuclei were counterstained with DAPI. Fluorescence images were captured using a confocal laser scanning microscope, with identical imaging parameters maintained across groups for quantitative comparison.

1.7 Cell Membrane Isolation

Macrophages were stimulated with *Staphylococcus aureus* as described above and incubated for an additional 24 h prior to membrane extraction. Cells were collected in pre-chilled sterile PBS containing protease inhibitors using a cell scraper. The cell suspension was subjected to three freeze–thaw cycles between −80 °C and 37 °C to facilitate membrane disruption. Subsequently, samples were sonicated using a cell disruptor (100 W, 30 kHz, 30% amplitude, 3 min per sample). The lysate was first centrifuged at 700 g for 10 min at 4 °C to remove unbroken cells and large debris. The supernatant was then ultracentrifuged at 100 000 g for 1 h at 4 °C, yielding a white membrane pellet that was collected and stored at −80 °C. Prior to use, the membrane pellet was resuspended in sterile PBS and membrane protein content was quantified using a BCA assay. For preparation of unstimulated macrophage membranes, the same procedure was followed without bacterial stimulation. Isolation of red blood cell membranes was performed via hypotonic haemolysis[1, 2]. Briefly, fresh whole blood was collected and centrifuged at 200 g for 20 min at 4 °C in the presence of anticoagulant. The erythrocyte pellet was collected and washed three times with

cold PBS. The erythrocytes were then resuspended in hypotonic saline solution and incubated at 4 °C for 25 min to induce hemolysis. The lysate was centrifuged at 12 000 g for 10 min at 4 °C, and the washing process was repeated three times until the supernatant became clear. The resulting membrane pellet was collected and stored at −80 °C until further use.

1.8 Preparation and Characterization of $MoSe_2$@SPMM

$MoSe_2$ were mixed with macrophage membranes derived from *Staphylococcus aureus*-stimulated cells (SPMM) at a 1:1 mass ratio and gently pipetted on ice to ensure homogeneous mixing. The mixture was allowed to stand for 20 min, followed by 30 min of sonication in an ice-water bath. The suspension was subsequently extruded through polycarbonate membranes with pore sizes of 400 nm and 200 nm using a mini-extruder (Avanti Polar Lipids) for 20–30 cycles, enabling the macrophage membrane to coat the $MoSe_2$ nanoparticles and form $MoSe_2$@SPMM nanostructures. The resulting nanoparticles were purified by centrifugation at 15 000 g for 30 min and stored in 10 mM HEPES buffer (150 mM NaCl, pH 7.4) at 4 °C for subsequent experiments.$MoSe_2$ nanoparticles coated with unstimulated macrophage membranes ($MoSe_2$@MM) or red blood cell membranes ($MoSe_2$@RBCM) were prepared using the same procedure. $MoSe_2$@DOPC was prepared using a lipid-coating method. DOPC liposomes were first obtained by thin-film hydration, followed by sonication. $MoSe_2$ nanoparticles were then mixed with DOPC liposomes and subjected to sonication/extrusion to promote lipid coating. The resulting $MoSe_2$@DOPC was collected by centrifugation, washed with PBS to remove excess lipids, and resuspended for subsequent experiments. The hydrodynamic size and zeta potential of nanoparticles were measured by dynamic light scattering using a Malvern Zetasizer Pro analyzer (Malvern Panalytical, Malvern, UK). For stability evaluation, $MoSe_2$@SPMM nanoparticles were dispersed in PBS or FBS-containing solution with a final protein concentration of 1.0 mg mL^{-1}. Particle size was measured in triplicate and monitored continuously at room temperature for 15 days. Nanoparticle morphology was observed using transmission electron microscopy (TEM). Samples were diluted in 10 mM HEPES buffer (150 mM NaCl, pH 7.4) and 10 µL of suspension was deposited onto carbon-coated copper grids. After 5 min adsorption, excess liquid was removed with filter paper. Negative staining was performed using 1% phosphotungstic acid (PTA, pH ≈ 7.0) for 60 s, followed by air drying. TEM images were acquired at 100 kV, and elemental distribution was analyzed using STEM–EDS mapping. Surface elemental composition and chemical states

were further characterized using X-ray photoelectron spectroscopy (XPS, Thermo Scientific K-Alpha). Samples were drop-cast onto silicon wafers and dried prior to measurement. The analysis chamber pressure was maintained below 2.0 × 10^{-7} mbar. The X-ray spot size was 400 μm, with an operating voltage of 12 kV and filament current of 6 mA. Survey spectra were collected with a pass energy of 150 eV, while high-resolution spectra were acquired with a pass energy of 50 eV and step size of 0.1 eV. Binding energies were calibrated using the C 1s peak at 284.8 eV. To confirm membrane protein retention on nanoparticle surfaces, SDS–PAGE analysis was performed for $MoSe_2$, $MoSe_2$@MM, $MoSe_2$@SPMM, and lysates from bacteria-stimulated macrophages. Equal amounts of protein were mixed with 4× loading buffer, denatured at 95 °C for 10 min, and separated on polyacrylamide gels. After electrophoresis, gels were stained with Coomassie Brilliant Blue and imaged.

1.9 Evaluation of Reactive Oxygen Species (ROS) Scavenging Activity

Prior to H_2O_2 addition, dissolved oxygen in deionized water was minimized by heating and vacuum treatment. $MoSe_2$@SPMM (200 μg/mL) was then incubated with H_2O_2 solution (5 × 10^{-4} mol·L^{-1}), and the residual H_2O_2 was quantified every 15 min using a H_2O_2 assay kit according to the manufacturer's instructions. To evaluate O_2 generation during catalytic decomposition, real-time dissolved oxygen levels were recorded at 60 s intervals using a portable dissolved oxygen meter (JPB-607A, Inesa Scientific Instrument CO., LTD., China). For cyclic H_2O_2 decomposition analysis, $MoSe_2$@SPMM was added to H_2O_2 solution (5 × 10^{-4} mol·L^{-1}) and allowed to react for 20 min, followed by centrifugation at 5000 × g for 10 min. The precipitate was collected, re-dispersed in fresh H_2O_2 solution, and subjected to repeated reaction cycles. After each cycle, the residual H_2O_2 concentration in the supernatant was measured to assess the catalytic stability of $MoSe_2$@SPMM. The SOD-like activity was assessed using a kit based on the xanthine–xanthine oxidase system, which generates superoxide anions ($O_2 {\bullet}^-$) capable of reducing nitroblue tetrazolium (NBT). After adding the nanomaterials to the reaction mixture, samples were incubated under the specified conditions. Materials exhibiting SOD-like activity scavenged $O_2 {\bullet}^-$ and inhibited NBT reduction, resulting in decreased color development. Absorbance was measured at 506 nm using a microplate reader, and the inhibition percentage of NBT reduction was calculated. The GPx-like activity of $MoSe_2$@SPMM was evaluated using a GPx activity assay kit (Solarbio, Beijing, China) according to the manufacturer's instructions. This assay is based on a glutathione

reductase (GR)-coupled reaction system. In the presence of H_2O_2, the GPx-like activity catalyzes the oxidation of reduced glutathione (GSH) to oxidized glutathione (GSSG). Subsequently, GSSG is reduced back to GSH by GR, accompanied by the oxidation of NADPH to $NADP^+$. The GPx-like activity was determined by monitoring the decrease in NADPH absorbance at 340 nm. $MoSe_2$-based nanomaterials at different concentrations (0, 50, 100, 200, and 400 μg/mL) were added to the reaction system, and the absorbance was recorded every 30 s over time. The GPx-like activity within 10 min was calculated according to the kit instructions and expressed as U/mL. For the hydroxyl radical scavenging assay, a •OH-generating system was established according to the kit protocol. Nanomaterials were added to the reaction mixture and allowed to react under the recommended conditions. Samples with hydroxyl radical scavenging capability reduced the formation of colored reaction products. Absorbance was recorded at 536 nm.For the DPPH radical scavenging assay, nanomaterials were mixed with DPPH working solution and incubated in the dark for a defined period. As electron donors, the nanomaterials reduced DPPH radicals, resulting in a gradual loss of the characteristic purple color. The absorbance was measured at 515 nm. To eliminate possible interference caused by nanoparticle color or light scattering, each experimental group included corresponding sample blank controls, and background correction was applied during data processing. The ROS-scavenging ability of $MoSe_2$, $MoSe_2$@MM, and $MoSe_2$@SPMM was evaluated using three complementary assays, including a total superoxide dismutase (SOD) activity assay, a hydroxyl radical (•OH) scavenging assay, and a DPPH radical scavenging assay. Nanomaterials were first dispersed in PBS by ultrasonication and adjusted to a final concentration of 200 μg mL^{-1}. Reaction systems without nanomaterials served as negative controls. All assays were conducted strictly according to the manufacturers' instructions, and each experiment was performed in triplicate.

1.10 Electron Paramagnetic Resonance (EPR) Analysis of Radical Scavenging

The scavenging capacity of $MoSe_2$@SPMM toward superoxide anion radicals (•O_2^-) and hydroxyl radicals (•OH) was further examined using electron paramagnetic resonance (EPR) spectroscopy combined with spin-trapping analysis. For detection of •O_2^-, a radical-generating system was established using the xanthine/xanthine oxidase (X/XO) reaction. Xanthine was first mixed with xanthine oxidase to continuously generate •O_2^-. $MoSe_2$@SPMM suspensions at different concentrations (100, 200, and 400 μg mL^{-1}) were then introduced into the reaction system, while

a system without nanomaterials served as the control. After a defined reaction period, 5,5-dimethyl-1-pyrroline N-oxide (DMPO) was added as a spin-trapping agent to capture residual radicals, forming stable DMPO–OOH adducts. For detection of •OH, radicals were generated through a Fenton reaction by mixing Fe^{2+} with H_2O_2 under controlled conditions. $MoSe_2$@SPMM suspensions (100, 200, and 400 μg mL^{-1}) were then added to the system, while the control group contained no nanomaterial. After incubation, DMPO was introduced to trap the remaining hydroxyl radicals, producing DMPO–OH adducts. The total volume of each reaction system was 5 mL. EPR measurements were conducted at room temperature using a Bruker A300 EMXplus spectrometer (Bruker, Germany). Characteristic EPR signals of DMPO–OOH and DMPO–OH adducts were recorded. Radical scavenging performance was evaluated by comparing signal intensities between treated samples and control groups.

1.11 Photothermal Performance Evaluation

$MoSe_2$@SPMM nanoparticles were dispersed in deionized water or PBS and ultrasonicated to obtain homogeneous suspensions. Equal volumes of each sample were transferred into quartz cuvettes or centrifuge tubes, ensuring identical liquid levels among groups. The corresponding solvent served as a blank control. An 808 nm near-infrared (NIR) laser was applied vertically to the center of the liquid surface while maintaining a constant irradiation distance. In the first set of experiments, samples with a fixed concentration were irradiated for 300 s under different laser power densities (0, 0.2, 0.4, 0.6, 0.8, and 1.0 W cm^{-2}), and the temperature change was continuously recorded. In a second set of experiments, the laser power density was kept constant while nanoparticle concentrations were varied (50, 100, 200, and 500 μg mL^{-1}). Temperature–time profiles were recorded to evaluate concentration-dependent photothermal behavior. Temperature changes were monitored in real time using an infrared thermal imaging camera or temperature probe. To examine photothermal stability and reproducibility, three on/off irradiation cycles were performed under representative conditions. Each cycle consisted of 300 s of NIR irradiation followed by natural cooling for 600 s, repeated for three cycles in total. Temperature evolution and peak temperature values were recorded throughout the cycles. Each experiment was conducted at least three times, and averaged values were used for subsequent analysis.

1.12 Targeting Experiments

To visualize the interaction between membrane-coated nanoparticles and bacterial cells, Staphylococcus aureus suspensions (1×10^8 CFU mL^{-1}) were incubated with different nanomaterials. Four experimental groups were included: Control, $MoSe_2$@RBCM, $MoSe_2$@MM, and $MoSe_2$@SPMM. The control group received an equal volume of PBS. Nanomaterials were added at a final concentration of 200 μg mL^{-1} and mixed with the bacterial suspension at a 1:1 volume ratio, followed by incubation at 37 °C for 12 h. After incubation, samples were centrifuged at 5000 rpm for 5 min, and the supernatant was removed. The bacterial pellets were fixed overnight at 4 °C in 4% paraformaldehyde. The fixed samples were then centrifuged again and washed twice with PBS. The resulting suspension was dropped onto silicon wafers (10 μL per sample) and air-dried under a fume hood. Dehydration was carried out through a graded ethanol series (30%, 50%, and 70% for 10 min each, followed by 80%, 90%, 95%, and 100% ethanol for 5 min each). After complete dehydration, samples were allowed to dry naturally and subsequently observed using a scanning electron microscope (SEM, Hitachi S-4800, Japan) to evaluate nanoparticle binding and enrichment on bacterial surfaces.

To examine the bacterial targeting capability of different membrane-coated nanoparticles, $MoSe_2$@RBCM, $MoSe_2$@MM, and $MoSe_2$@SPMM were fluorescently labeled using the lipophilic dye DiO. Briefly, nanoparticle suspensions (200 μg mL^{-1}) were incubated with DiO at 37 °C for 30 min in the dark. Excess dye was removed by centrifugation and washing, and the labeled nanoparticles were resuspended in sterile PBS. Meanwhile, *S. aureus* and *E. coli* suspensions were adjusted to 1×10^8 CFU mL^{-1} and stained with Hoechst 33342 at 37 °C for 15 min in the dark. The bacteria were subsequently washed by centrifugation to remove unbound dye and resuspended in sterile PBS. DiO-labeled nanoparticles were then mixed with Hoechst-stained bacteria at a 1:1 volume ratio and incubated at 37 °C for 60 min in the dark. After incubation, a small aliquot of the suspension was transferred to glass-bottom confocal dishes. Fluorescence images were acquired using a laser scanning confocal microscope (FV1000, Olympus) under the green (DiO) and blue (Hoechst) channels.

To evaluate the cellular uptake of membrane-coated nanoparticles, $MoSe_2$@RBCM, $MoSe_2$@MM, and $MoSe_2$@SPMM were labeled with the fluorescent dye DiI. RAW264.7 macrophages, NIH3T3 fibroblasts, and HUVEC were seeded into glass-bottom confocal culture dishes and cultured at 37 °C in a humidified incubator with 5% CO_2 until reaching approximately

70% confluence. To induce oxidative stress, cells were incubated with 200 μM H_2O_2 for 4 h.DiI-labeled nanoparticles (200 μg mL^{-1}) were then added to the cells and incubated for 24 h. After incubation, the culture medium was removed and cells were gently washed with PBS to eliminate non-internalized nanoparticles. Cellular uptake and distribution of nanoparticles were examined using a laser scanning confocal microscope, and fluorescence images were recorded.

1.13 In Vitro Antibacterial Evaluation

The antibacterial activity of different nanomaterials against Staphylococcus aureus was evaluated using a plate counting assay and a crystal violet biofilm assay, both performed with or without near-infrared (NIR) irradiation. Bacterial suspensions were prepared at 1×10^7 CFU mL^{-1} and mixed with different nanomaterial dispersions. Four experimental groups were established: Control, $MoSe_2$@RBCM, $MoSe_2$@MM, and $MoSe_2$@SPMM, each with a final material concentration of 200 μg mL^{-1}. After mixing, the NIR^+ groups were exposed to 808 nm NIR laser irradiation (1 W cm^{-2}) for 10 min, whereas the NIR^- groups were kept under identical conditions without irradiation. The mixtures were then incubated at 37 °C for 12 h.

For bacterial colony counting, the bacterial suspensions were serially diluted with sterile PBS to 1×10^4 CFU mL^{-1}. An aliquot of 200 μL from each dilution was evenly spread onto LB agar plates and incubated overnight at 37 °C. Colony-forming units (CFUs) were counted and compared among groups to evaluate antibacterial efficacy.

For biofilm formation analysis, the bacteria–material mixtures were transferred into 24-well plates and cultured under the same NIR irradiation conditions described above. After incubation at 37 °C for 48 h, the supernatant was removed and the wells were gently washed with PBS to eliminate planktonic bacteria. Biofilms were stained with 0.1% crystal violet solution for 15 min, followed by thorough PBS washing until the wash solution became clear. The bound dye was then dissolved with 95% ethanol, and the absorbance at 550 nm was measured using a microplate reader to quantify biofilm biomass.

To visualize bacterial viability after treatment, live/dead staining was performed on planktonic bacteria. After co-incubation with nanomaterials, bacterial suspensions were centrifuged at 5000 rpm for 3 min, and the pellets were washed three times with PBS. A 100 μL bacterial suspension was transferred into a new microcentrifuge tube and mixed with 1 μL of 100×

DMAO/PI staining solution. After incubation for 15 min in the dark, 10 μL of the stained suspension was dropped onto a glass slide and covered with a coverslip. Fluorescence images were acquired using a laser scanning confocal microscope (FV1000, Olympus). For visualization of biofilm viability, bacterial suspensions (1×10^7 CFU mL^{-1}) were added to glass-bottom confocal dishes (1 mL per dish) together with the corresponding nanomaterial treatments. The cultures were incubated at 37 °C for 48 h to allow biofilm formation. Subsequently, DMAO/PI staining solution (150 μL per dish) was added and incubated for 15 min in the dark. After staining, the dye solution was removed and the samples were gently rinsed with PBS. Biofilms were observed using a laser scanning confocal microscope (FV1000, Olympus). To evaluate biofilm architecture, z-stack scanning was performed during confocal imaging. The resulting optical sections were reconstructed into three-dimensional biofilm structures, and biofilm thickness was quantified based on the reconstructed images.

1.14 Evaluation of Oxidative Stress Mitigation and Macrophage Reprogramming

RAW264.7 macrophages were cultured in DMEM supplemented with 10% fetal bovine serum and 1% penicillin–streptomycin at 37 °C in a humidified incubator containing 5% CO_2. When the cells reached approximately 70% confluence, the culture medium was removed and the cells were washed once with PBS. To induce oxidative stress, cells were incubated with 200 μM H_2O_2 for 4 h. Cells cultured under identical conditions without H_2O_2 treatment served as the control group. For the oxidative stress model, a positive control group received H_2O_2 treatment without nanomaterial intervention, whereas the experimental groups were treated with $MoSe_2$@RBCM, $MoSe_2$@MM, or $MoSe_2$@SPMM at a final concentration of 200 μg mL^{-1} following H_2O_2 stimulation.

Intracellular reactive oxygen species (ROS) levels were determined using a DCFH-DA–based ROS detection kit. After the designated treatments, cells were gently washed with PBS and incubated with 10 μM DCFH-DA working solution at 37 °C for 30 min in the dark. Excess probe that had not entered the cells was removed by PBS washing.Fluorescence images were acquired using a confocal laser scanning microscope (FV1000, Olympus). The fluorescence intensity of each group was quantified using ImageJ software to evaluate intracellular ROS levels.

Changes in mitochondrial membrane potential ($\Delta\Psi m$) were assessed using a JC-1 mitochondrial membrane potential assay kit. After treatment, cells were incubated with 2 μM JC-

1 staining solution at 37 °C for 30 min in the dark, followed by washing with JC-1 buffer. Fluorescence images were obtained using a confocal microscope (FV1000, Olympus). Under high mitochondrial membrane potential, JC-1 forms aggregates that emit red fluorescence, whereas depolarized mitochondria exhibit green fluorescence due to the monomeric form of JC-1. The ratio of red to green fluorescence intensity was calculated to quantify changes in mitochondrial membrane potential.

To assess macrophage phenotypic reprogramming, cells were fixed with ice-cold methanol for 10 min after treatment and subsequently blocked with 5% bovine serum albumin (BSA) for 1 h at room temperature. Cells were then incubated overnight at 4 °C with primary antibodies against iNOS (M1 marker) and Arg-1 (M2 marker) at a dilution of 1:200. After washing with PBS, cells were incubated with the corresponding fluorescent secondary antibodies (1:500) for 1 h at room temperature in the dark. Cell nuclei were counterstained using DAPI-containing antifade mounting medium. Fluorescence images were collected using a laser scanning confocal microscope (FV1000, Olympus). Quantitative analysis of fluorescence intensity at the single-cell level was performed using ImageJ, allowing comparison of macrophage polarization states among different treatment groups.

The secretion levels of inflammatory cytokines produced by RAW264.7 macrophages under different treatment conditions were measured using enzyme-linked immunosorbent assay (ELISA) kits according to the manufacturers' instructions.Specifically, the concentrations of tumor necrosis factor-α (TNF-α), interleukin-6 (IL-6), interleukin-10 (IL-10), and transforming growth factor-β (TGF-β) were quantified. Briefly, standards and samples were added to antibody-coated microplates and incubated, followed by sequential addition of detection antibodies and enzyme conjugates. After incubation with chromogenic substrate, the reaction was terminated and absorbance was measured at 450 nm using a microplate reader. Cytokine concentrations were calculated based on standard calibration curves.

1.15 Evaluation of Macrophage-Mediated Regulation of Vascular Endothelial Cell Function

Oxidative stress induction and material treatment of macrophages were performed as described above. After treatment, the culture supernatants from each group were collected and centrifuged at 3000 rpm for 10 min to remove cellular debris. The resulting supernatants were used as macrophage-conditioned medium (CM) and temporarily stored at 4 °C before use in endothelial

cell assays. HUVECs were seeded in multi-well plates or glass-bottom confocal dishes and cultured in endothelial cell growth medium supplemented with 10% fetal bovine serum at 37 °C in a humidified atmosphere containing 5% CO_2. When the cells reached approximately 70–80% confluence, oxidative stress was induced by replacing the culture medium with H_2O_2-containing medium. The medium was subsequently replaced with macrophage-derived CM from the different treatment groups to evaluate the regulatory effect of macrophage secretions on endothelial cell function.

Cell proliferation was assessed using an EdU incorporation assay. After HUVECs were cultured with CCM from different groups for 24 h, EdU (10 μM) was added according to the kit protocol and cells were incubated for an additional 4 h. The culture medium was then removed and cells were fixed with 4% paraformaldehyde for 15 min. Following PBS washing, cells were permeabilized using 0.3% Triton X-100, and 200 μL Click reaction solution was added to each well for 30 min incubation in the dark at room temperature. Finally, nuclei were counterstained using DAPI-containing antifade reagent. Fluorescence images were captured using a confocal laser scanning microscope (FV1000, Olympus). The percentage of EdU-positive cells was calculated to quantify endothelial cell proliferation.

HUVECs were seeded in 6-well plates and cultured with macrophage-derived CM until a confluent monolayer formed. A sterile pipette tip was then used to generate a linear scratch wound across the cell monolayer. Detached cells were removed by gentle PBS washing, and fresh CM from the corresponding treatment groups was added. Images of the scratch area were recorded at 0 h and 36 h. The wound closure area was quantified using ImageJ software, and the migration capacity of endothelial cells was evaluated based on the percentage of wound closure.

The migration of HUVECs was further examined using a Transwell chamber system. After exposure to CCM from different groups for 24 h, HUVECs were resuspended in serum-free medium and seeded into the upper chamber at 5×10^4 cells in 200 μL per well. The lower chamber was filled with 500 μL complete medium containing 10% fetal bovine serum. The Transwell plates were incubated at 37 °C with 5% CO_2 for 12 h to allow cell migration. After incubation, the inserts were removed and gently washed with PBS. Cells remaining on the upper surface of the membrane were carefully removed using a cotton swab. Migrated cells on the lower surface were fixed with 4% paraformaldehyde for 15 min and stained with 0.1% crystal violet for 15 min. After washing

with PBS to remove excess dye, the migrated cells were observed and photographed using an inverted microscope. Multiple random fields were imaged for each sample, and the number of migrated cells was quantified for statistical analysis.

To assess the angiogenic capacity of endothelial cells, a Matrigel tube formation assay was performed. HUVECs were first pretreated with macrophage-derived CCM for 24 h. Cells were then harvested and seeded into Matrigel-coated 24-well plates, followed by incubation with the corresponding CCM. After 6 h of incubation, tube-like structures were observed under a microscope and images were captured. The extent of tube formation was quantified using ImageJ, including parameters such as branch formation and network structure.

The expression of angiogenesis-related proteins in HUVECs was analyzed by immunofluorescence staining. After treatment with CM for 24 h, cells cultured in confocal dishes were fixed with 4% paraformaldehyde for 15 min, permeabilized with 0.1% Triton X-100 for 10 min, and blocked with 5% BSA for 1 h. Cells were then incubated overnight at 4 °C with primary antibodies against VEGF and bFGF (1:200). After washing with PBS, fluorescent secondary antibodies (1:500) were applied and incubated for 1 h at room temperature in the dark. Following PBS washing, nuclei were stained with DAPI-containing antifade reagent. Fluorescence images were obtained using a laser scanning confocal microscope (FV1000, Olympus). The fluorescence intensity of VEGF and bFGF at the single-cell level was quantified using ImageJ software to evaluate angiogenic protein expression.

Macrophage oxidative stress induction and material treatments were performed as described above. After treatment, culture supernatants from each group were collected and centrifuged at 3000 rpm for 10 min to remove cellular debris. The resulting CCM was stored at 4 °C for short-term use in subsequent NIH3T3 fibroblast assays. Fibroblast were seeded in multiwell plates or confocal dishes and cultured in DMEM supplemented with 10% fetal bovine serum at 37 °C in a humidified atmosphere with 5% CO_2 until reaching 70–80% confluence. Oxidative stress was then induced by replacing the medium with H_2O_2-containing medium, followed by incubation with CCM derived from differently treated macrophage groups for 24 h to evaluate the regulatory effects of macrophage-derived CCM on fibroblast function.

For scratch assay, fibroblasts were seeded in 6-well plates and cultured with CCM from different groups until reaching full confluence. A linear scratch was created across the cell

monolayer using a sterile pipette tip. The cells were gently washed with PBS to remove detached cells and then incubated with serum-free CCM from the corresponding groups. Images of the wound area were captured at 0 and 24 h, and the wound closure area was quantified using ImageJ to calculate the cell migration rate.

For Transwell migration assay, cells were pretreated with CCM from different groups for 24 h, then harvested and resuspended in serum-free medium. A total of 5×10^4 cells in 200 μL were seeded into the upper chamber, while 500 μL of complete medium containing 10% fetal bovine serum was added to the lower chamber. The inserts were incubated at 37 °C in a 5% CO_2 atmosphere for 12 h. After incubation, non-migrated cells on the upper surface were gently removed with a cotton swab, and the migrated cells on the lower surface were fixed with 4% paraformaldehyde for 15 min and stained with 0.1% crystal violet for 15 min. The inserts were washed with PBS to remove excess dye, and migrated cells were imaged under an inverted microscope. Multiple random fields were captured for each sample and used for quantitative analysis of cell migration.

1.16 Immunofluorescence Analysis

Immunofluorescence staining was performed to assess the expression of cell adhesion– and extracellular matrix–related proteins in fibroblasts. Cells were seeded in glass bottom culture dishes and cultured with CCM from different treatment groups for 24 h. The cells were then fixed with 4% paraformaldehyde for 15 min, permeabilized with 0.1% Triton X-100 for 10 min, and blocked with 5% BSA for 1 h. Primary antibodies against vinculin, fibronectin (FN-1), collagen I, and collagen III (1:200) were applied and incubated overnight at 4 °C. After washing with PBS, cells were incubated with fluorophore-conjugated secondary antibodies (1:500) for 1 h at room temperature in the dark. Actin filaments were subsequently stained with AF488-labeled phalloidin (1:100) for 30 min, followed by nuclear staining with DAPI using an antifade mounting medium. Fluorescence images were acquired using a confocal laser scanning microscope (FV1000, Olympus), and the fluorescence intensity at the single-cell level was quantified using ImageJ.

1.17 In vivo study

All animals were obtained from the Experimental Animal Center of Air Force Medical University. All procedures were approved by the Institutional Animal Care and Use Committee of Fourth

Military Medical University (Approval No. kq-2025-166) and conducted in accordance with relevant guidelines. Male Sprague–Dawley rats (4–6 weeks old) were housed under specific pathogen-free (SPF) conditions.

After one week of acclimatization, bilateral maxillary first molars were extracted under anesthesia with 3% sodium pentobarbital. The animals were then maintained for 4 weeks to allow socket healing. Subsequently, custom-made implants (diameter: 1.2 mm; length: 4 mm) were placed at the original extraction sites. A suspension of *S. aureus* (20 µL, 10^6 CFU) was injected into the peri-implant soft tissue to establish infection. Starting from the day after implantation, 20 µL of material suspension (200 µg/mL) was locally injected into the peri-implant soft tissue once daily for 3 consecutive days, followed by 808 nm NIR irradiation (1 W/cm^2, 10 min). The control group received no treatment. Animals were sacrificed on days 4, 7, and 28 for subsequent analyses. For in vivo antibacterial evaluation (day 4), animals were divided into the following groups: Control, Control + NIR, $MoSe_2$@RBCM, $MoSe_2$@RBCM + NIR, $MoSe_2$@MM, $MoSe_2$@MM + NIR, $MoSe_2$@SPMM, and $MoSe_2$@SPMM + NIR. For immunological (day 7) and STI (day 28) assessments, four groups were included: Control, $MoSe_2$@RBCM, $MoSe_2$@MM, and $MoSe_2$@SPMM, with all treatment groups receiving combined material injection and NIR irradiation for 3 consecutive days. After euthanasia, peri-implant soft tissues were excised, weighed, and homogenized in sterile PBS. The homogenates were mixed with an equal volume of LB medium and incubated at 37 °C for 6 h. The suspensions were then diluted 1000-fold, and 200 µL aliquots were spread onto LB agar plates and incubated overnight at 37 °C. Colony-forming units (CFUs) were counted, and the antibacterial rate was calculated as follows:

$$\text{Antibacterial rate (\%)} = (CFU_{control} - CFU_{experiment}) / CFU_{control} \times 100\%$$

For evaluation of macrophage reprogramming in vivo, rat maxillae were harvested and fixed in 4% paraformaldehyde for 48 h, followed by overnight rinsing in running water. Samples were then decalcified, and the implants were carefully removed after decalcification. The tissues were dehydrated through a graded ethanol series, cleared in xylene, and embedded in paraffin. Serial sections (4–5 µm) were prepared for subsequent analyses. For immunofluorescence staining, sections were permeabilized with 0.025% Triton X-100 in TBS and blocked with TBS containing 10% serum and 1% bovine serum albumin for 2 h at room temperature. Sections were then incubated overnight at 4 °C with primary antibodies against F4/80, iNOS and Arg-1, as well as α-

SMA and integrin. After washing with TBS containing 0.025% Triton X-100, sections were incubated with the corresponding fluorescent secondary antibodies. Nuclei were counterstained with DAPI, and images were acquired using a confocal laser scanning microscope. Hematoxylin and eosin (H&E) staining was performed to assess overall tissue morphology and inflammatory response. Paraffin sections were deparaffinized, rehydrated, and stained according to standard protocols. Masson's trichrome staining was used to evaluate collagen deposition, with collagen fibers stained blue and other tissue components red. Van Gieson (VG) staining was further conducted to assess collagen distribution, where collagen fibers appeared red and background tissue yellow. Collagen deposition and density were quantified using ImageJ.

1.18 Biocompatibility evaluation

The cytocompatibility of $MoSe_2$@SPMM was assessed in RAW264.7 macrophages, L929 fibroblasts, and HUVECs using CCK-8 assays and live/dead staining. Cells were seeded in 96-well plates and cultured to 70–80% confluence at 37 °C under 5% CO_2. The medium was then replaced with fresh complete medium containing $MoSe_2$@SPMM at final concentrations of 6.25–500 μg/mL and incubated overnight. For viability assessment, CCK-8 reagent was added according to the manufacturer's instructions and incubated for 30 min at 37 °C, followed by measurement of absorbance at 450 nm. Cell viability was normalized to untreated controls. For live/dead staining, cells were washed with PBS after treatment and incubated with Calcein-AM/PI working solution for 30 min at 37 °C in the dark. Fluorescence images were acquired using a fluorescence microscope. Hemocompatibility was evaluated by a hemolysis assay using mouse red blood cells. Fresh whole blood was collected with anticoagulant and centrifuged to isolate RBCs, which were washed with PBS until the supernatant became clear and then resuspended to a 4% suspension. $MoSe_2$@SPMM at different concentrations was incubated with the RBC suspension at 37 °C. Physiological saline and deionized water were used as negative and positive controls, respectively. After incubation, samples were centrifuged, and the absorbance of the supernatant was measured at 540 nm. The hemolysis ratio was calculated as:

$$\text{Hemolysis (\%)} = (A_{sample} - A_{negative}) / (A_{positive} - A_{negative}) \times 100\%$$

Systemic biosafety was further evaluated at 4 weeks in the in vivo study. Blood samples were collected via cardiac puncture after euthanasia. Part of the blood was placed in EDTA-containing tubes for hematological analysis, while the remaining blood was allowed to clot and centrifuged

to obtain serum for biochemical assays. Major organs (heart, liver, spleen, lung, and kidney) were harvested, fixed in 4% paraformaldehyde, and processed by standard dehydration, paraffin embedding, and sectioning. Sections were stained with H&E and examined under an optical microscope to assess histopathological changes.

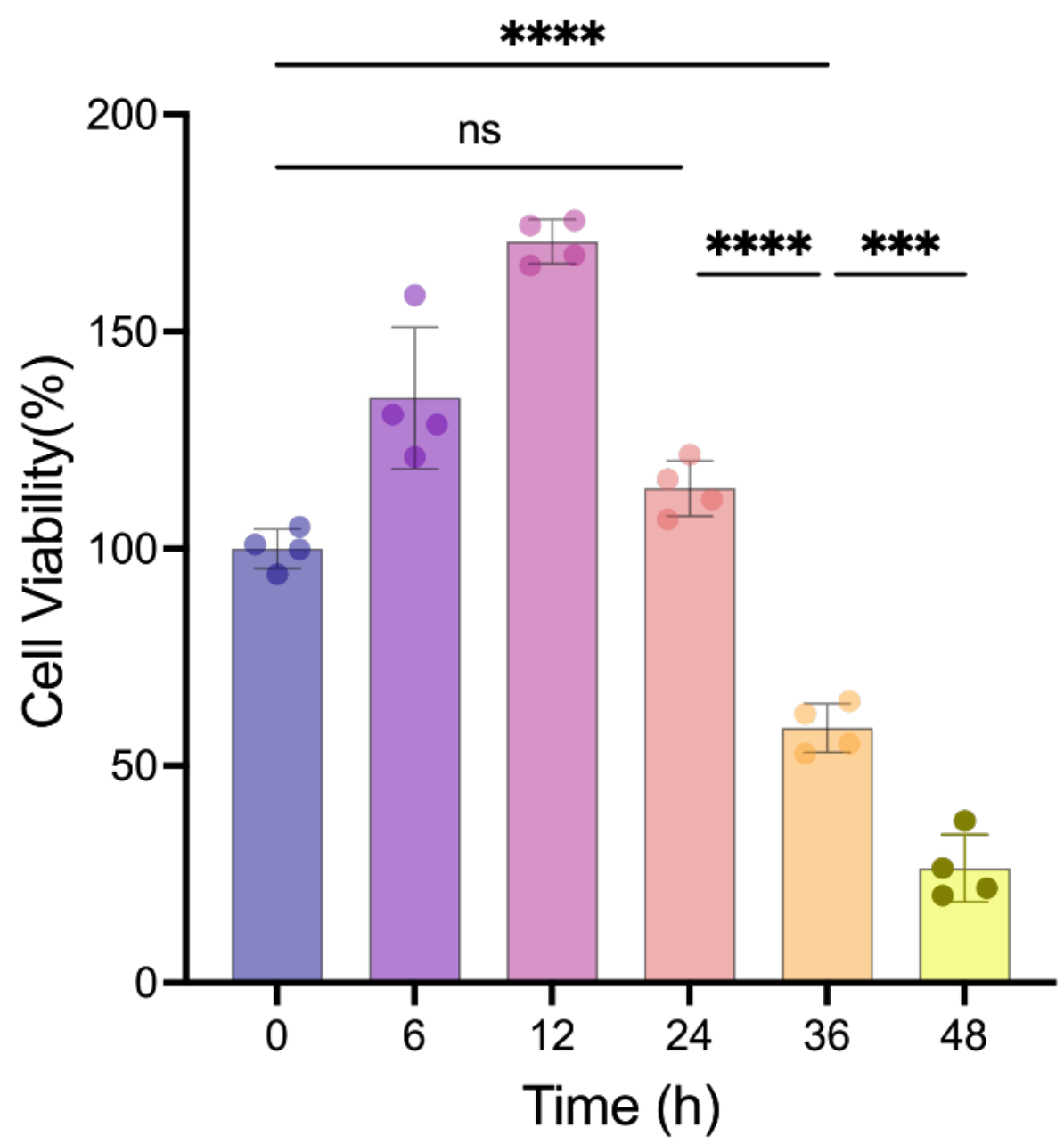

Figure S1 | Effect of *S. aureus* pre-stimulation duration on the viability of RAW264.7 macrophages. RAW264.7 macrophages were exposed to *S. aureus* at a multiplicity of infection (MOI) of 10 for 4 h. The bacterial suspension was then removed and replaced with antibiotic-containing complete medium to terminate the infection, and the cells were further incubated. Cell viability was assessed at different time points (0, 6, 12, 24, 36, and 48 h) using a CCK-8 assay to determine the influence of pre-stimulation duration on macrophage viability. Data are presented as mean ± s.d. (n = 4 biological replicates). Statistical analysis was performed using one-way ANOVA. ns, not significant. ***, $p < 0.001$. ****, $p < 0.0001$.

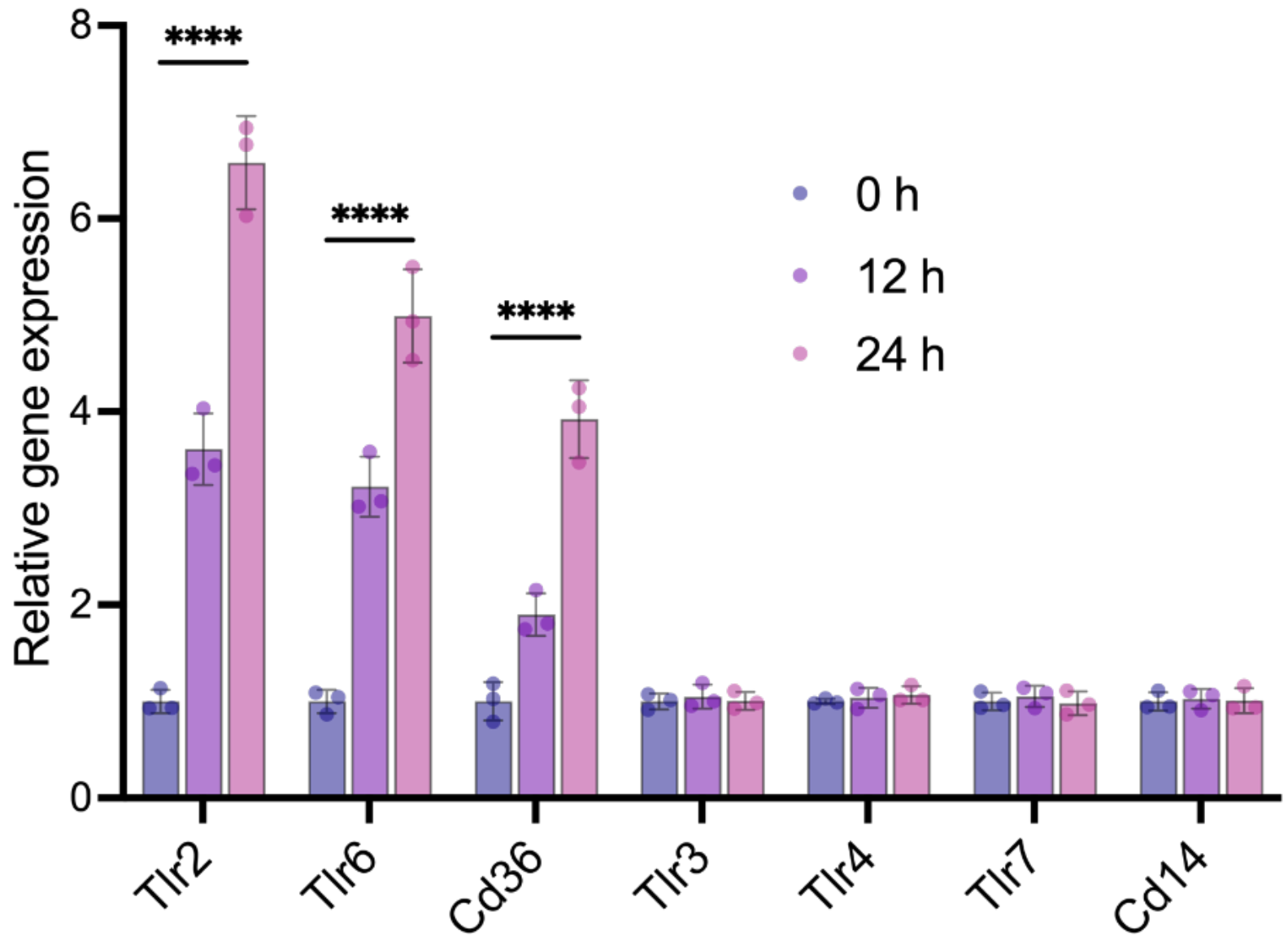

Figure S2 |Relative mRNA expression levels in macrophages at 0, 12, and 24 h after *S. aureus* stimulation were analyzed by RT–qPCR. Tlr2, Tlr6, and Cd36 showed significant upregulation over time, whereas no obvious changes were observed in Tlr3, Tlr4, Tlr7, and Cd14. Data are presented as mean ± s.d. (n = 3 biological replicates). Statistical analysis was performed using one-way ANOVA. ****, $p < 0.0001$.

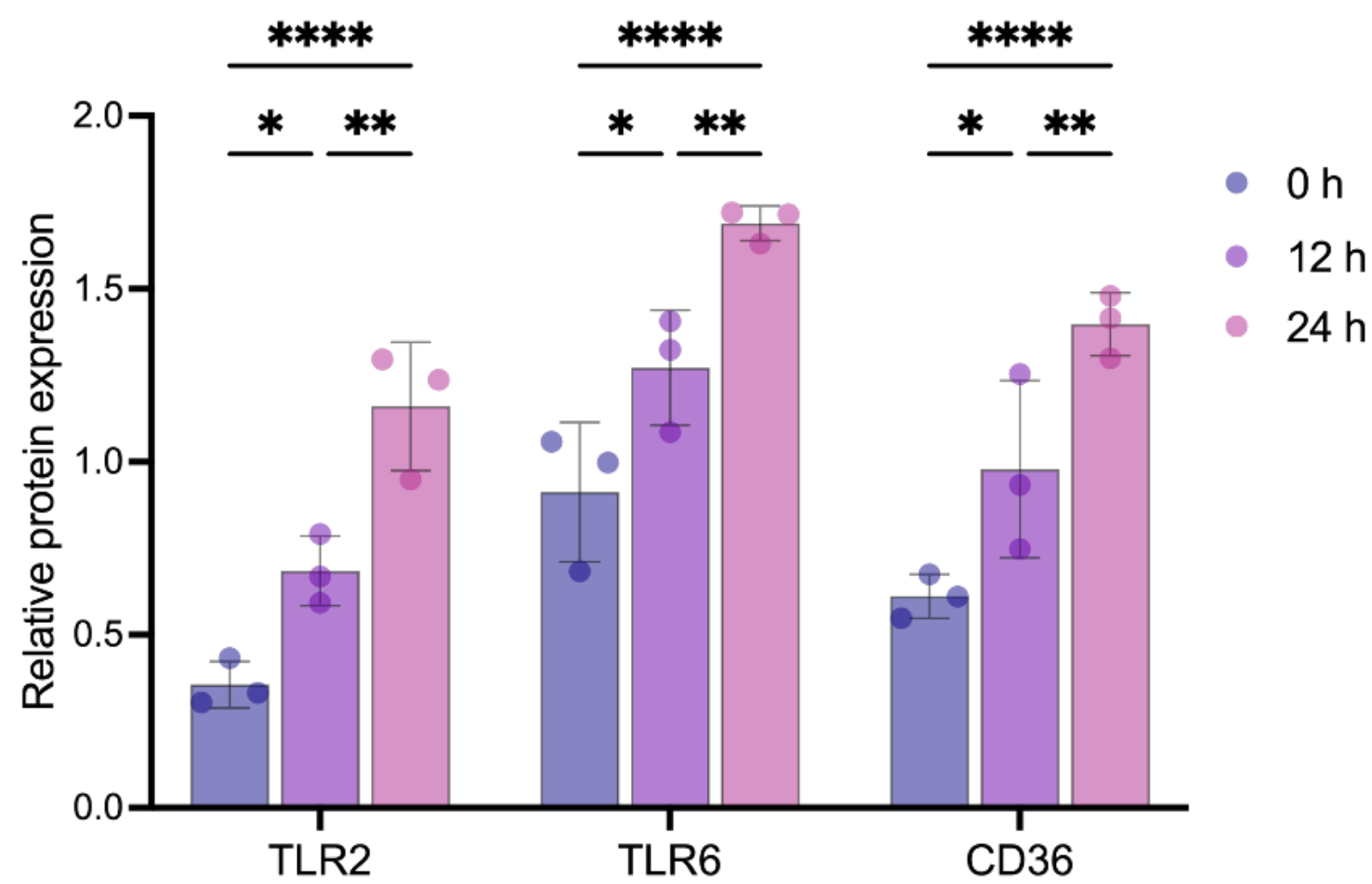

Figure S3 | Quantitative analysis of TLR2, TLR6, and CD36 protein expression in macrophages upon *S. aureus* stimulation. Relative protein expression levels of TLR2, TLR6, and CD36 were quantified by densitometric analysis of Western blot bands. Data are presented as mean ± s.d. (n = 3 biological replicates). Statistical analysis was performed using one-way ANOVA. *, $p < 0.05$. **, $p < 0.01$. ***, $p < 0.01$.

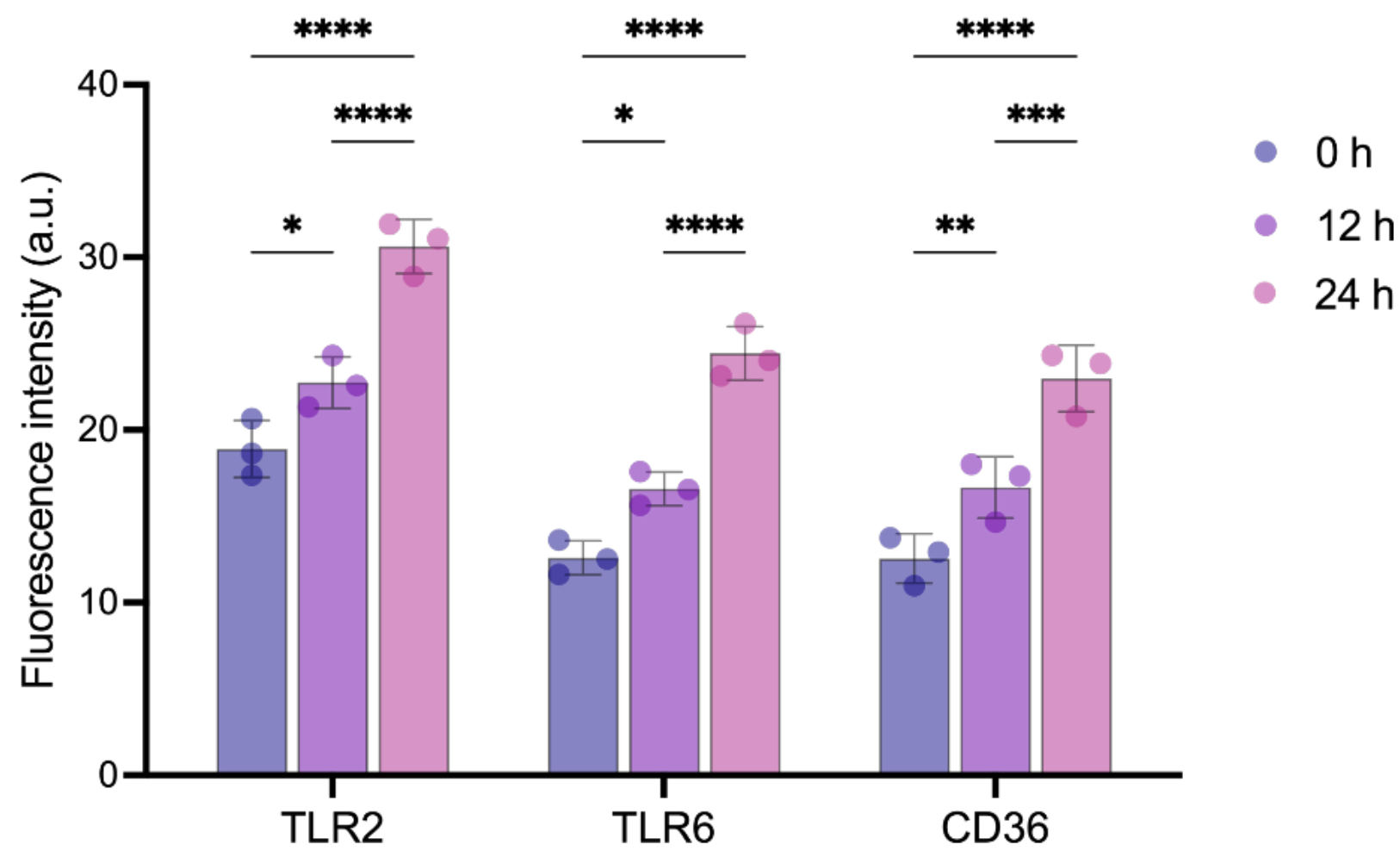

Figure S4 |Quantitative analysis of immunofluorescence intensity of TLR2, TLR6, and CD36 in macrophages at different time points following *S. aureus* stimulation. The mean fluorescence intensity of TLR2, TLR6, and CD36 was quantified from immunofluorescence images to assess their expression changes at different stimulation time points (0, 12, and 24 h). Data are presented as mean ± s.d. (n = 3 biological replicates). Statistical analysis was performed using one-way ANOVA. *, $p < 0.05$. **, $p < 0.01$. ***, $p < 0.001$. ****, $p < 0.0001$.

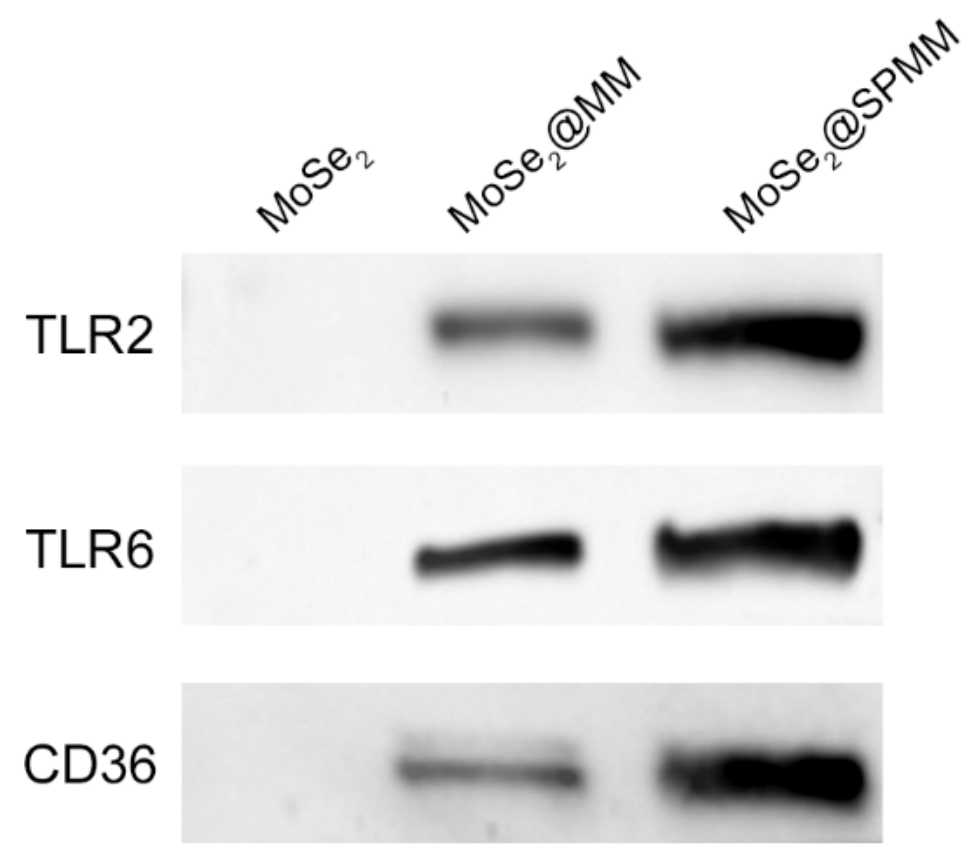

Figure S5 | Western blotting analysis of TLR2, TLR6 and CD36 in the $MoSe_2$, $MoSe_2$@MM, and $MoSe_2$@SPMM. Experiments were repeated independently three times with similar results and a representative result is shown.

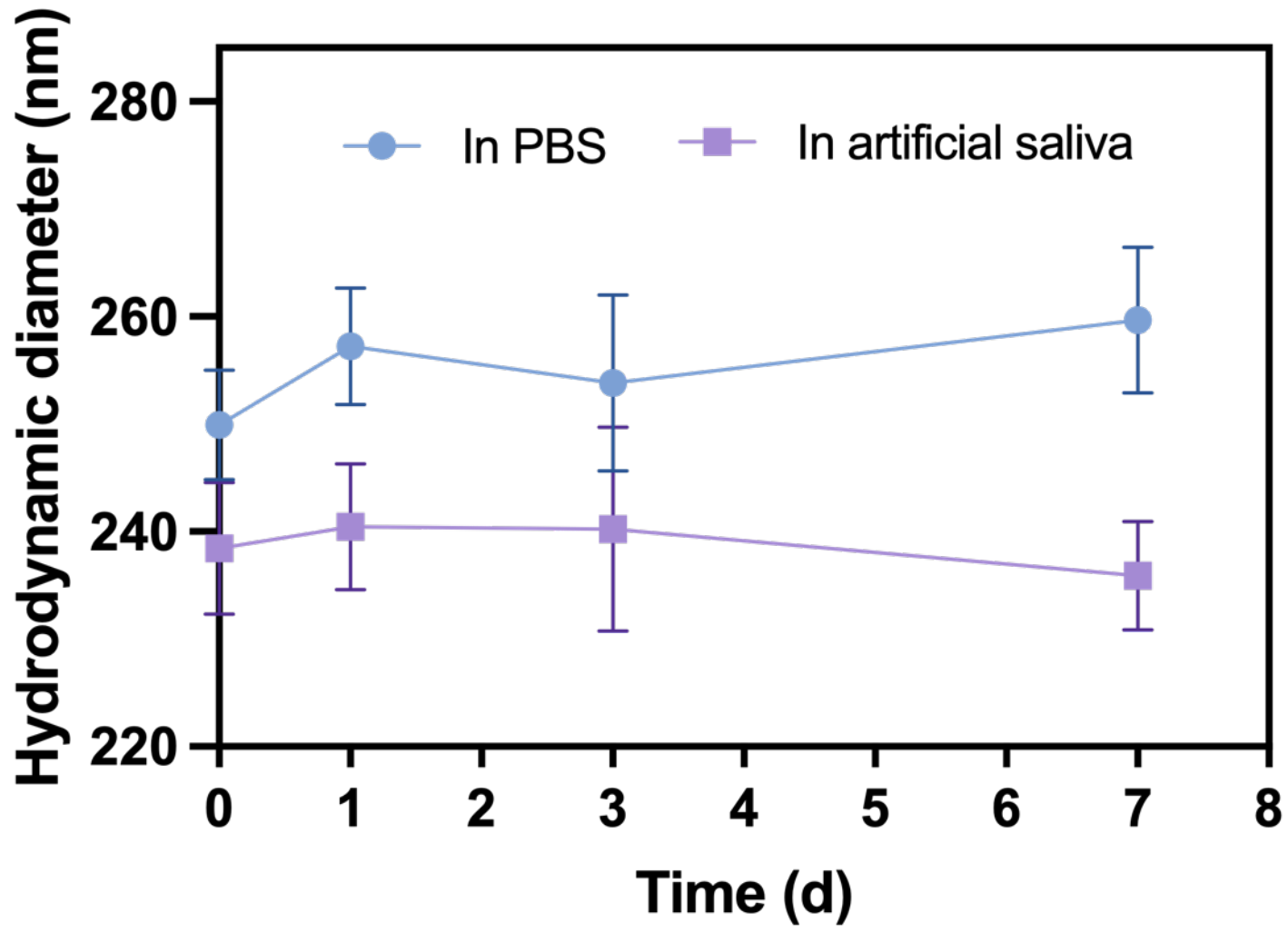

Figure S6 | Stability assessment of $MoSe_2$@SPMM in physiological media. Dynamic light scattering analysis of the hydrodynamic diameter of $MoSe_2$@SPMM dispersed in PBS and artificial saliva over 7 days. The negligible changes in particle size indicate good colloidal stability under physiologically relevant conditions. Data are presented as mean ± s.d. (n = 3).

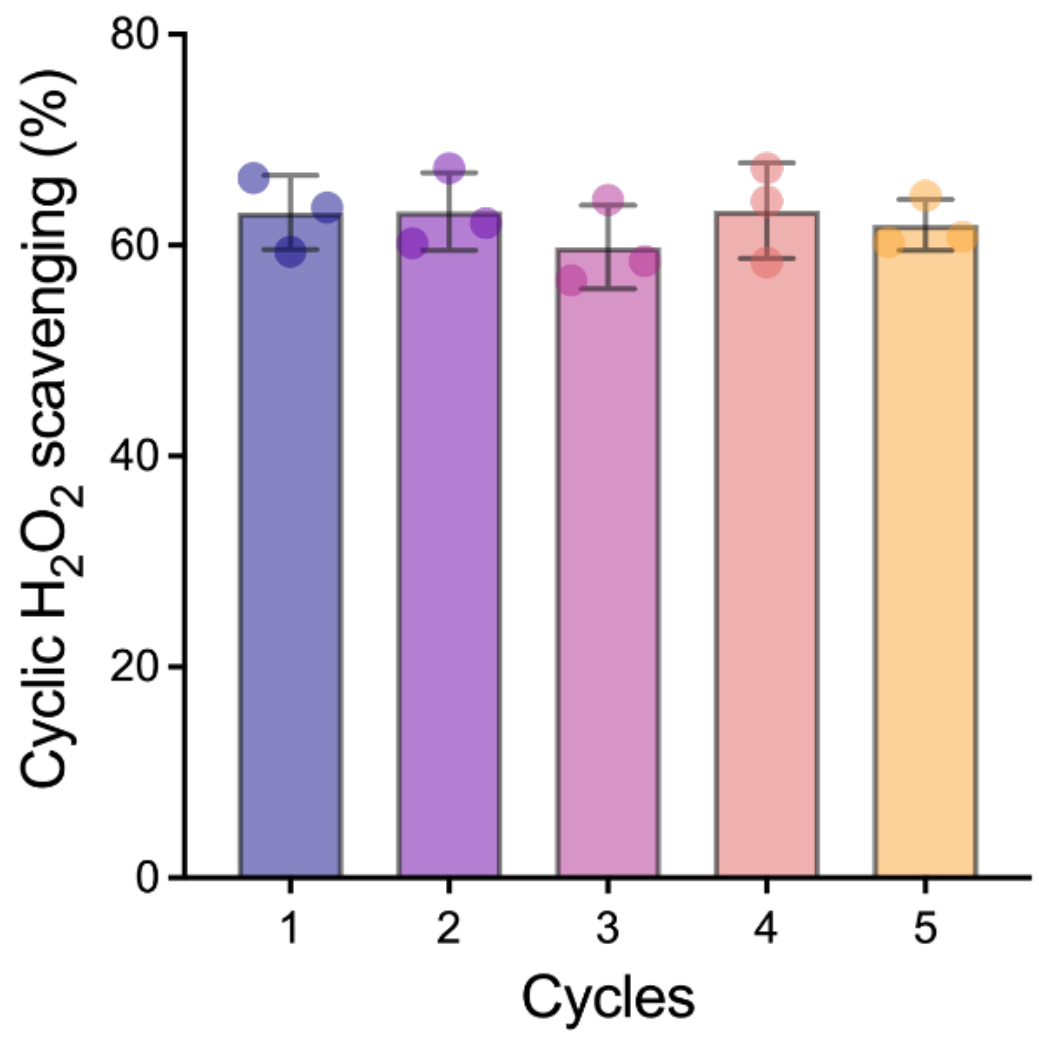

Figure S7 | Cyclic H_2O_2 decomposition performance of $MoSe_2$@SPMM. The scavenging efficiency toward H_2O_2 was assessed over five successive cycles with intermediate centrifugation and re-dispersion. Data are presented as mean ± s.d. (n = 3 biological replicates).

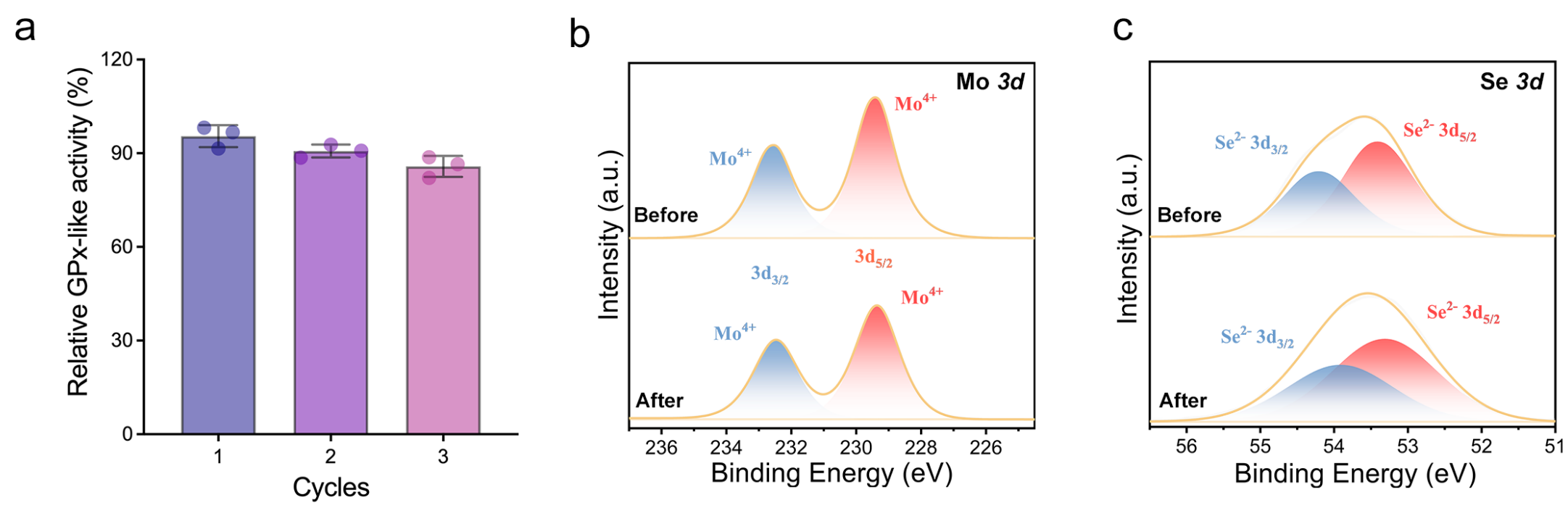

Figure S8 | Catalytic recyclability and chemical stability of $MoSe_2$@SPMM during GPx-like reactions. **(a)** Relative GPx-like activity of $MoSe_2$@SPMM after three consecutive catalytic cycles. The activity in the first cycle was normalized to 100%. XPS spectra of Se 3d **(b)** and Mo 3d **(c)** before and after catalytic cycling. Data are presented as mean ± s.d. (n = 3).

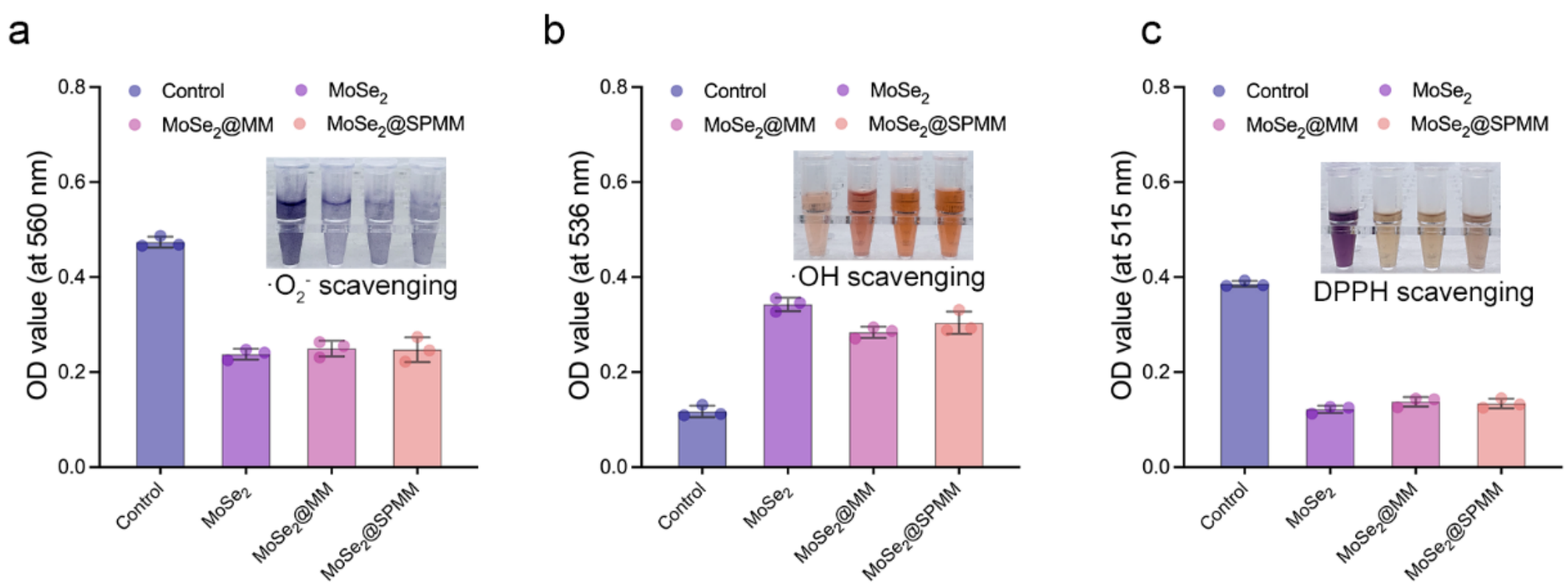

Figure S9 | Evaluation of ROS-scavenging of MoSe₂-based nanomaterials. (a) Superoxide radical ($\cdot O_2^-$) scavenging activity assessed by the nitroblue tetrazolium (NBT) reduction assay. A decrease in absorbance at 560 nm indicates inhibition of NBT reduction and effective superoxide scavenging. **(b)** Hydroxyl radical ($\cdot OH$) scavenging activity was evaluated using a Fenton reaction system.$\cdot OH$ is generated from H_2O_2 in the presence of Fe^{2+} and subsequently oxidizes the o-phenanthroline–Fe^{2+} complex to Fe^{3+}, resulting in a decrease in absorbance at 536 nm. Scavenging of $\cdot OH$ inhibits this process, and the retention of absorbance at 536 nm reflects the $\cdot OH$ scavenging capacity. **(c)** DPPH radical scavenging activity was evaluated by monitoring the decrease in absorbance at 515 nm. The reduction of the characteristic purple DPPH solution reflects the radical-scavenging capability of the samples. Data are presented as mean ± s.d. (n = 3 biological replicates).

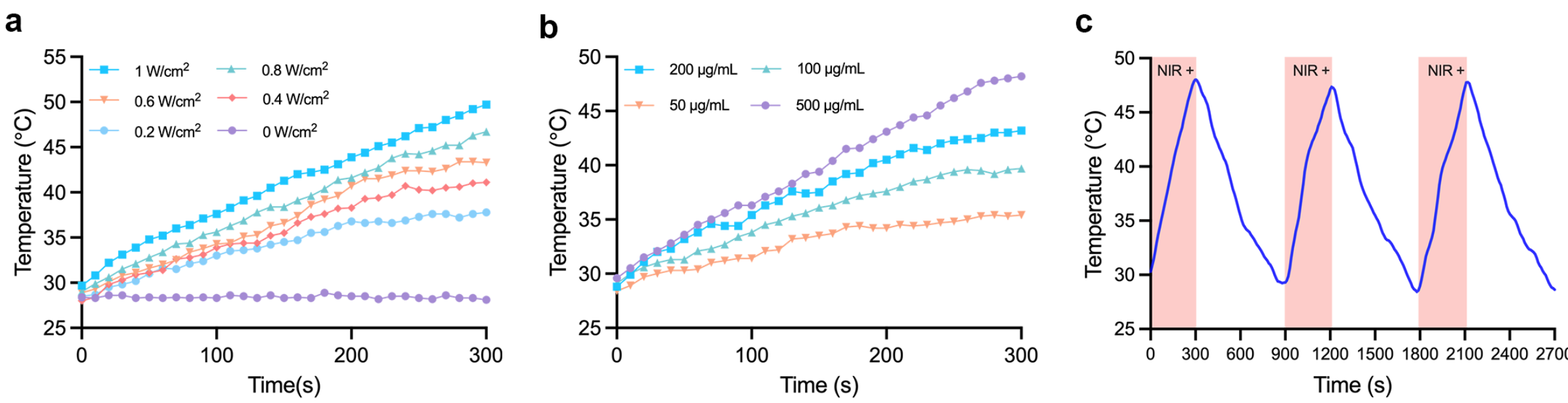

Figure S10 | Photothermal performance of $MoSe_2$@SPMM under 808 nm NIR irradiation. **(a)** Temperature profiles of $MoSe_2$@SPMM dispersions under continuous 808 nm laser irradiation at different power densities (0, 0.2, 0.4, 0.6, 0.8 and 1.0 W cm^{-2}). **(b)** Temperature evolution of $MoSe_2$@SPMM dispersions at various concentrations (50, 100, 200, and 500 μg mL^{-1}) under 808 nm NIR irradiation. **(c)** Photothermal stability of $MoSe_2$@SPMM evaluated over three on/off irradiation cycles (300 s irradiation followed by 600 s cooling per cycle).

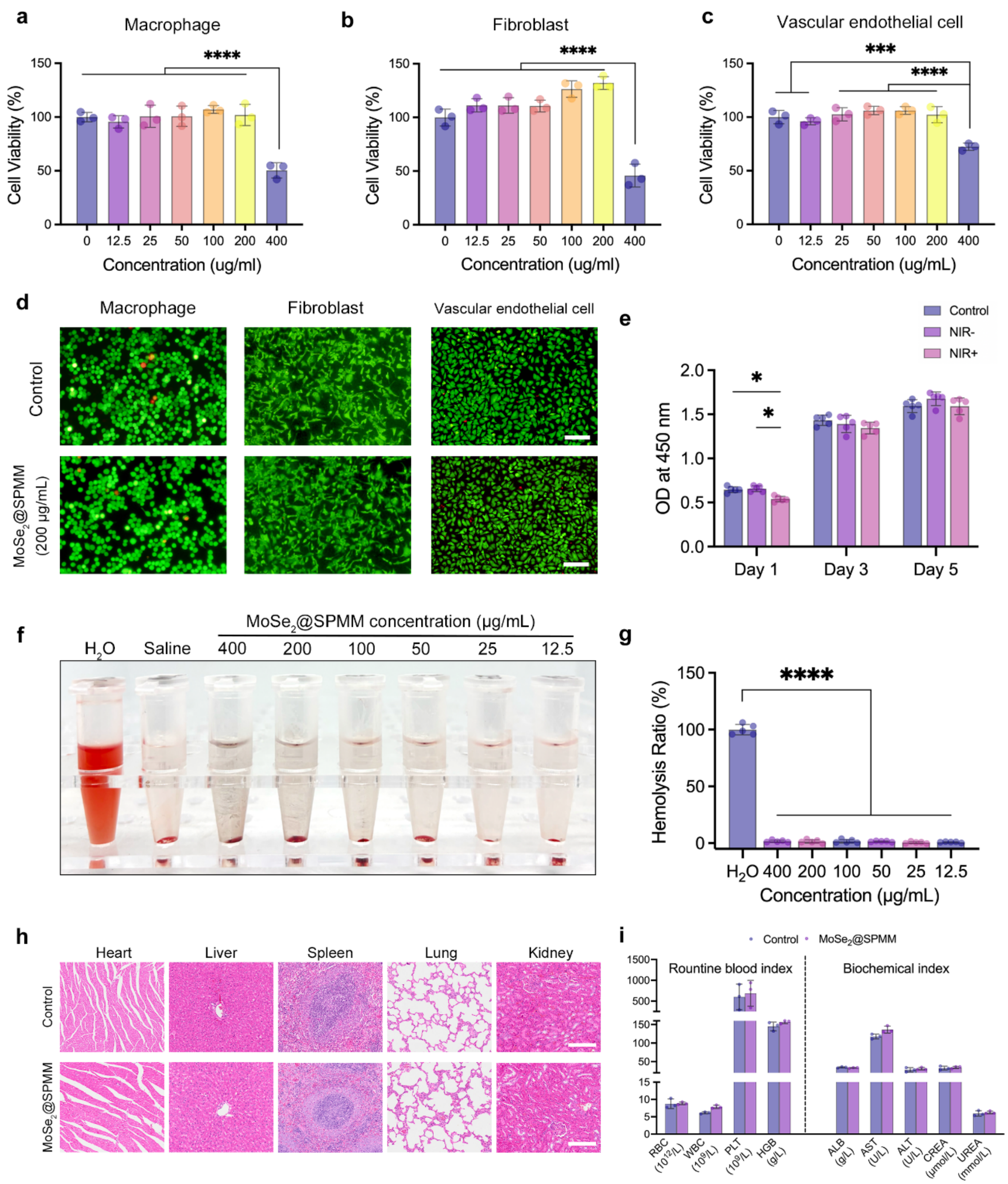

Figure S11 | Biosafety evaluation of $MoSe_2$@SPMM. Cell viability of macrophages (**a**), fibroblasts (**b**) and vascular endothelial cells (**c**) after incubation with different concentrations of $MoSe_2$@SPMM, assessed by CCK-8 assay. Data are presented as mean ± s.d. (n = 3 biological replications). **(d)** Live/dead staining of macrophages, fibroblasts and endothelial cells after treatment with $MoSe_2$@SPMM at 200 μg ml^{-1}. Live cells are shown in green and dead cells in red.

Scale bar, 100 μm. **(e)** Cell viability of macrophages treated with $MoSe_2$@SPMM with or without NIR irradiation on days 1, 3 and 5. Data are presented as mean ± s.d. (n = 5 biological replications). **(f)** Photographs showing haemolysis induced by different concentrations of $MoSe_2$@SPMM, with deionized water and saline used as positive and negative controls, respectively. **(g)** Quantitative analysis of haemolysis ratios. Data are presented as mean ± s.d. (n = 5 biological replications). **(h)** H&E staining of major organs following in vivo treatment with $MoSe_2$@SPMM. Scale bar, 100 μm. **(i)** Routine blood and serum biochemical analyses following in vivo treatment with $MoSe_2$@SPMM. Data are presented as mean ± s.d. (n = 3 biological replications). Statistical analysis was performed using one-way ANOVA. *, $p < 0.05$. ****, $p < 0.0001$.

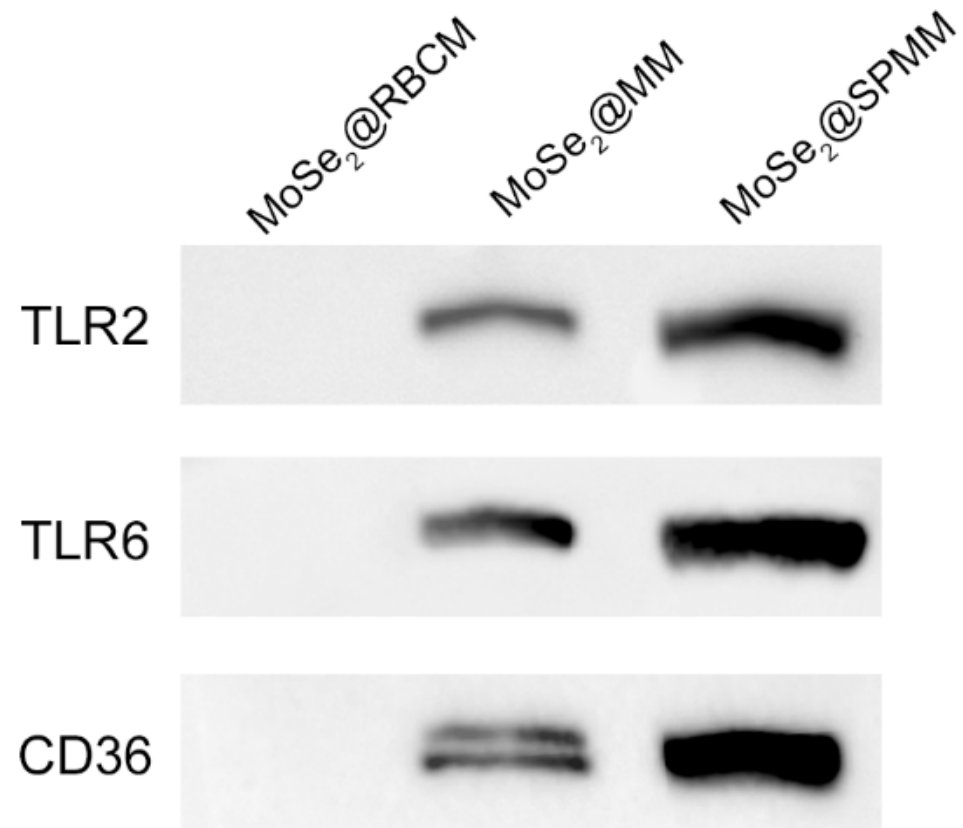

679 **Figure S12 | Western blotting analysis of TLR2, TLR6 and CD6 in the $MoSe_2$@RBCM, $MoSe_2$@MM, and $MoSe_2$@SPMM.** Experiments were repeated independently three times with similar results and a representative result is shown.

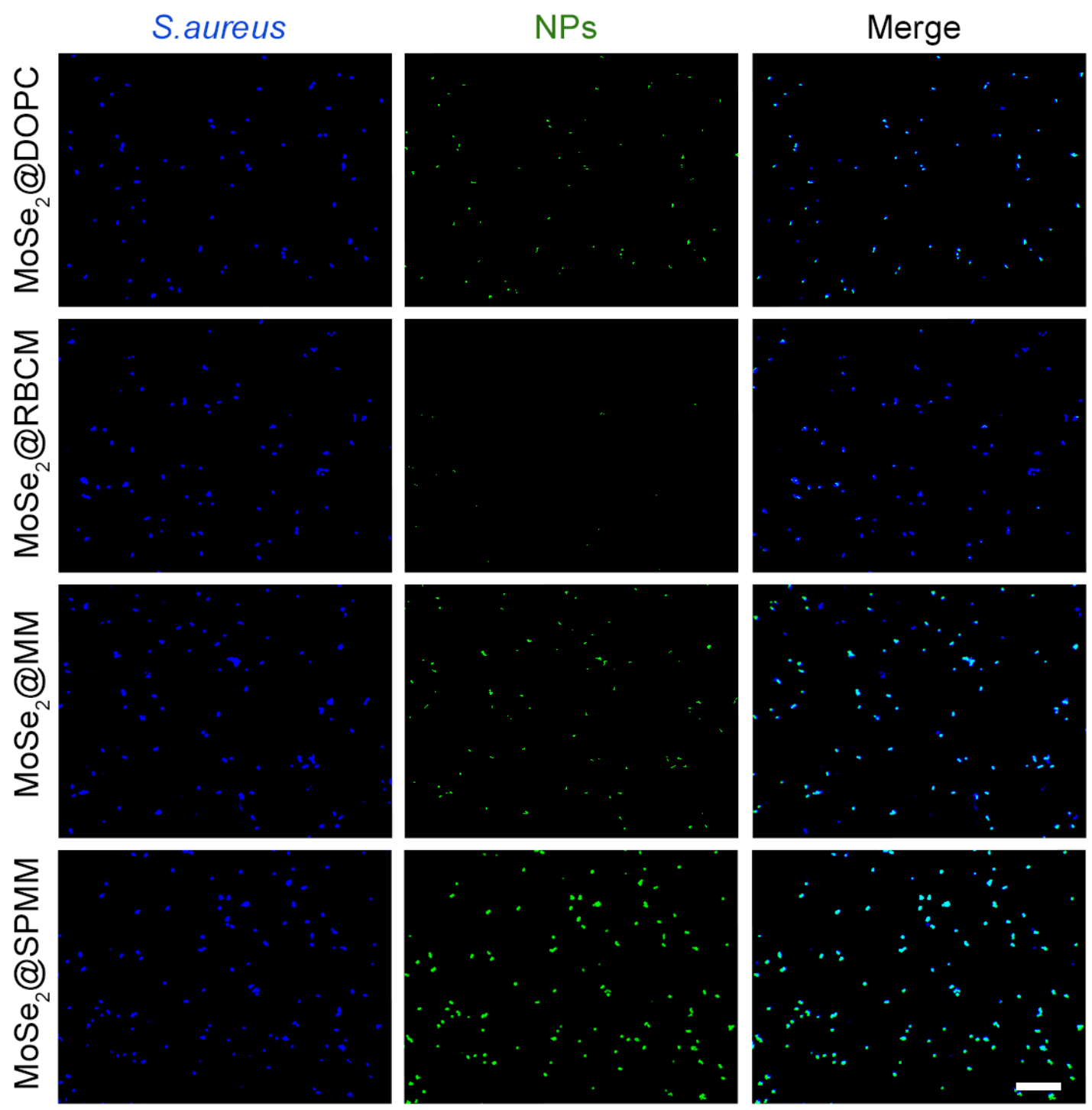

Figure S13 | Evaluation of *S. aureus* binding by $MoSe_2$@SPMM using $MoSe_2$@DOPC as a neutral lipid-coated control. CLSM images showing the binding of different nanoparticles with *S. aureus*. *S. aureus* was stained blue, and nanoparticles were labeled green. Scale bar, 20 μm.

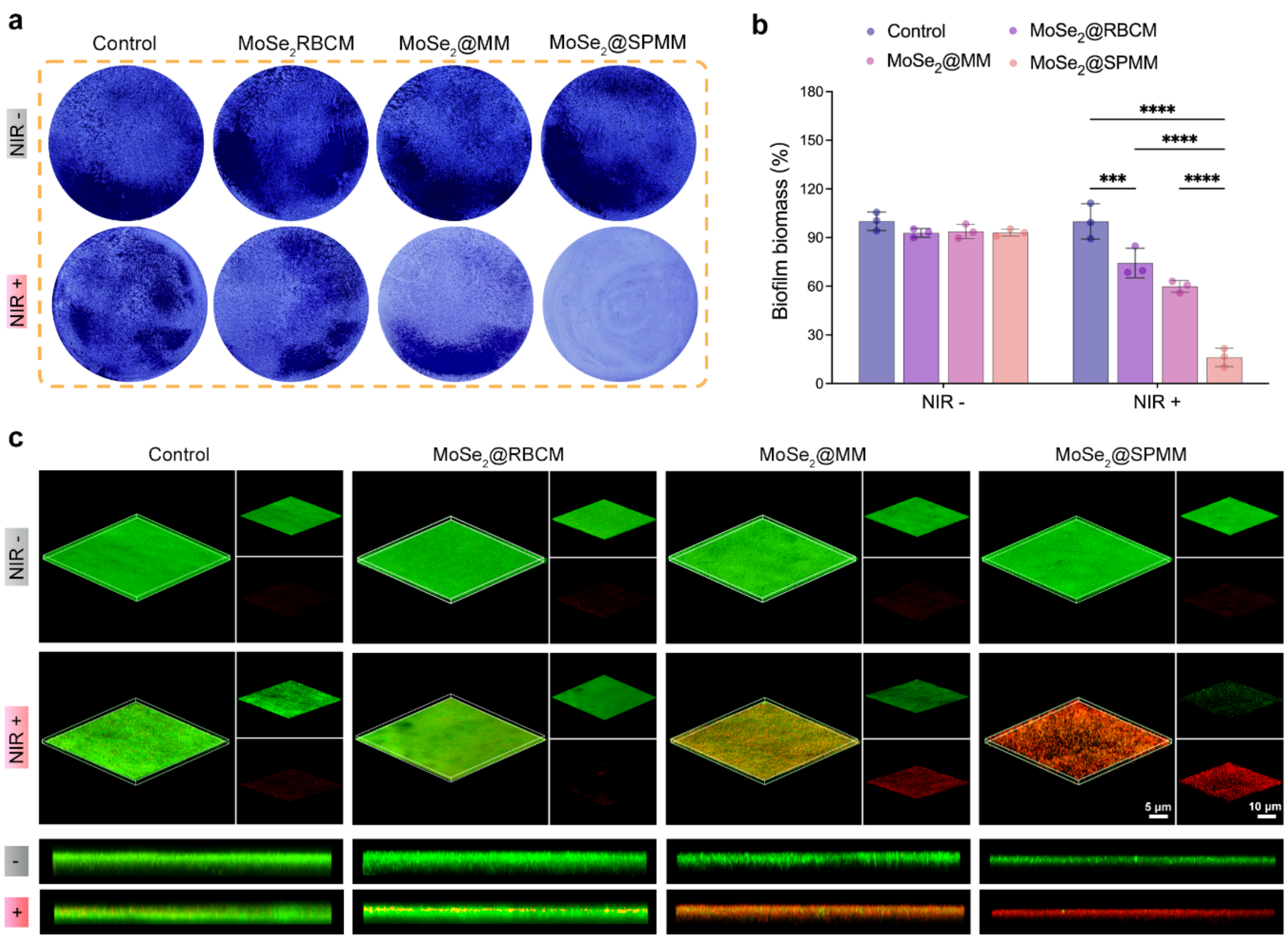

Figure S14 | $MoSe_2$@SPMM inhibits S. aureus biofilm formation under NIR irradiation. Crystal violet staining images showing biofilm formation under different treatments with or without NIR irradiation **(a)**, with quantification of biofilm biomass **(b)**. Data are presented as mean ± s.d. (n = 3 biological replicates). **(c)** CLSM images of bacterial biofilms under different treatments, with three-dimensional reconstruction and orthogonal cross-sectional analysis. Live bacteria are shown in green, whereas dead bacteria are shown in red. Scale bars, 5 μm for the merged three-dimensional view and 10 μm for the separated channel views. Statistical significance was calculated using one-way ANOVA with Tukey's post hoc test. Representative images in **a** and **c** are from three biologically independent experiments.

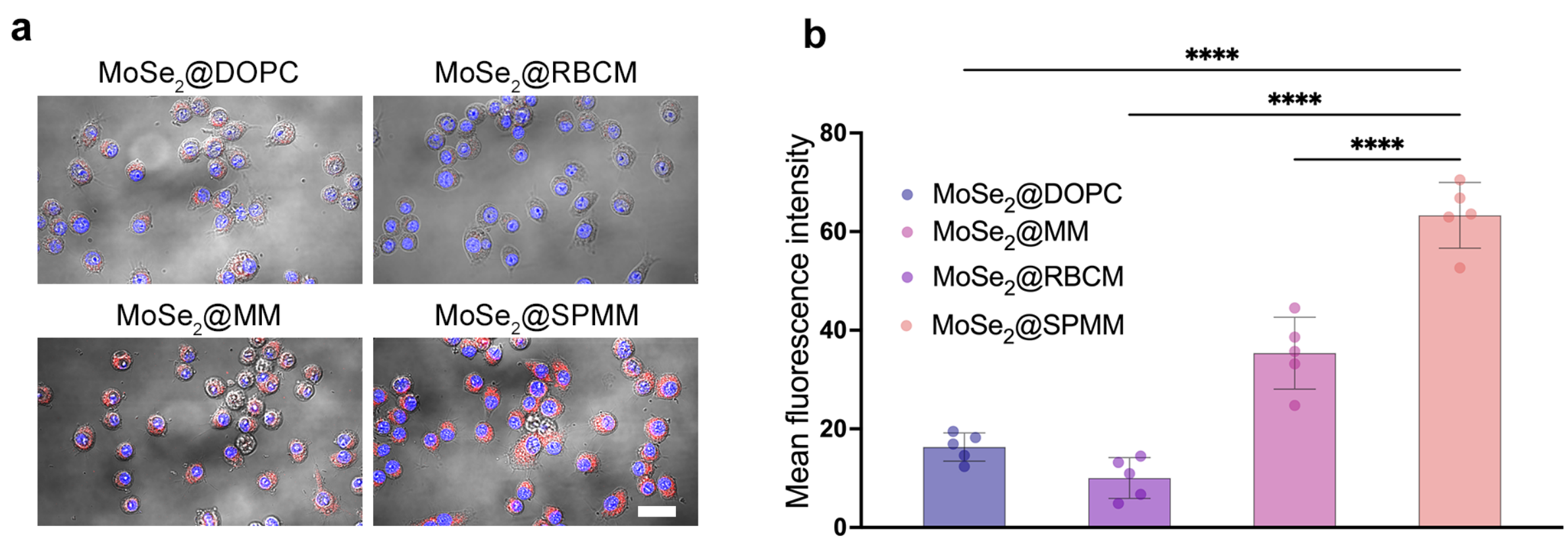

Figure S15 | Macrophage uptake of $MoSe_2$@SPMM compared with $MoSe_2$@DOPC. (a) CLSM images showing the uptake of different nanoparticles by RAW264.7 macrophages under OS conditions. Cell nuclei were stained blue, and nanoparticles were labeled red. Scale bar, 20 µm. (b) Quantification of nanoparticle uptake based on mean fluorescence intensity. Data are presented as mean ± s.d. ($n = 5$). Statistical significance was calculated using one-way ANOVA with Tukey's post hoc test. ****$p < 0.0001$.

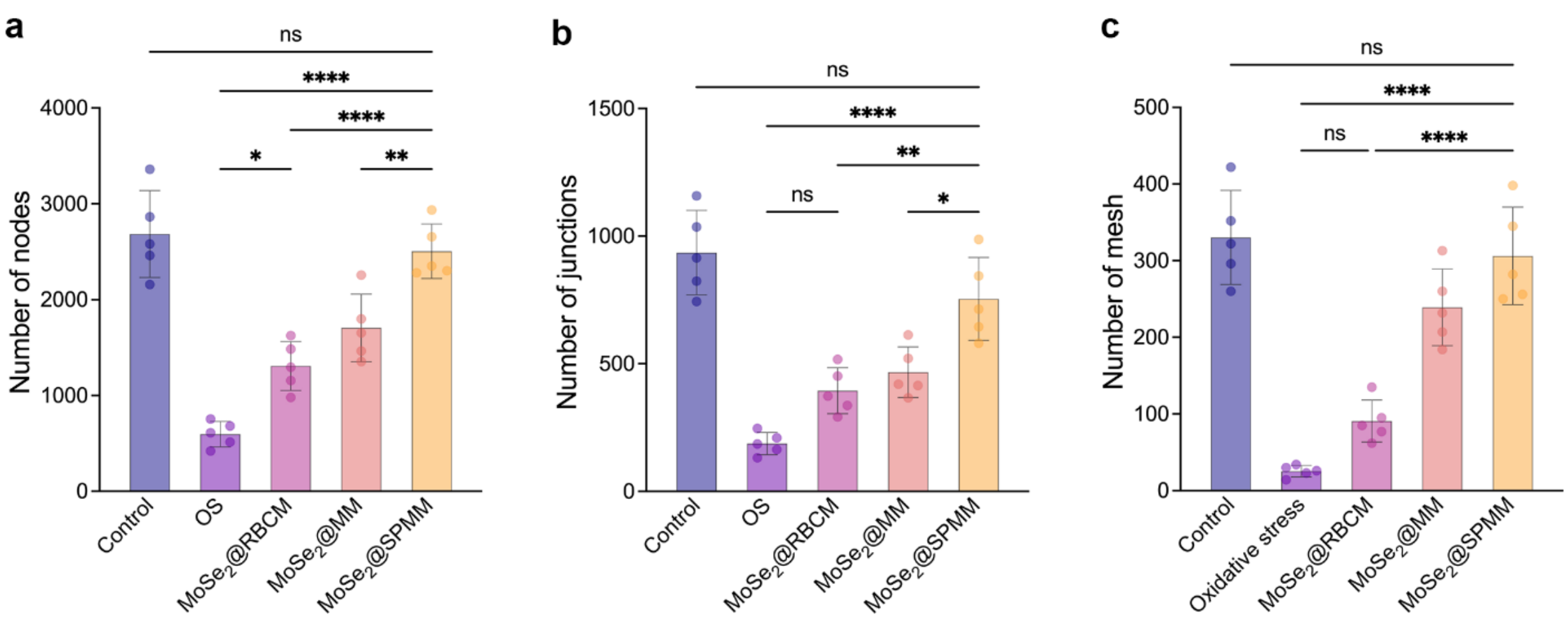

Figure S16 | Quantitative analysis of tube formation of HUVECs treated with conditioned medium from different groups. HUVECs were cultured with CCM from different treatment groups, and the tube-like structures were analyzed using ImageJ, including number of nodes **(a)** , junctions **(b)**, and mesh **(c)**. Data are presented as mean ± s.d. (n = 5 biological replicates). Statistical analysis was performed using one-way ANOVA. ns, not significant; $*p < 0.05$; $**p < 0.01$; $***p < 0.001$; $****p < 0.0001$.

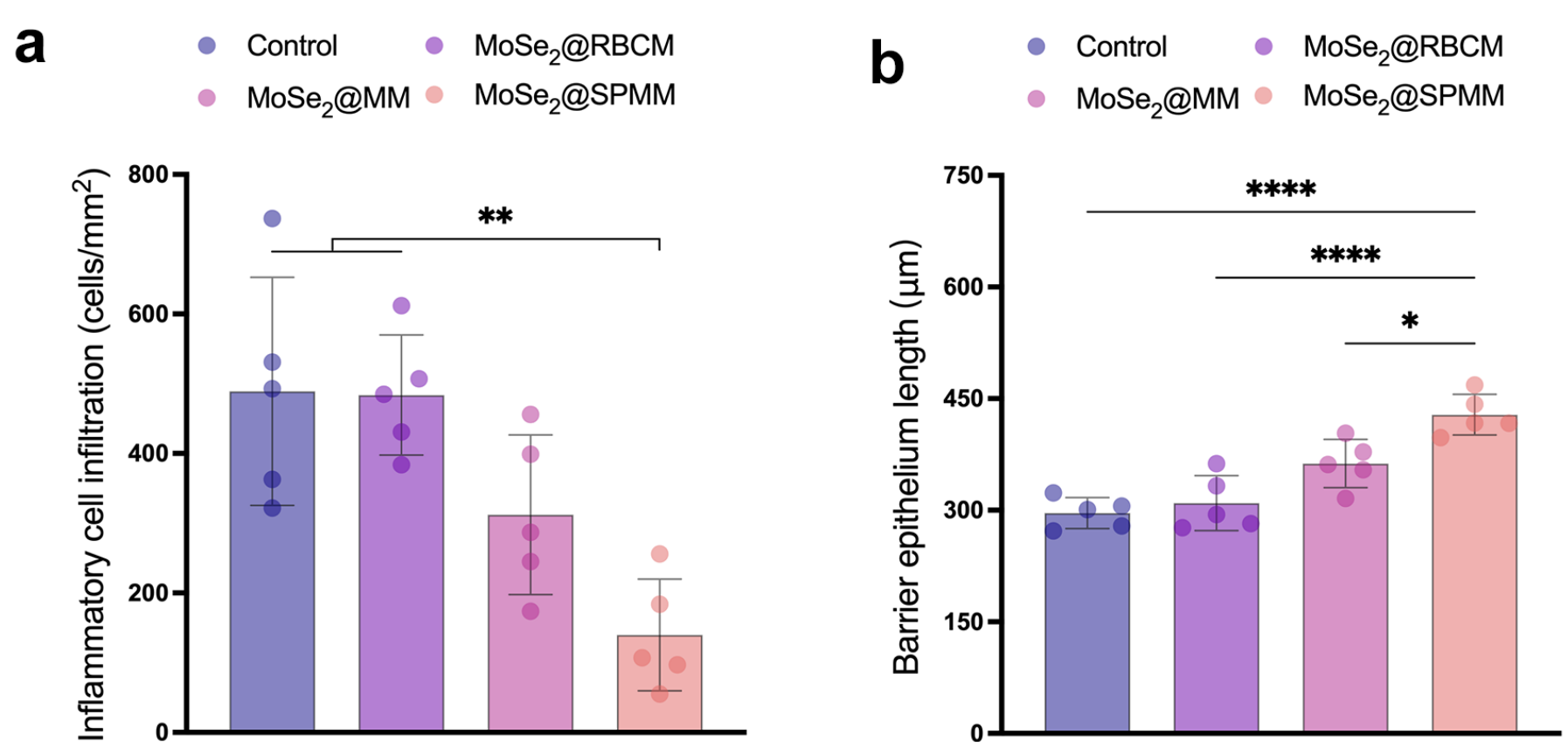

Figure S17 | Quantitative analysis of inflammatory cell infiltration and barrier epithelium formation in peri-implant soft tissues. (a) Quantification of inflammatory cell infiltration in peri-implant soft tissues based on H&E-stained sections. The number of inflammatory cells was calculated per unit area (cells/mm²). **(b)** Quantitative analysis of barrier epithelium length in peri-implant soft tissues. $MoSe_2$@SPMM treatment significantly reduced inflammatory cell infiltration and increased barrier epithelium length compared with other groups. Data are presented as mean ± s.d. (n = 5 biologically independent animals). Statistical significance was determined by one-way ANOVA with Tukey's multiple comparisons test. *$p < 0.05$, **$p < 0.01$, ****$p < 0.0001$.

Table S1. Primer sequences used for RT–qPCR analysis

Genes	Primers	Sequences
Tlr2	Forward	5'-AAGGGCAGCTCAGGATCTTT-3'
	Reverse	5'-CCATTGGATGTCAGCACCAG-3'
Tlr6	Forward	5'-AGTGAGTGGTGCCATTACGA-3'
	Reverse	5'-CAAATAAGTCCGCTGCGTCA-3'
Cd36	Forward	5'-TGGAACAGAGGCTGACAACT-3'
	Reverse	5'-ACAGGGTACGGAACCAAACT-3'
Tlr3	Forward	5'-CTTAGCACGGCTCTGGAAAC-3'
	Reverse	5'-CCTCAACTGGGATCTCGTCA-3'
Tlr4	Forward	5'-GCGTGAGACCAGAAAGCTG-3'
	Reverse	5'-CACCTGCAGTTCTGGGAAAC-3'
Tlr7	Forward	5'-CTCGAGGAAAGGGACTGGTT-3'
	Reverse	5'-GAGCCTCTGATGGGACAAGT-3'
Cd14	Forward	5'-AAAGATAACCGGCACCATGC-3'
	Reverse	5‘-GGGCAATGCTCAGTACCTTG-3'
Gapdh	Forward	5‘-GGACTCATGACCACAGTCCA-3'
	Reverse	5‘-TCAGCTCAGGGATGACCTTG-3'

References in supporting information

(1) Zhu, J.; Xie, R.; Gao, R.; Zhao, Y.; Yodsanit, N.; Zhu, M.; Burger, J. C.; Ye, M.; Tong, Y.; Gong, S. Multimodal Nanoimmunotherapy Engages Neutrophils to Eliminate Staphylococcus Aureus Infections. *Nature Nanotechnology* **2024**, *19* (7), 1032-1043. DOI: 10.1038/s41565-024-01648-8.
(2) Zhai, Y.; He, X.; Li, Y.; Han, R.; Ma, Y.; Gao, P.; Qian, Z.; Gu, Y.; Li, S. A Splenic-Targeted Versatile Antigen Courier: Ipsc Wrapped in Coalescent Erythrocyte-Liposome as Tumor Nanovaccine. *Science Advances* **2021**, *7* (35), eabi6326. DOI: doi:10.1126/sciadv.abi6326.
